# Base editing reveals an essential role for NANOG in human embryogenesis

**DOI:** 10.1038/s41586-026-10792-1

**Published:** 2026-06-25

**Authors:** Oliver J. Bower, Ana E. R. Orsi, Riley McMahon, Desislava Staneva, Josephine R. Blagrove, Kashish Singh, Claire S. Simon, Afshan McCarthy, Patricia Garcia, Valerie Shaikly, Mohamed Taranissi, Martin Wilding, Paul Serhal, Rabi A. Odia, Mina Vasilic, Meenakshi Choudhary, Athanasios Papathanasiou, Kay Elder, Phil Snell, Leila Christie, Mandana Arbab, David R. Liu, Mary Herbert, Katarina Harasimov, Kathy K. Niakan

**Affiliations:** 1 Loke Centre for Trophoblast Research, Department of Physiology, Development and Neuroscience, University of Cambridge, Cambridge, UK; 2 Human Embryo and Stem Cell Laboratory, The Francis Crick Institute, London, UK; 3 MRC Laboratory of Molecular Biology, Cambridge, UK; 4 Assisted Reproduction and Gynaecology Centre, London, UK; 5 Create Fertility, London, UK; 6 Centre for Reproductive and Genetic Health, London, UK; 7 Newcastle Fertility Centre, Newcastle upon Tyne Hospitals NHS Trust, Biosciences Institute, Newcastle University Centre for Life, Newcastle upon Tyne, UK; 8 Bourn Hall Clinic, Bourn, UK; 9 Rosamund Stone Zander Translational Neuroscience Center, Boston Children’s Hospital, Boston, MA USA; 10 Department of Neurology, F.M. Kirby Neurobiology Center, Boston Children’s Hospital, Harvard Medical School, Boston, MA USA; 11 Merkin Institute of Transformative Technologies in Healthcare, Broad Institute of Harvard and MIT, Cambridge, MA USA; 12 Department of Chemistry and Chemical Biology, Harvard University, Cambridge, MA USA; 13 Howard Hughes Medical Institute, Harvard University, Cambridge, MA USA; 14 Department of Anatomy and Developmental Biology, Monash Biomedicine Discovery Institute, Monash University, Melbourne, Victoria Australia; 15 Sidney Sussex College, Cambridge, UK; 16 Wellcome Trust – Medical Research Council Stem Cell Institute, Jeffrey Cheah Biomedical Centre, University of Cambridge, Cambridge, UK; 17 Epigenetics Programme, Babraham Institute, Cambridge, UK

**Keywords:** Genetic engineering, Embryology, Stem cells, Genomic instability, Embryonic stem cells

## Abstract

Understanding how the first cell lineages in human development are specified and maintained has fundamental importance and clinical implications for regenerative medicine, infertility and pregnancy loss. Although mouse models have provided valuable insights into transcription factors regulating early development, translating these findings to human embryos has been limited by ethical, technical and biological constraints. Functional studies of transcription factors in human embryos have been hindered by nuclease-based genome editing approaches that induce genotoxicity^1–3^. Here, to overcome this, we applied ABE8e adenine base editing^4,5^ to precisely target an exon splice donor site, resulting in a splicing defect and functional knockout of the developmental regulator *NANOG* in human embryos. This approach did not trigger genotoxicity and showed limited off-target editing. Loss of *NANOG* disrupts pluripotent epiblast specification and instead cells differentiate towards a primitive endoderm (yolk sac) or trophectoderm (placental) transcriptional programme. Retention of primitive endoderm differentiation in *NANOG*-edited human embryos reveals a functional compensation that is distinct from mouse, underscoring the importance of directly investigating human development. Our findings demonstrate an essential role for NANOG in human pluripotency and epiblast specification and highlight the utility of base editing for functional interrogation of human development.

## Abstract (web summary)

Base editing of *NANOG* in human embryos reveals its essential role in epiblast pluripotency, showing that loss of NANOG disrupts pluripotent epiblast specification and instead cells differentiate towards primitive endoderm and trophectoderm, which highlights base editing as a precise, non-genotoxic tool for studying human development.

## Main

The genetic regulation of lineage specification in the human embryo remains poorly understood, particularly the mechanisms by which key transcription factors establish and maintain the pluripotent embryonic epiblast. The homeodomain transcription factor NANOG is required for epiblast development in the mouse^6,7^. Moreover, NANOG contributes to stabilizing a pluripotency gene regulatory network that is required to maintain mouse and human embryonic stem cells (ESCs) in an undifferentiated state^8,9^. In human preimplantation embryos, NANOG expression is specifically restricted to the epiblast, the precursor population of the embryo proper, prior to the restriction of other epiblast-associated factors, such as OCT4 and SOX2^10–12^. The functional role of NANOG and the mechanisms by which it regulates epiblast specification and pluripotency maintenance in human embryos remain unclear partly owing to technical challenges associated with genetic perturbation in human embryos.

The use of CRISPR–Cas9 mutagenesis can trigger genomic instability, chromosomal rearrangements and unintended mutations at on-target sites due to error-prone resolution of DNA double-strand breaks^1–3^. Human zygotes are particularly vulnerable to these adverse effects due to inefficient DNA double-strand break repair, leading to high rates of karyotypic abnormalities after editing^1,3,13^. These limitations hinder the use of the originally described method of CRISPR–spCas9 for studying human embryogenesis and pose a significant barrier to its clinical translation.

## *NANOG* mutant hESCs lose stem cell identity

**Fig. 1:**
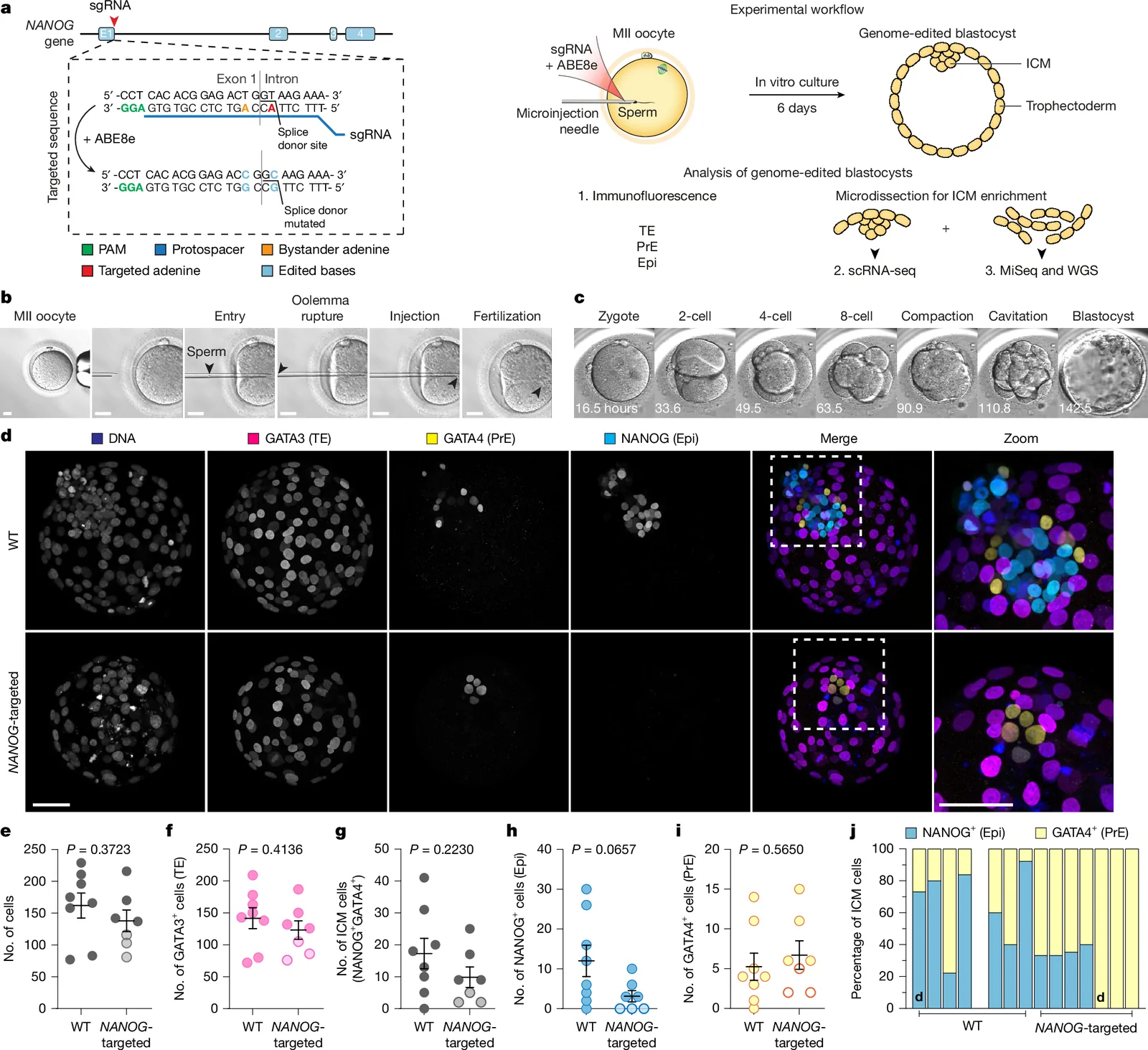
Loss of NANOG protein expression in human embryos following adenine base editing. **a**, Left, schematic of the human *NANOG* gene locus (top) and guide RNA (gRNA) (underlined in blue) located at the exon 1–intron 1 boundary. The NGG PAM sequence, target adenine and bystander adenine are highlighted. The most frequent sequence observed following ABE8e base editing results in a mutation at the splice donor site and a bystander allele. Right, schematic of base editing strategy in human embryos. Human oocytes were fertilized by ICSI. ABE8e and *NANOG*-targeting sgRNA were co-delivered using a microinjection needle. Embryos were cultured until the blastocyst stage (6 days post-fertilization). Embryos were processed for: (1) immunofluorescence analysis as blastocysts; (2) scRNA-seq following laser dissection to enrich for a polar region comprising epiblast and primitive endoderm cells of the ICM and polar trophectoderm; (3) mural trophectoderm further dissected for genotyping and low-pass whole-genome sequencing (WGS) for karyotype analysis. Epi, epiblast; ICM, inner cell mass; PrE, primitive endoderm; TE, trophectoderm. **b**, Bright-field images of the ICSI procedure. A microinjection needle containing sperm and base editing reagents was inserted into the oocyte; the oolemma was ruptured by gentle aspiration and the sperm and base editing reagents were deposited into the ooplasm. Black arrowheads indicate sperm position. **c**, Time-lapse microscopy of *NANOG* sgRNA ABE8e-edited embryos developing to blastocyst stage (*n* = 7 blastocysts for immunofluorescence, *n* = 12 blastocysts for single-cell collection). **d**, Immunofluorescence analysis of wild-type (WT) and *NANOG*-edited human blastocysts for DNA, GATA3, GATA4 and NANOG. **e**–**i**, Quantification of total cell number (**e**), trophectoderm (**f**), ICM (**g**), epiblast (**h**) and primitive ectoderm (**i**) in wild-type (*n* = 8) and *NANOG*-edited (*n* = 7) human blastocysts, analysed in two independent experiments. Dots represent individual embryos; error bars show mean ± s.e.m. Two-tailed Welch’s *t*-test. **j**, Proportions of each inner cell lineage in wild-type and *NANOG*-edited human blastocysts. Letters in bars indicate embryos shown in **d**. Scale bars, 50 µm. Source data

**Extended Data Fig. 1:**
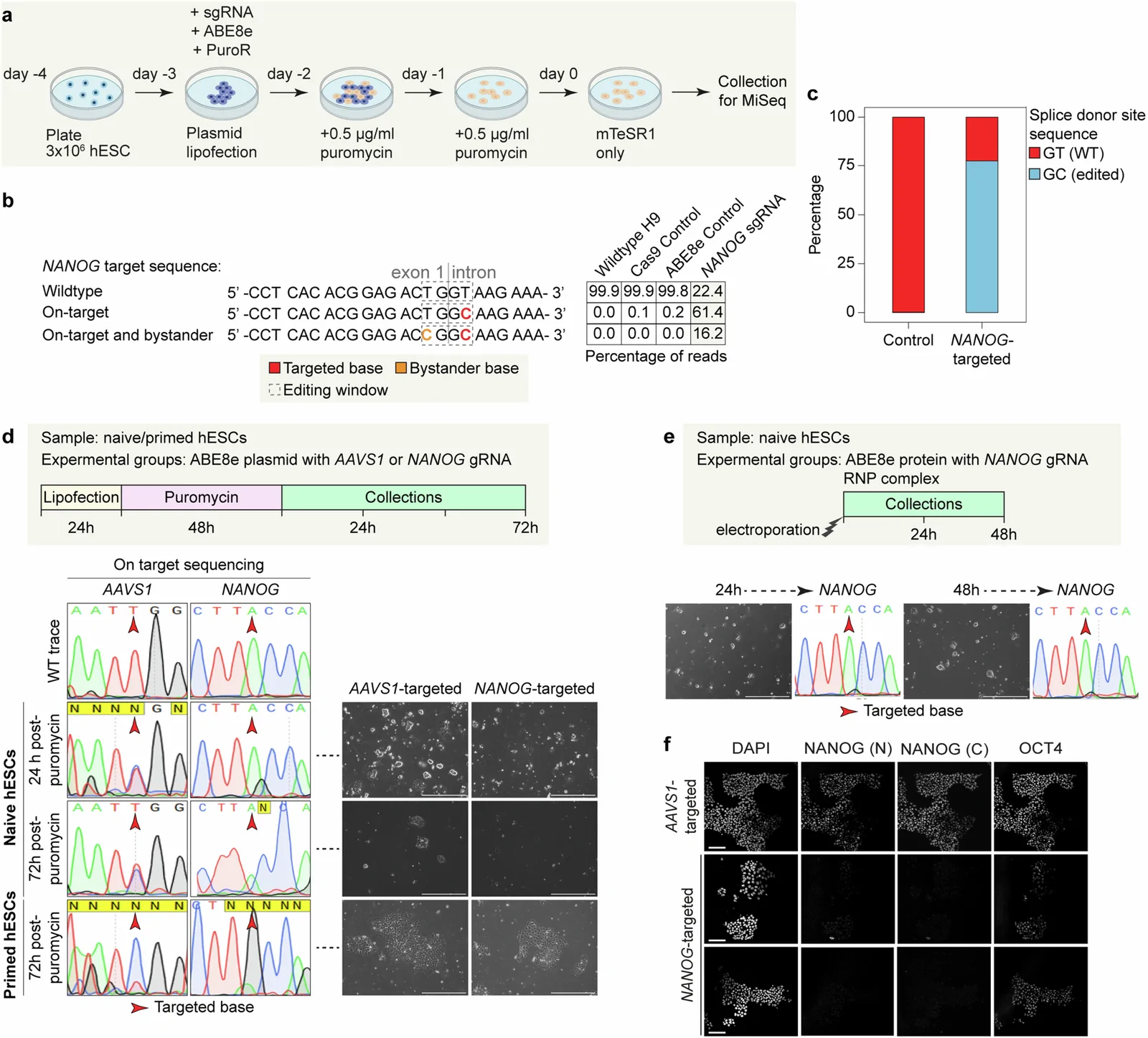
Optimisation of *NANOG* base editing in human embryonic stem cells. **a**, Schematic of the lipofection-based targeting strategy in human ESCs. 3 × 10^6^ cells were plated. After 24 h, cells were lipofected with plasmids containing ABE8e, puromycin resistance and a gRNA of interest. Cells were then selected with puromycin for 48 h, before DNA extraction and MiSeq analysis, or colony picking. **b**, Sequences and percentage observed incidences of edits at the *NANOG* locus in human ESCs. Wildtype, on-target only, and on-target plus bystander rates of editing are shown. **c**, Bar plots displaying the percentage of A•T > G•C single nucleotide change observed in human ESCs following transient transfection of ABE8e alone, or in combination with the *NANOG*-targeting sgRNA at the *NANOG* exon 1 splice donor site (n = 3 experimental replicates). Bulk population of cells were analysed post transfection and puromycin selection. **d**, Comparison of lipofection in naïve and primed human ESCs. Upper: Schematic of targeting strategy, left: representative Sanger sequencing traces of the *AAVS1* and *NANOG* target loci, right: representative brightfield images of *AAVS1-* and *NANOG*-edited human ESCs (n = 3 experimental replicates). **e**, Electroporation of *NANOG* in naïve human ESCs. Upper: Schematic of targeting strategy, lower: representative brightfield images and Sanger sequencing traces of edited cells (n = 3 experimental replicates). **f**, Immunofluorescence analysis of primed human ESCs 72 h following exposure to either *AAVS1* ABE8e or *NANOG* sgRNA ABE8e for DAPI (DNA), OCT4 and N-terminus and C-terminus NANOG antibodies (n = 3 experimental replicates). Scale bars, 100 µm. Source data

To overcome genotoxicity we used base editing, a precision genome editing technology that uses a deaminase enzyme fused to a Cas9 nickase protein to modify individual bases in the DNA of living cells without the generation of DNA double-strand breaks^14^. We selected the adenine base editor variant ABE8e for its enhanced catalytic activity following phage-assisted continuous evolution^5^. We designed a single guide RNA (sgRNA) to target the splice donor sites of *NANOG* (Fig. 1a), reasoning that it would induce a splicing defect leading to intron retention or exon skipping, ultimately producing mutant proteins^15^. To minimize the use of valuable human embryos, sgRNAs targeting *NANOG* were first screened in human ESCs (hESCs)^16^ (Extended Data Fig. 1a), where its function remains incompletely characterized and knockdown or knockout phenotypes are variable^11,17–21^.

**Extended Data Fig. 2:**
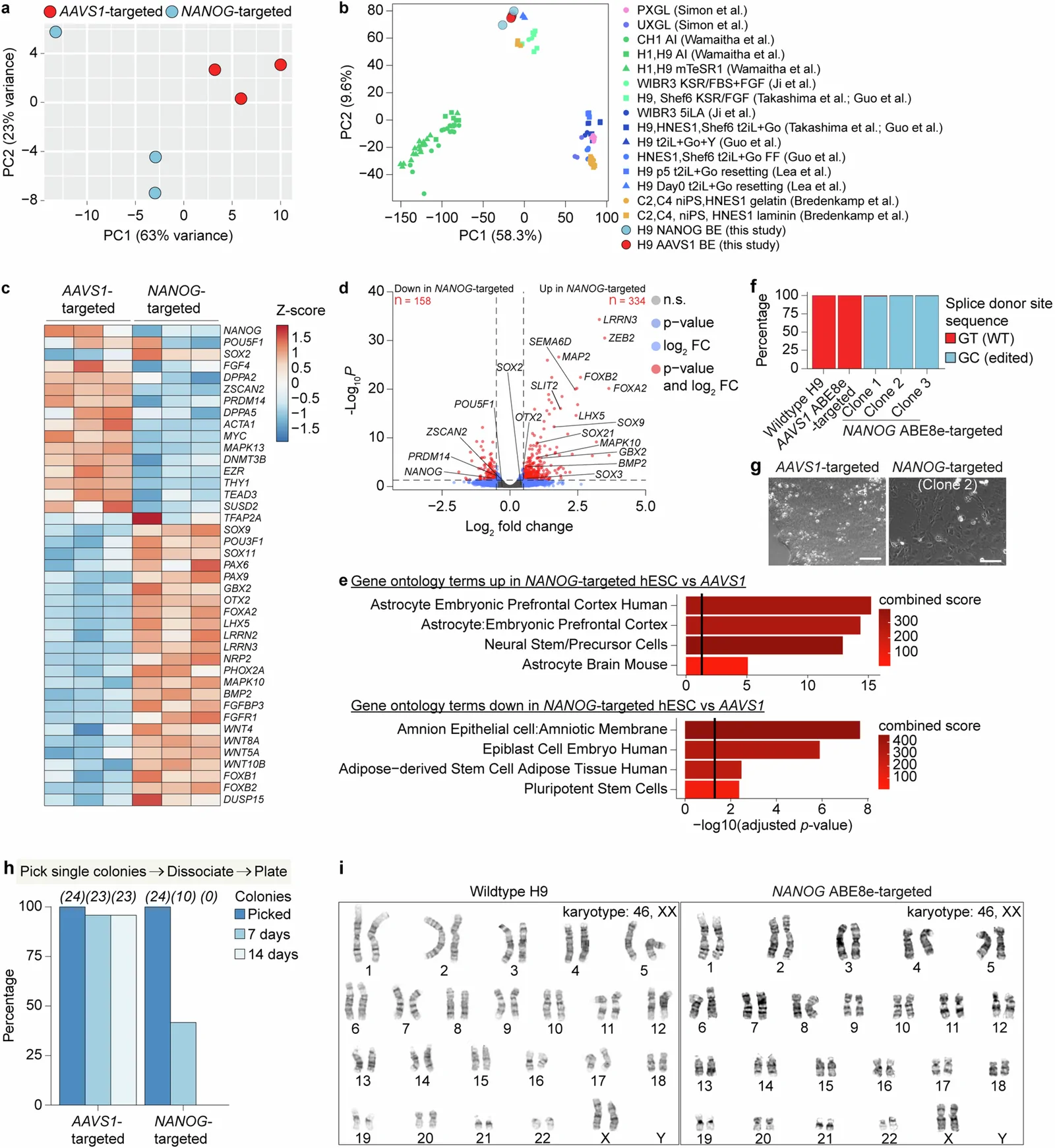
*NANOG* is required for the self-renewal of human embryonic stem cells and confers resistance to spontaneous differentiation. **a**, Principal component analysis of bulk RNA-sequencing data from primed human ESCs targeted with *AAVS1* ABE8e or *NANOG* sgRNA ABE8e (n = 3 independent replicates per group). **b**, Principal component analysis of *AAVS1*- and *NANOG*-edited primed human ESCs along with previously published datasets. **c**, Curated heatmap displaying the expression of markers associated with ESC pluripotency (top) and neuroectodermal differentiation (bottom), identified in the bulk RNA-sequencing analysis. Normalized expression shown as z-score. **d**, Volcano plot showing differentially expressed genes upregulated in *AAVS1* ABE8e- or *NANOG* sgRNA ABE8e-edited human ESCs. DESeq2 was used with adjusted p-value < 0.05 and log2FC > 1.0. Adjusted p-values were calculated using the two-sided Benjamini–Hochberg method to control for FDR across multiple tests). **e**, Gene ontology analysis of upregulated and downregulated terms in *NANOG*-edited versus *AAVS1*-edited primed human ESCs. Line depicts adjusted p-value of 0.05. Two-sided Kolmogorov-Smirnov test, adjusted p-values were calculated using the two-sided Benjamini–Hochberg method to control for FDR across multiple tests. **f**, Bar charts of amplicon sequencing analysis of surviving cells from single cell clones after targeting with *AAVS1* or *NANOG* (Representative, n = 2 and n = 3 *AAVS1* and *NANOG* colonies sequenced from n = 24 colonies across n = 2 experimental replicates). **g**, *AAVS1* and *NANOG* representative brightfield images of clones, n = 24 colonies across n = 2 experimental replicates. Scale bars, 100 µm. **h**, Bar chart quantifying the number of wells with viable human ESC colonies following *AAVS1* ABE8e and *NANOG* sgRNA ABE8e targeting, assessed at 7 and 14 days post colony picking (n = 24 colonies picked per condition across n = 2 experimental replicates). **i**, Representative karyotype analysis of wildtype H9 human ESCs compared to *NANOG*-edited cells (n = 3 experimental replicates). Source data

We selected an sgRNA targeting the splice donor site of exon 1 (Fig. 1a), converting the canonical -GT splice donor motif to -GC with 77.6% efficiency following transient transfection (Extended Data Fig. 1b,c). We targeted the *AAVS1* safe harbour locus as a control for delivery and activity and generated *AAVS1*-edited naive hESCs that represent an earlier epiblast-like state^20^ (Extended Data Fig. 1d). Despite multiple replicates and methods, *NANOG* editing in naive hESCs was inefficient and colonies were rapidly lost (Extended Data Fig. 1d,e). We therefore focused on the role of *NANOG* in primed hESCs that represent a peri- and early post-implantation epiblast-like state^22^, in which we were able to base-edit the gene and observed loss of protein (Extended Data Fig. 1a–e). We observed that *NANOG* ABE8e-edited cells displayed lower cell density compared with *AAVS1*-edited controls (Extended Data Fig. 1d,f). Bulk RNA sequencing (RNA-seq) of *NANOG*- and *AAVS1-*base-edited hESCs showed clustering with previously published primed hESCs and away from naive hESCs, indicating that transcriptome-wide, they retained their expected stem cell state (Extended Data Fig. 2a,b). *NANOG*-edited cells showed significant reduction in *NANOG*, *PRDM14*, *DPPA2*, *DPPA5*, *DNMT3B*, *SUSD2* and *ZSCAN2*, indicating a loss of pluripotency marker expression (Extended Data Fig. 2c,d and Supplementary Table 1). *NANOG* transcript may be downregulated owing to nonsense-mediated decay and/or differentiation of hESCs triggered by destabilization of pluripotency following *NANOG* perturbation. *NANOG*-edited cells upregulated neuroectoderm-associated molecular markers, including increased expression of *LRNN3*, *ZEB2*,* PAX6*, *PAX9*, *OTX2*, *SOX2* and *SOX9* (Extended Data Fig. 2c–e and Supplementary Table 1).

We next explored whether we could propagate *NANOG*-null mutant hESC lines. We performed single-cell colony cloning^23^ of *NANOG*- and *AAVS1*-base edited hESCs and cultured the resulting clones for 14 days (Extended Data Fig. 2f–h). After 14 days, 95.8% of *AAVS1*-edited clones formed viable, stable colonies, whereas only 41.7% of *NANOG*-edited clones formed colonies at day 7, with none persisting by day 14 (Extended Data Fig. 2h). Remaining *NANOG*-edited cells were sparse, highly differentiated and larger in size compared with *AAVS1*-edited controls (Extended Data Fig. 2g). Altogether, this demonstrates that adenine base editing of a splice donor site is an effective strategy for disrupting gene function. This also shows that *NANOG* is essential for maintaining pluripotency and self-renewal in hESCs, highlighting a critical role in hESC maintenance. The phenotype that we observed in hESCs contrasts with *Nanog*-null mouse ESCs that remain pluripotent, undergo indefinite self-renewal, express pluripotency-associated factors *Oct4*, *Sox2*, *Dppa3*, *Klf4*, *Esrrb* and *Zfp42*, and chimerically contribute to all somatic lineages and the germline^24,25^, which may reflect stem cell state, culture condition or species differences. The role of *NANOG* in hESCs is reminiscent of that reported in primed mouse epiblast stem cells, where it functions to inhibit somatic cell lineages, including neuroectoderm differentiation^26^, consistent with previous studies of hESCs in equivalent primed culture conditions^18^. G-band karyotyping showed that the *NANOG*-edited hESCs are karyotypically normal (Extended Data Fig. 2i) indicating that base editing does not lead to partial or whole chromosome gains or losses as previously reported for spCas9^1,3^.

## Reduced genotoxicity and off-target editing

**Extended Data Fig. 3:**
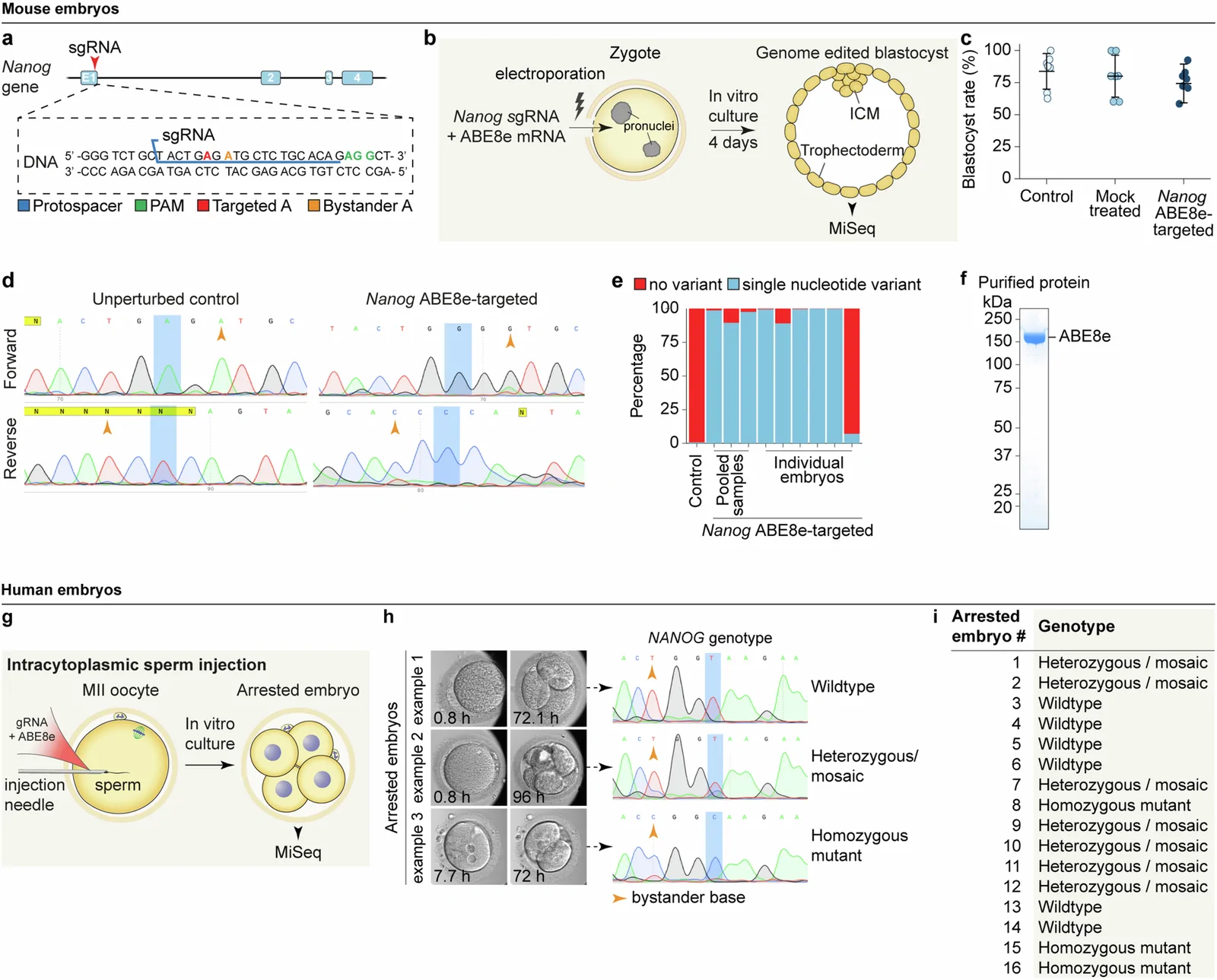
Optimisation of adenine base editing in mouse zygotes. **a**, Schematic of the mouse *Nanog* gene locus (top) and gRNA sequence site (bottom). The base editing sgRNA for *Nanog* (underlined in blue), NGG PAM sequence (gene), target (red) and bystander (orange) bases are highlighted. **b**, Schematic showing the process of targeting in mouse zygotes. Mouse zygotes were electroporated with *Nanog* sgRNA and ABE8e mRNA. Mouse blastocysts at 4.5 days post fertilisation were collected for DNA extraction and target amplicon MiSeq analysis at the on-target locus. **c**, Dotplot showing the blastocyst development rate in unperturbed controls, electroporated controls, and *Nanog* sgRNA ABE8e electroporated embryos (n = 7 independent experiments). Bars show mean and standard deviation. **d**, Representative examples of Sanger sequencing traces for unperturbed control or *Nanog* sgRNA ABE8e electroporated mouse embryos. **e**, Stacked bar charts showing target amplicon MiSeq analysis of pooled and individual blastocysts from targeting experiments. Pooled blastocysts are n = 10 embryos pooled into one lysis buffer for DNA extraction. **f**, SDS page gel of purified ABE8e protein, n = 1 protein purification. **g**, Schematic showing the experimental design for amplicon sequencing in arrested embryos. **h**, Sanger sequencing traces of representative arrested embryos harbouring wildtype, heterozygous/mosaic, and homozygous mutant *NANOG* sequences at the exon 1 splice donor site (n = 16 embryos total). **i**, Summary of observed editing rates in arrested human embryos targeted for *NANOG* ABE8e. Source data

We next determined the efficiency of ABE8e editing in mouse embryos. We designed a corresponding mouse sgRNA targeting the 3′ region of *Nanog* exon 1 (Extended Data Fig. 3a). Although this sgRNA could be used to optimize delivery methods in mouse embryos, the lack of a compatible protospacer adjacent motif (PAM) sequence at the exon–intron boundary prevented the targeting of the splice site to generate an equivalent splicing mutation. We electroporated mouse zygotes with ABE8e mRNA and *Nanog* sgRNA and collected blastocysts at 4.5 days post-fertilization for DNA extraction and next generation target amplicon sequencing (Extended Data Fig. 3b). We observed no significant difference in rates of blastocyst formation between *Nanog* ABE8e-edited embryos (*n* = 13) and unperturbed controls (*n* = 7) (Extended Data Fig. 3c). *Nanog*-edited embryos exhibited high frequency of detectable A•T>G•C edits at the on-target locus (Extended Data Fig. 3d,e). Single-nucleotide editing frequency was on average 82.4% in individual embryos (*n* = 6), with biallelic editing detected in 83% of embryos (5 out of 6). Pooled groups of 10 embryos showed a single-nucleotide editing frequency of 94.3% (*n* = 3 replicates, *n* = 30 embryos) (Extended Data Fig. 3e). In keeping with previous reports, we observed silent bystander mutation of neighbouring bases, targeting a proximal adenine base pair because it falls within the editing window of ABE8e^27^ (Extended Data Fig. 3d). To further optimize our delivery approach, we performed purification of ABE8e protein for human embryo experiments (Extended Data Fig. 3f).

We next proceeded to target human embryos and gametes donated for research. We used a co-injection method that has previously been shown to reduce mosaicism and increase the efficiency of CRISPR–Cas9-mediated editing^28^. Cryopreserved MII-stage oocytes and sperm were thawed. Using intracytoplasmic sperm injection (ICSI) sperm was co-microinjected with ABE8e mRNA or purified protein and *NANOG* sgRNA (Fig. 1a,b and Extended Data Fig. 3g). Based on our prior experience, approximately 50% of human embryos form blastocysts following microinjection of spCas9 protein^16^, consistent with typical developmental outcomes observed with in vitro fertilization in clinical settings^29^. Consistent with this frequency, 43% of *NANOG*-edited embryos initiated blastocyst formation and expansion (*n* = 12 out of 28 embryos) (Fig. 1c). We confirmed NANOG protein loss in the *NANOG*-edited embryos by immunofluorescence analysis (Fig. 1d). Co-staining and quantification of trophectoderm and primitive endoderm markers GATA3 and GATA4 showed the retention of these extraembryonic lineages despite the loss of *NANOG* (Fig. 1e–j). To maximize the information gained from embryos used in this study, we also analysed arrested human embryos to evaluate editing frequency (Extended Data Fig. 3g–i). Sequencing of embryos that arrested prior to the blastocyst stage revealed an editing frequency of 62.5% (*n* = 10 out of 16 embryos) (Extended Data Fig. 3i). However, given that arrested human embryos are developmentally compromised and frequently carry karyotypic abnormalities^30^, this editing rate may not be representative of embryos that are capable of developing beyond this stage.

**Fig. 2:**
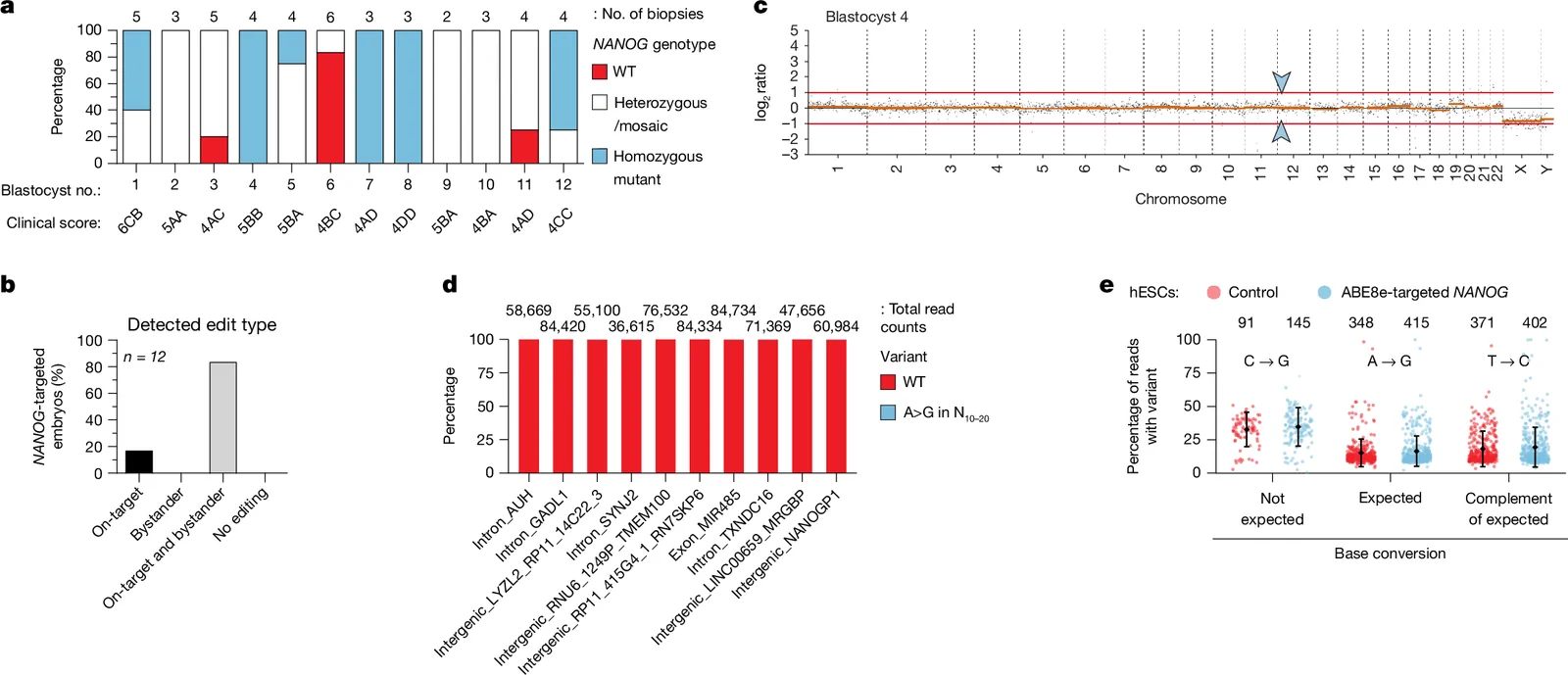
Minimal evidence of off-target editing and no obvious genotoxicity following base editing of *NANOG* in human embryos. **a**, Bar chart of observed A•T>G•C editing at the *NANOG* exon 1 splice donor site based on Sanger sequencing of trophectoderm biopsies from 12 *NANOG*-edited blastocysts (*n* = 46 biopsies). Clinical scoring of each blastocyst: 4, fully expanded blastocyst; 5, hatching (trophectoderm breaking through zona pellucida); 6, fully hatched blastocyst; A, excellent (many tightly packed cells); B, good (several loosely grouped cells); C, fair (very few cells); and D, poor (degenerate and/or fragmented cells). **b**, Proportions of *NANOG*-edited blastocysts that had edits at the on-target base, a bystander base, both bases, or no editing. **c**, Representative whole-genome sequencing analysis of trophectoderm biopsies from *NANOG*-edited blastocyst 4, with blue arrowheads indicating the *NANOG* locus on chromosome 12 (*n* = 5 blastocysts, *n* = 23 biopsies). **d**, Bar chart of observed A•T>G•C editing at 10 in silico predicted off-target sites in pooled biopsies from edited blastocysts (*n* = 10 embryos, 30 biopsies in total). **e**, Jitter plots of observed C>G (not expected), A>G (expected) and T>C (reverse complement of expected) ABE8e editing across the transcriptome in *NANOG*-edited hESCs as determined by bulk RNA-seq (*n* = 3 experimental replicates; numbers of RNA-seq loci is noted in the graph). Bars show mean ± s.d. Source data

**Extended Data Fig. 4:**
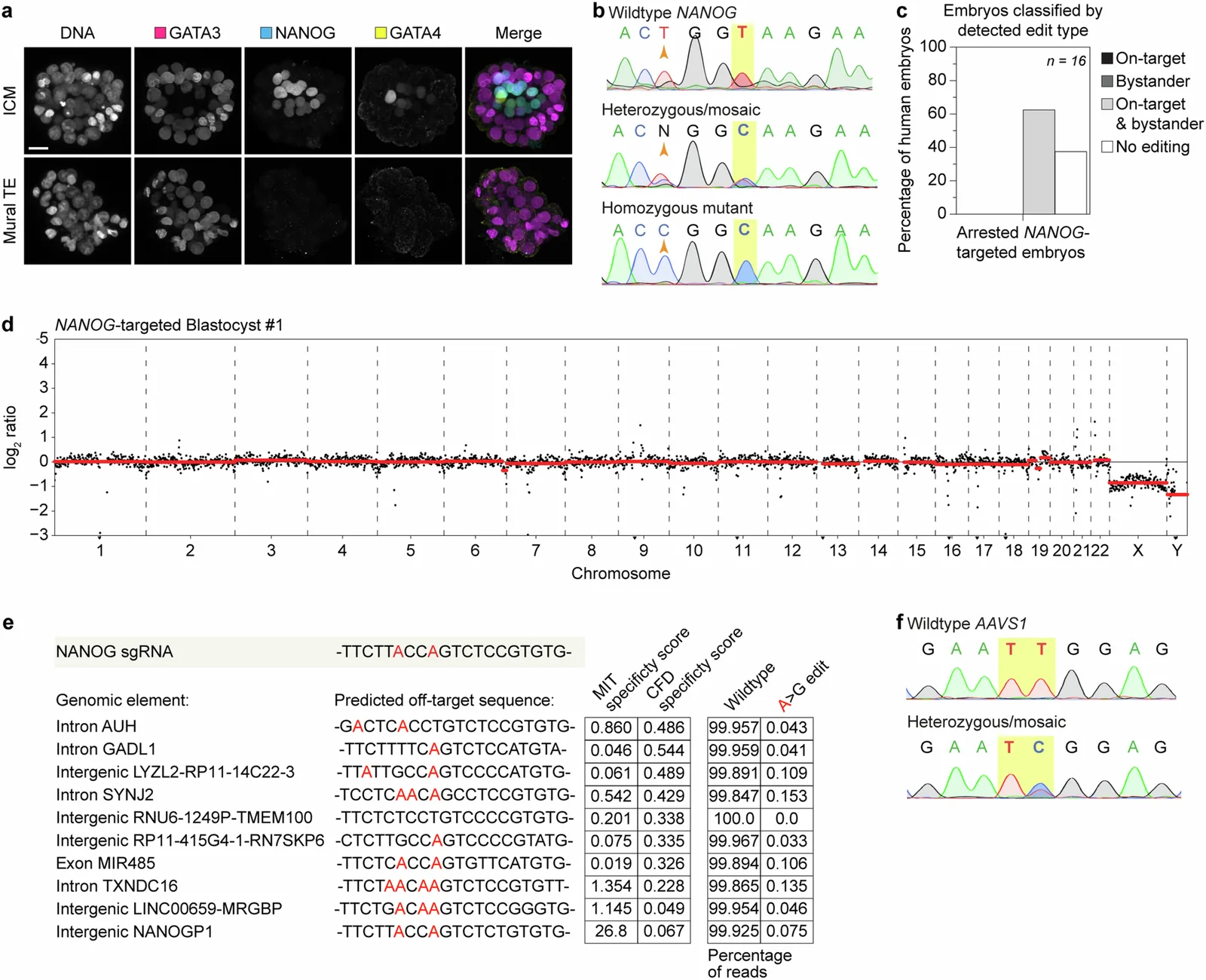
Further characterisation of base editing in human embryos. **a**, Representative immunofluorescence analysis following laser microdissection to separate polar region comprising inner cell mass cells (epiblast and primitive endoderm) and polar trophectoderm from mural trophectoderm in blastocyst stage embryos (n = 4 dissected blastocysts). Scale bar, 20 µm. **b**, Representative Sanger sequencing traces of observed wildtype, heterozygous/mosaic, and homozygous mutant editing observed in *NANOG*-edited embryos in Fig. 2. Target base highlighted in yellow, bystander base shown with orange arrowhead. **c**, Bar chart indicating proportion of *NANOG*-edited arrested embryos that show editing at the on-target base only, bystander base only, both, or neither. **d**, Low pass whole genome sequencing analysis of biopsies from *NANOG*-edited blastocysts, supplemental to Fig. 2c. **e**, Sequencing analysis and quantification of *in-silico* predicted off-targets shown in Fig. 2d, potential target A bases used for analysis are indicated in red. MIT and CFD specificity scores are shown. **f**, Genotyping of biopsies from *AAVS1*-edited blastocysts. Target base highlighted in yellow. Source data

As blastocyst formation was not compromised, we next sought to investigate the consequences of *NANOG* loss on human preimplantation embryos (Fig. 1a). Blastocyst quality was scored using clinical embryology grading, which showed that compared with controls *NANOG*-edited blastocysts had a poorer quality inner cell mass (ICM) (Fig. 2a). *NANOG*-edited blastocysts were laser microdissected to enrich for a polar region comprising epiblast, primitive endoderm and polar trophectoderm separated from mural trophectoderm cells (Fig. 1a and Extended Data Fig. 4a). Mural trophectoderm was further microdissected and genotyped, whereas the polar regions were dissociated for subsequent single-cell RNA sequencing (scRNA-seq) and transcriptome analysis (Fig. 1a). We observed on-target T•A>C•G editing at the *NANOG* exon 1 splice donor site in 84.8% of trophectoderm biopsies from edited blastocysts (*n* = 12 embryos, 39 out of 46 biopsies) (Fig. 2a and Extended Data Fig. 4b). Biallelic editing was variable among biopsies, with homozygous mutation in 37.0% of biopsies and either heterozygous or mosaic mutations in 47.8% of biopsies (*n* = 17 and *n* = 22 out of 46 biopsies, respectively) (Fig. 2a). As previously described, we also observed bystander complement T•A>C•G editing of a thymine located three base pairs upstream (5′) of the target sequence in 10 out of 12 (83.3%) blastocysts and 10 out of 16 (62.5%) arrested embryos (Fig. 2b and Extended Data Fig. 4b,c). Exclusive on-target base editing was observed in 2 out of 12 blastocysts (16.7%) (Fig. 2b). The bystander base change is a silent mutation that does not alter the amino acid sequence of *NANOG*.

To assess aneuploidy and chromosomal loss following editing, we performed whole-genome sequencing on trophectoderm biopsies (Fig. 2c and Extended Data Fig. 4d). No loss of chromosomal material was detected at the on-target loci or the top 5 predicted off-target sites in any of the biopsies analysed (*n* = 23 biopsies) (Fig. 2c and Extended Data Fig. 4d). *NANOG* sgRNA is predicted to edit an off-target *NANOG* pseudogene at an intergenic region, and other loci were predicted in silico to have low rates of editing (Fig. 2d and Extended Data Fig. 4e). The majority of predicted off-target sites were either in introns or intergenic regions with a single exonic region for a microRNA *MIR485* predicted (Extended Data Fig. 4e). We performed next generation amplicon sequencing of 10 predicted off-target sites in edited blastocysts (*n* = 10 embryos, 30 biopsies in total) and observed 0.15% or less edits (Fig. 2d and Extended Data Fig. 4e).

Previous versions of deaminase base editors have been suggested to lead to RNA off-target edits, particularly using the cytidine deaminase base editor^31–34^. To assess possible RNA off-target edits following ABE8e editing, we transfected hESCs and collected cells for genome-wide total RNA-seq 48 h following puromycin selection (Fig. 2e). We applied a previously published pipeline to determine edits in RNA-seq reads^31^ and observed A-to-G editing rate at all sites increased by 1.2% in ABE8e *NANOG*-edited cells compared with controls (*n* = 348 loci in control, *n* = 415 loci in ABE8e *NANOG*-edited) (Fig. 2e). Notably, we observed similar percentages of base changes at other nucleotides not associated with adenine base editing conversion, for example 1.6% increase of C-to-G base conversion in ABE8e *NANOG*-edited cells compared with controls (*n* = 91 loci in control, *n* = 145 loci in ABE8e *NANOG*-edited), indicative of technical DNA polymerase or sequencing error rates. Overall, these data demonstrate effective base editing in human embryos, absence of genotoxicity, low off-target editing and human blastocyst initiation despite the loss of *NANOG*.

## NANOG is essential for epiblast specification

**Extended Data Fig. 5:**
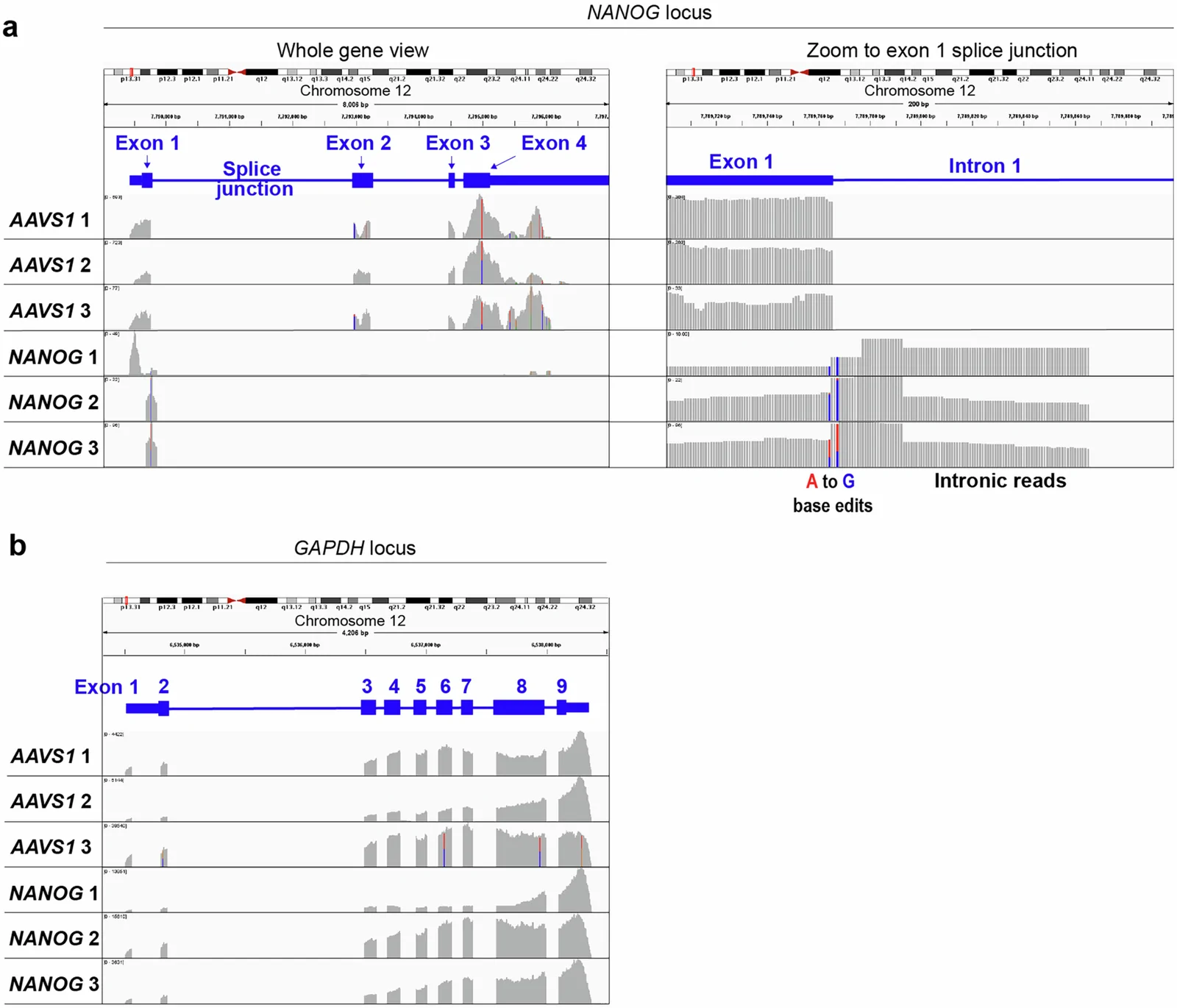
Splice defects in *NANOG*-edited human embryo single cells. **a**, Representative IGV genome browser images showing scRNAseq alignments at the *NANOG* locus from exon 1 until the 3’ untranslated region (left) and focused on the exon 1-intron 1 splice donor site (right) for three cells from *AAVS1*-edited and three cells from *NANOG*-edited blastocysts. The right panel shows A to G edits in blue and intronic reads in *NANOG*-edited cells only. **b**, The same representative cells as **a** display correct splicing at the *GAPDH* locus in all cells.

The retention of GATA4 in *NANOG*-edited human embryos was unexpected because *Nanog*-null mutant mouse embryos are defective in primitive endoderm specification, including the absence of GATA4^35,36^. To investigate the transcriptome-wide effects of *NANOG* loss for human lineage specification, we performed scRNA-seq on dissociated polar regions. We isolated biopsies from 12 *NANOG*-edited embryos and single cells (359 cells) from 11 out of 12 of these because one biopsy lysed prior to scRNA-seq. We compared these to cells isolated from two *AAVS1*-edited controls (42 cells from 2 polar regions) (Extended Data Fig. 4f) and previously published reference transcriptome datasets^37–39^. Genotyping of biopsied embryos indicated homozygous mutant, heterozygously or mosaically edited, or wild-type alleles at the *NANOG* locus (Fig. 2a). Although these groupings informed further analysis, trophectoderm biopsies represent only a portion of the embryo and may not reflect the genotype of the embryo as a whole. In *NANOG*-edited cells, scRNA-seq reads exhibited GT-to-GC mutation at the *NANOG* splice donor site, aberrant intron-retaining transcripts following exon 1 and absence of mapping to subsequent exons, suggesting a splicing defect (Extended Data Fig. 5). By contrast, in *AAVS1*-edited controls, scRNA-seq reads mapped to the 3′ untranslated region and all *NANOG* exons, with evidence of correct splicing across exonic regions (Extended Data Fig. 5). Reads mapping to the *GAPDH* locus in both the *NANOG*- and *AAVS1*-edited cells showed correct splicing and reads mapping to only exons.

**Fig. 3:**
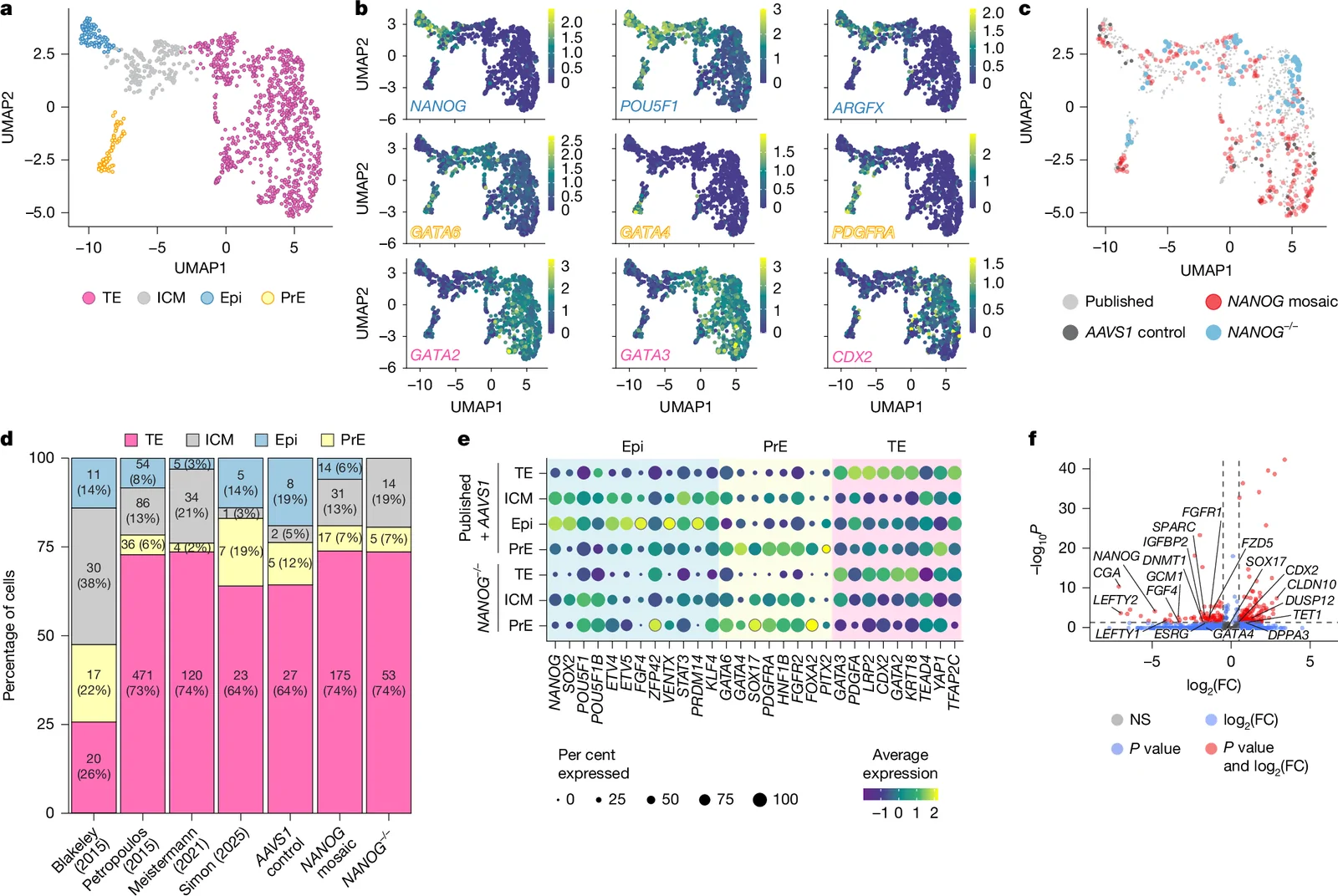
*NANOG*-edited human embryo cells do not specify embryonic epiblast, preferentially contributing to extraembryonic lineages. **a**, Uniform manifold approximation and projection (UMAP) integrated clustering of the experimental scRNA-seq data generated in this study with published reference datasets^37–39^ (*n* = 359 cells from 11 blastocysts, integrated with *n* = 888 cells from previously published datasets). Cells are coloured by lineages based on marker expression in **b** and the Seurat FindNeighbors and FindClusters functions. **b**, UMAP feature plots showing relative expression of molecular markers enriched in lineages: epiblast (*NANOG*,* POU5F1* and* ARGFX*), primitive endoderm (*GATA6*,* GATA4* and* PDGFRA*), ICM (*GATA6* and *POU5F1*) and trophectoderm (*GATA2*,* GATA3* and* CDX2*). **c**, UMAP annotated according to experimental condition: *AAVS1*-edited embryos or *NANOG*-edited embryos that showed mosaic or homozygous editing based on trophectoderm biopsies in Fig. 2a. **d**, Stacked bar plots indicating the number and proportion of cells from each embryo experimental group mapped to each lineage. Published controls are shown as a comparison^37–39,44^. All data plotted underwent similar laser-dissected enrichment for the polar region and SMART-seq based RNA-seq. **e**, Dot plot summarizing relative expression of lineage-associated molecular markers across the published reference and *AAVS1*-edited embryos versus *NANOG*-edited homozygous embryo datasets. The percentage of cells and average expression are shown. **f**, Volcano plot with genes that are significantly differentially expressed between *NANOG*-edited homozygous embryos and *AAVS1*-edited embryos. Significantly differentially expressed genes (MAST test with adjusted *P* value < 0.05 and log_2_(fold change) > 1.0 (red)). Adjusted *P* values were calculated using the two-sided Bonferroni correction method to control for multiple tests. FC, fold change; NS, not significant. Source data

We integrated these data with previously published human preimplantation datasets^37–39^ to benchmark cell populations against established and well-characterized embryonic lineages (Fig. 3a–c). Following integration, four main cell populations were identified based on expression of lineage-associated molecular markers: trophectoderm (*GATA2*, *GATA3* and *CDX2*), ICM (*GATA6* and *POU5F1*), epiblast (*NANOG*, *POU5F1* and *ARGFX*) and primitive endoderm (*GATA6*, *GATA4* and *PDGFRA*) (Fig. 3a–c). Cells from all three *NANOG*-null mutant edited embryos (blastocysts 4, 7 and 8) clustered predominantly with the ICM, trophectoderm and primitive endoderm groups, whereas cells from heterozygous or mosaic embryos (blastocysts 1, 2, 3, 5, 6, 9, 10 and 11) clustered with ICM, epiblast, trophectoderm and primitive endoderm control cells (Fig. 3a–d).

**Fig. 4:**
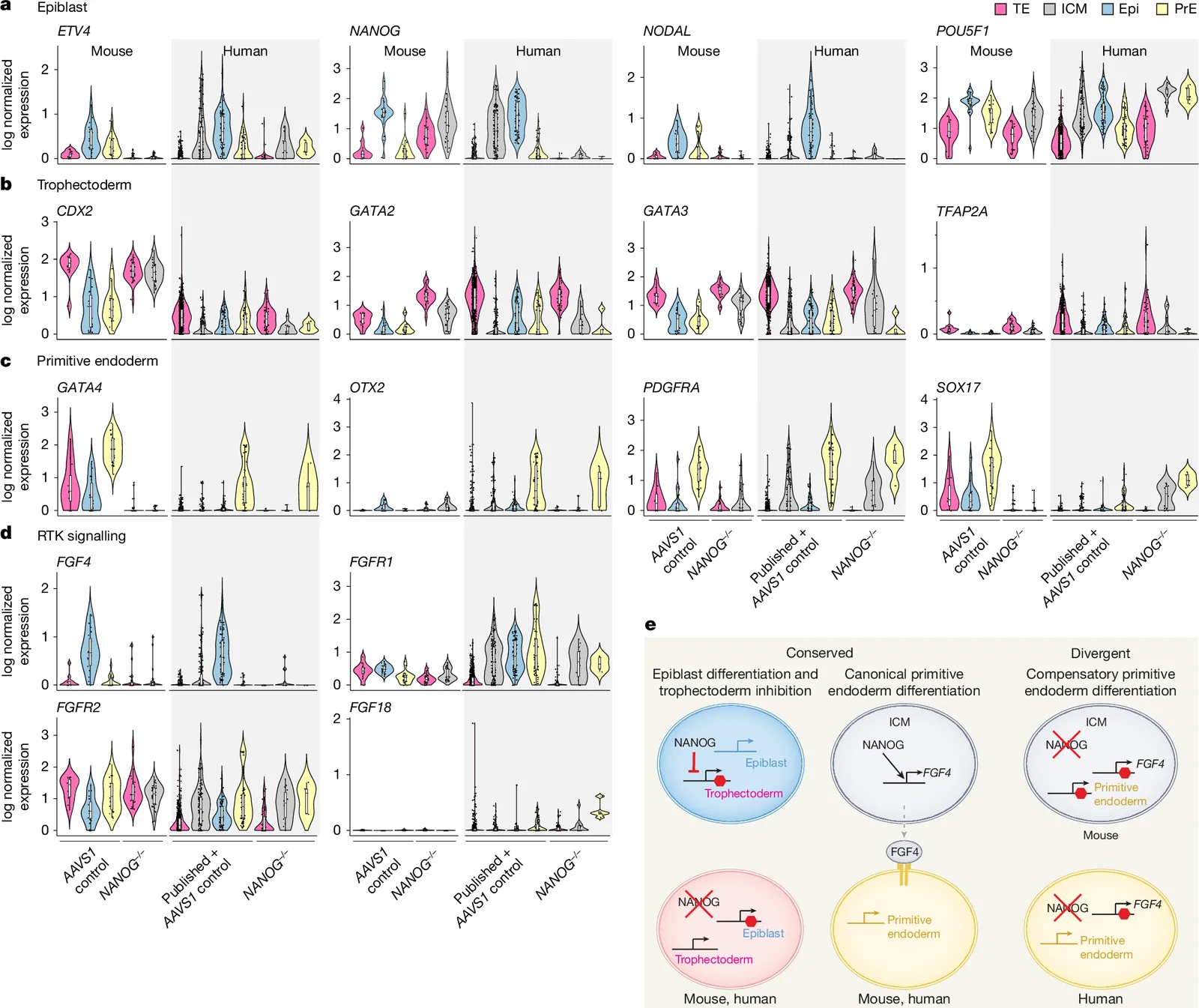
Primitive endoderm differentiation occurs despite *NANOG* mutation in human, but not mouse, embryos. **a**–**d**, Comparison of scRNA-seq of human and mouse *NANOG* (or *Nanog*)-edited embryos versus controls. log-normalized expression of selected genes associated with epiblast (**a**), trophectoderm (**b**) and primitive endoderm (**c**) lineages, and RTK signalling (**d**). *n* = 930 previously published and *AAVS1*-edited control cells, *n* = 72 *NANOG*-edited cells, *n* = 57 *Aavs1*-edited cells, *n* = 58 *Nanog*-edited cells. Box plots represent the interquartile range (IQR), with the median shown as a central line; whiskers (white box) extending to lowest and highest values within 1.5× IQR. Individual dots represent expression in a given cell. **e**, Schematic summary of the role of NANOG in human versus mouse preimplantation development. The loss of NANOG in human and mouse embryos leads to impaired epiblast specification and upregulation of trophectoderm-associated transcripts in both species. FGF4 expression, which has been shown to be required for primitive endoderm specification, is disrupted in both species. Unlike mouse, human *NANOG*-base edited embryos can still differentiate primitive endoderm cells due to an undetermined compensatory mechanism. Source data

To further understand the transcriptome-wide consequences of the loss of *NANOG*, we focused on the embryos in which trophectoderm biopsies suggested a homozygous *NANOG* mutant genotype compared with *AAVS1* and reference control embryos. Global gene expression analysis revealed that compared with control cells, *NANOG*-edited embryos 4, 7 and 8 exhibited significantly reduced expression of epiblast-associated markers, including *NANOG*, *LEFTY1*, *LEFTY2* and *FGF4* (Figs. 3e,f and 4a and Supplementary Table 2). We found reduced expression of *SOX2*, *ETV5*, *PRDM14*, *VENTX* and *NODAL* transcripts in the *NANOG*-edited ICM cells compared with controls, although the difference was not statistically significant (Figs. 3e and 4a and Supplementary Table 2). This suggests that epiblast specification is disrupted in the absence of *NANOG*.

## Primitive endoderm in *NANOG* mutant human but not mouse

**Extended Data Fig. 6:**
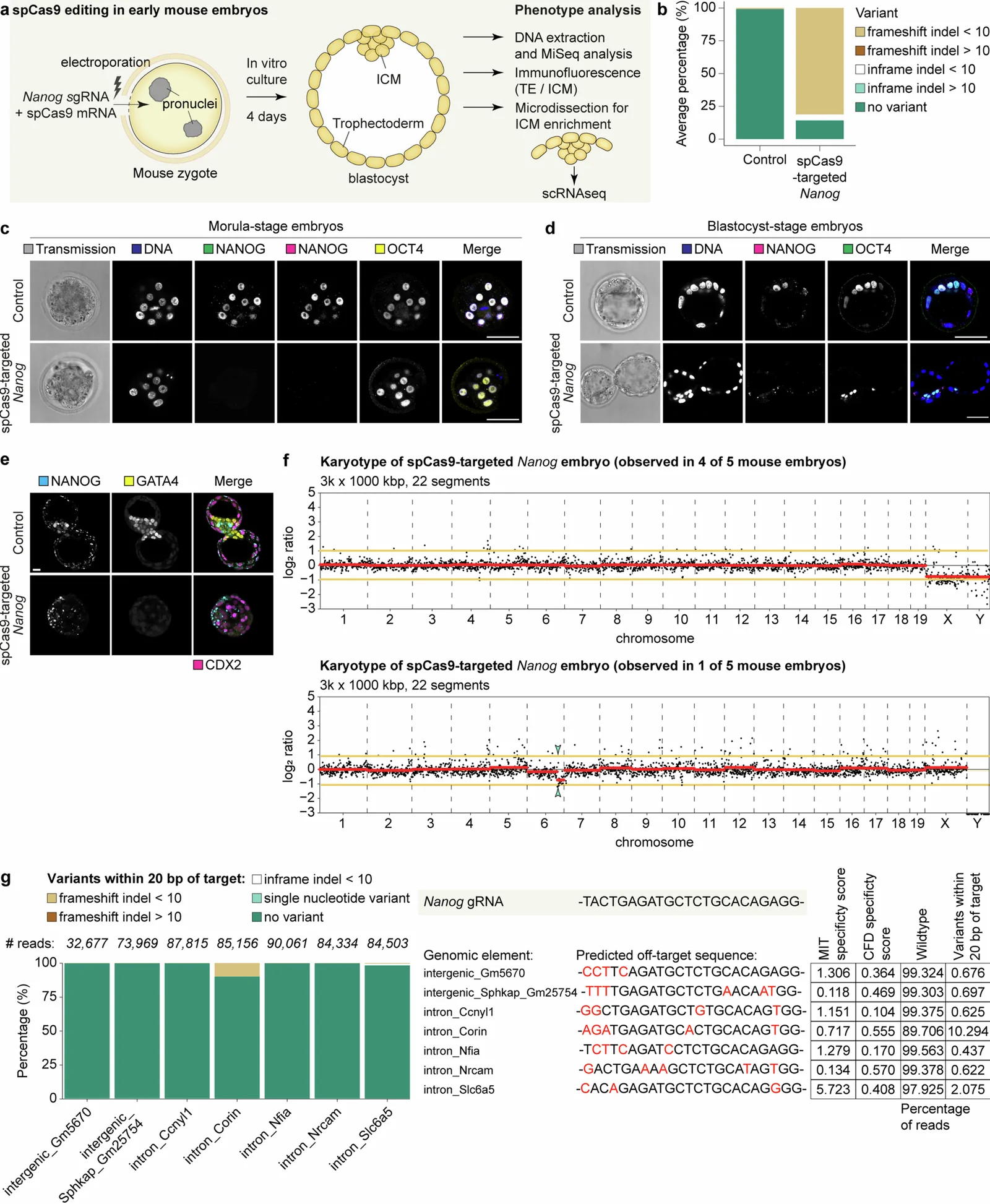
Editing of *Nanog* in mouse zygotes using CRISPR/spCas9 editing. **a**, Schematic of the targeting strategy in mouse zygotes. Zygotes were electroporated with *Nanog* sgRNA and spCas9 ribonucleoprotein complexes. Embryos were culture until 4.5 days post fertilization after which 1) DNA extracted and *Nanog* on-target sequencing was performed; 2) immunofluorescence analysis was performed on whole embryos; 3) microdissection to enrich for the polar region was performed followed by scRNAseq. **b**, Stacked bar charts showing *Nanog* on-target amplicon MiSeq analysis of pooled blastocysts from targeting experiments (n = 3 biological replicates). **c**, Immunofluorescence analysis of two independent Nanog antibodies along with Oct4 and DAPI nuclear staining in control and *Nanog*-edited morula stage embryos (n = 15 *Nanog*-edited and n = 3 control embryos). Scale bar, 50 µm. **d**, Immunofluorescence analysis of DAPI, Nanog and Oct4 nuclear staining in control and *Nanog*-edited blastocyst stage embryos (n = 15 *Nanog*-edited and n = 3 control embryos). Scale bar, 50 µm. **e**, Immunofluorescence analysis of Cdx2, Nanog and Gata4 nuclear staining in control and *Nanog*-edited blastocyst stage embryos (n = 22 *Nanog*-edited and n = 9 control embryos across n = 3 independent replicates). Scale bar, 20 µm. **f**, Low pass whole genome sequencing analysis of CRISPR-spCas9 *Nanog*-edited blastocyst, showing aneuploidy at the *Nanog* locus (blue arrowheads, n = 1/5 blastocysts) and example of karyotypically normal blastocyst (n = 4/5 blastocysts). **g**, Bar chart (left) and quantified data (right) showing amplicon sequencing analysis of 7 in silico predicted *Nanog* gRNA off targets (n = 3 *Nanog*-edited mouse embryos). MIT and CFD specificity score is shown. Source data

**Extended Data Fig. 7:**
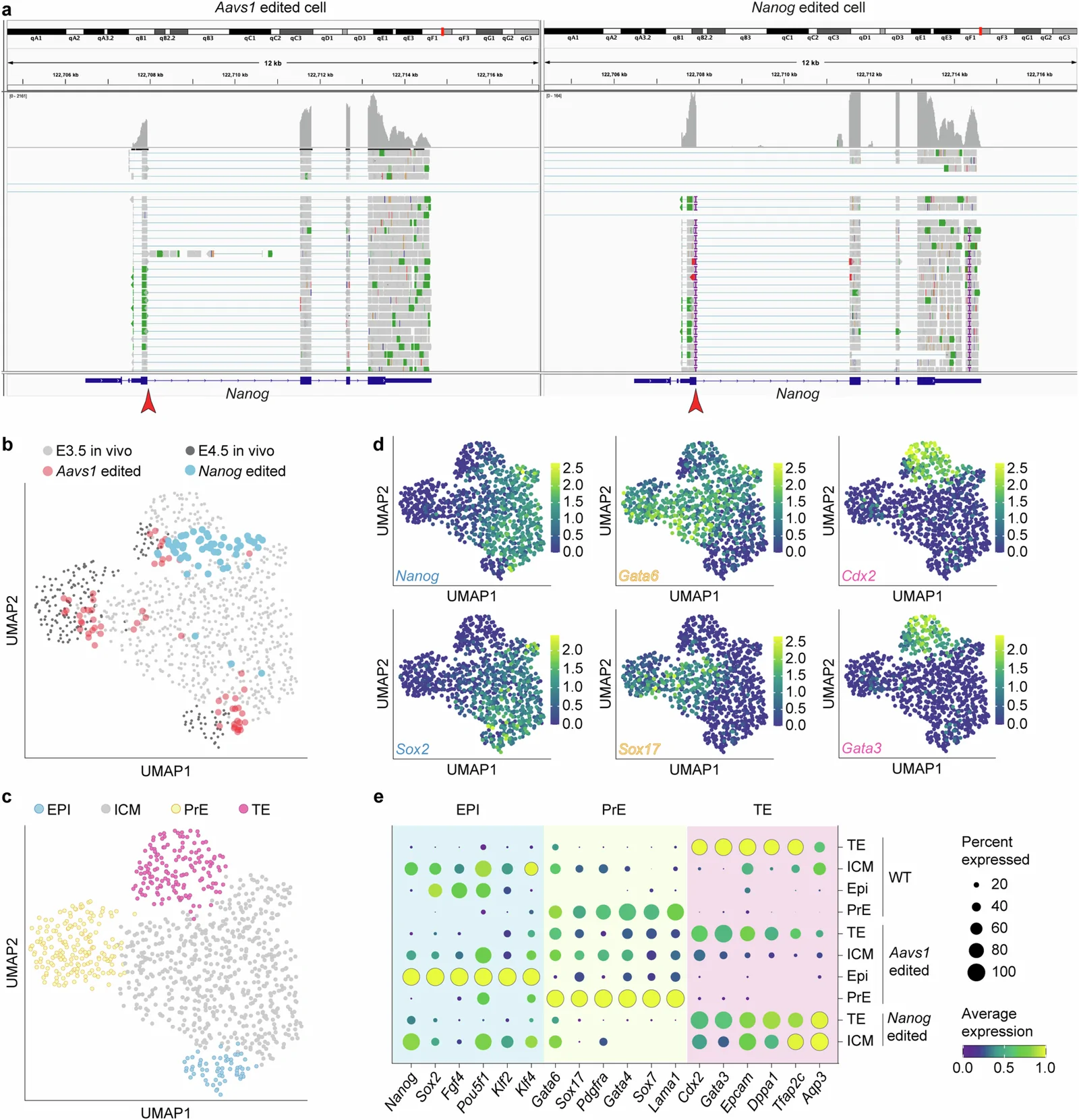
*Nanog*-edited mouse embryos upregulate trophectoderm markers and fail to differentiate epiblast and primitive endoderm cells. **a**, IGV genome browser images showing representative images of cDNA alignments at the *Nanog* locus in cells from *Aavs1-* and *Nanog*-edited blastocysts. Red arrowhead is the site of *Nanog* gRNA. The red arrowhead and purple indicate a 1 base-pair insertion. **b**, UMAP dimensionality reduction plot of scRNAseq data from *Aavs1-* and *Nanog-*edited mouse embryos collected in this study compared to a previously published in vivo reference control dataset from embryonic data (E) 3.5 and 4.5 blastocyst stage embryos^40^ (n = 57 *Aavs1*-edited control cells, n =58 *Nanog*-edited cells, collected from n = 5 mouse embryos per condition; n = 1006 from previously published dataset^40^). **c**, UMAP coloured by assigned lineage based on marker expression in **d** and the Leiden algorithm in Scanpy’s implementation to cluster cells into subgroups using parameter resolution=0.7. **d**, UMAP feature plots showing relative expression of molecular markers enriched in lineages: epiblast (*Nanog, Sox2*), primitive endoderm (*Gata6, Sox17*), inner cell mass (*Gata6*, *Nanog*, *Sox2*) and trophectoderm (*Cdx2, Gata3*). **e**, Dotplot analysis of molecular marker expression associated with the epiblast (EPI), primitive endoderm (PrE), inner cell mass (ICM) or trophectoderm (TE). The percentage of cells and average expression are shown.

**Extended Data Fig. 8:**
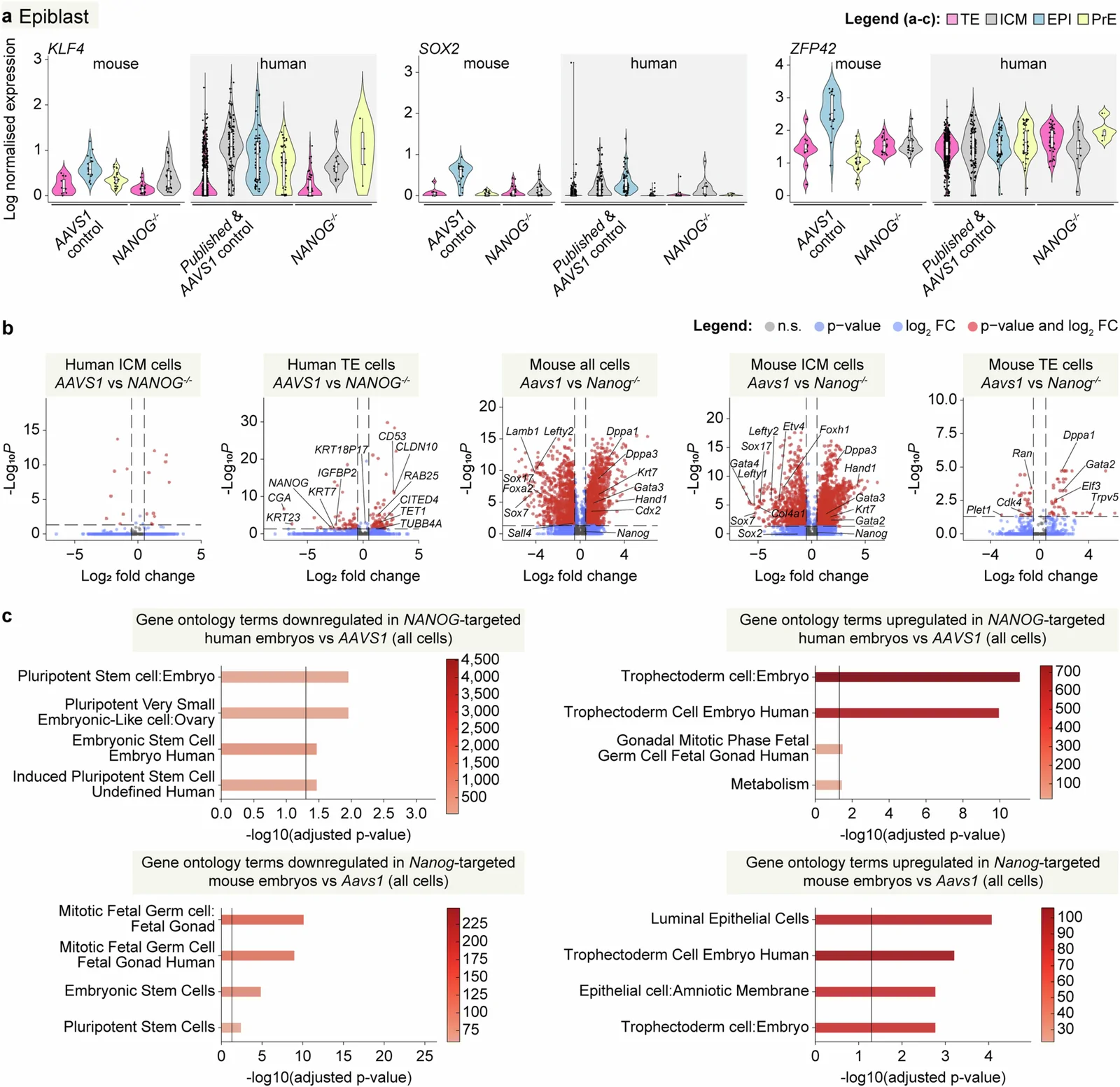
Differences and similarities in the phenotype of *NANOG*/*Nanog*-edited human versus mouse embryos. Comparison of human versus mouse *NANOG*/*Nanog*-edited embryos versus control scRNAseq datasets. Log-normalized scRNAseq expression of selected genes associated with the epiblast. Plots represent the interquartile (IQR range), with the median shown as a central line; whiskers (white box) extend to lowest or highest value within 1.5*IQR; values. Individual points represent expression in a given cell (n = 930 published and *AAVS1*-edited control cells, n = 72 *NANOG*-edited cells, n = 57 *Aavs1*-edited cells, n = 58 *Nanog*-edited cells). **b**, Volcano plots of differential gene expression between *AAVS1/Aavs1*- and *NANOG/Nanog*-edited embryo cell populations in human or mouse embryos. Comparisons of cells clustered in inner cell mass (ICM) lineages only, trophectoderm (TE) cells only, or cells of all lineages are shown. MAST test was used with adjusted p-value < 0.05 and log2FC > 1.0. Adjusted p-values were calculated using the two-sided Bonferroni correction method to control for multiple tests. **c**, Gene ontology analysis of upregulated and downregulated terms in *NANOG/Nanog*-edited versus *AAVS1/Aavs1*-edited human or mouse embryos. Line depicts adjusted p-value of 0.05. Genes were selected from differential expression analysis with adjusted p-value < 0.05. Two-sided Kolmogorov-Smirnov test, adjusted p-values were calculated using the two-sided Benjamini–Hochberg method to control for FDR across multiple tests.

**Extended Data Fig. 9:**
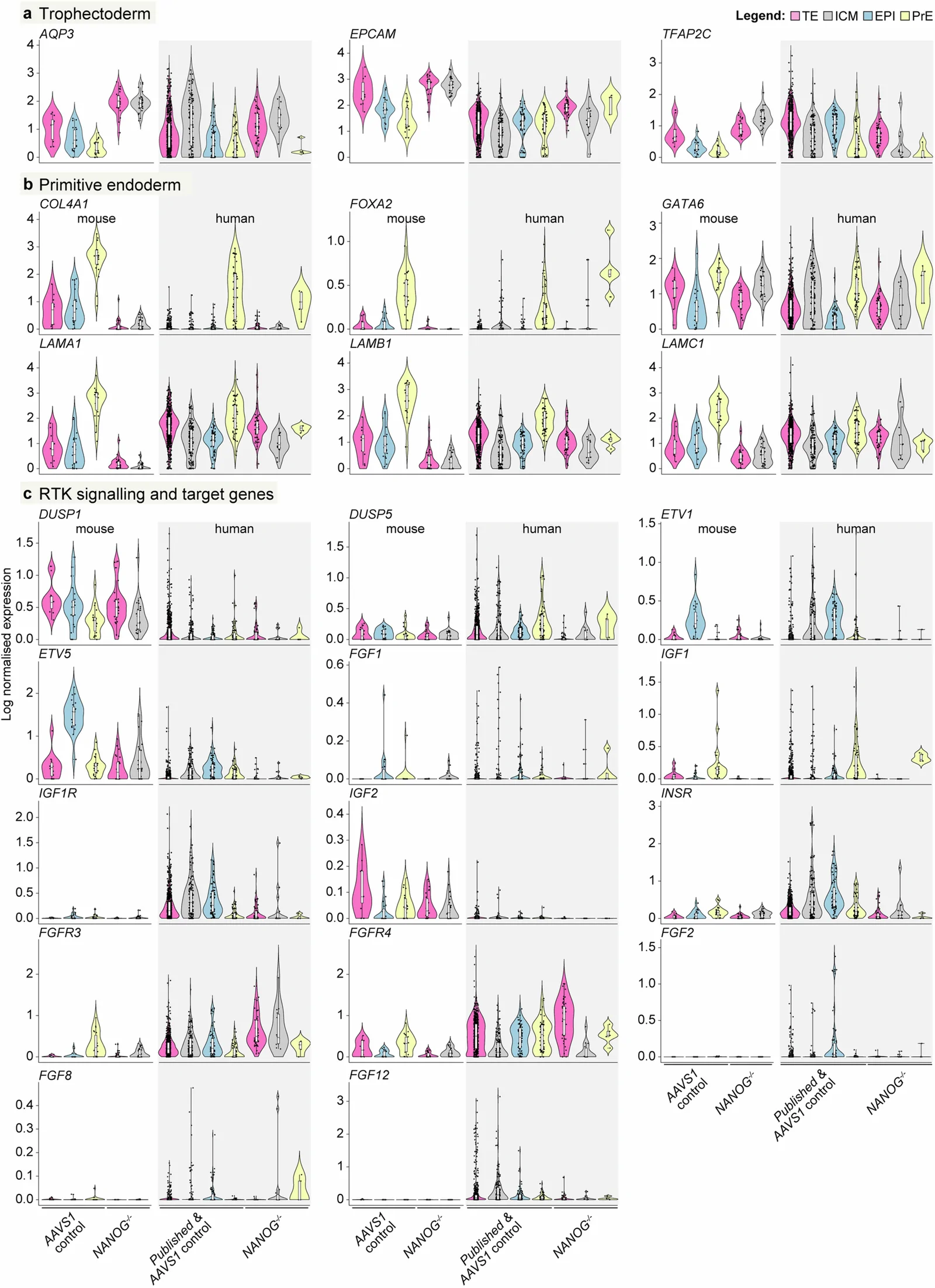
Differences and similarities in extraembryonic and RTK signalling expression in *AAVS1*- versus *NANOG*-edited mouse and human embryos. Comparison of human versus mouse *NANOG*/*Nanog*-edited embryos versus controls. Log-normalized scRNAseq expression of selected genes associated with trophectoderm (**a**), primitive endoderm **(b)** and receptor tyrosine kinase (RTK) signalling and target genes (**c**) (n = 930 published and *AAVS1*-edited control cells, n = 72 *NANOG*-edited cells, n = 57 *Aavs1*-edited cells, n = 58 *Nanog*-edited cells). Plots represent the interquartile (IQR range), with the median shown as a central line; whiskers (white box) extend to lowest or highest value within 1.5*IQR; values. Individual points represent expression in a given cell. Source data

To compare the role of NANOG in mouse and human, we utilized a spCas9 editing strategy in mouse zygotes to target the orthologous 3′ region of *Nanog* exon 1 (Extended Data Fig. 6a). We validated high on-target editing efficiency (Extended Data Fig. 6b), resulting in loss of NANOG and GATA4 protein expression in mouse embryos (Extended Data Figs. 6c–e). Although 4 out of 5 mouse blastocysts that we evaluated following spCas9 genome editing had a normal karyotype, 1 out of 5 was aneuploid at the on-target chromosome (Extended Data Fig. 6f), consistent with the approximately 20% aneuploidy that we and others previously reported following the use of spCas9 enzyme in embryos^1^. Off-target loci predicted in silico included intergenic and intronic regions (Extended Data Fig. 6g). We amplified seven in silico predicted off-target sites using PCR of pooled samples of three *Nanog* genome edited mouse embryos and performed next generation deep sequencing (Extended Data Fig. 6g). Four out of 7 off-target predicted sites lacked observed insertion deletion mutations in mouse *Nanog*-edited embryos, and 2 out of 7 sites had 10% and 2% reads with off-target editing (Extended Data Fig. 6g). We performed scRNA-seq on *Aavs1-* versus *Nanog*-edited mouse blastocysts and enriched for ICM cells in the polar region by laser microdissection (Extended Data Fig. 6a). RNA-seq reads mapping to the *Nanog* locus confirmed successfully edited cells (Extended Data Fig. 7a). Following integration with previously published scRNA-seq of mouse embryos from embryonic day 3.5 to 4.5 (ref. ^40^) (Extended Data Fig. 7b,c), we observed distinct clustering of *Nanog*-edited cells compared with *Aavs1-*edited controls, with divergent lineage marker expression (Extended Data Fig. 7d,e). Notably, *Nanog*-edited mouse embryos did not establish discrete epiblast or primitive endoderm cells, consistent with previous studies^6,7,35,36^. Instead, they clustered with ICM and trophectoderm cells (Extended Data Fig. 7b–d). The *Nanog*-edited ICM-like cells expressed *Nanog*, *Pou5f1*, *Tbx3,* *Zfp42*, *Klf2* and *Klf4*, but downregulated markers such as *Etv4*, *Nodal*, *Lefty1* and *Lefty2* (Fig. 4a and Extended Data Figs. 7e and 8a and Supplementary Table 3). Expression of *Sox2* and *Fgf4* transcripts was reduced in the *Nanog*-edited ICM cells compared with controls, although this did not reach significance (Extended Data Figs. 7e and 8a and Supplementary Table 3). Moreover, the *Nanog*-edited ICM-like cells significantly upregulated trophectoderm-associated markers *Gata2*, *Gata3*, *Krt7*, *Cdx2*, *Epcam*, *Tfap2c*, *Hand1* and *Aqp3*, and gene ontology enrichment further indicated that lineage specification was compromised (Fig. 4b and Extended Data Figs. 8b,c and 9a and Supplementary Table 3). The inability of both mouse and human embryos to specify the pluripotent epiblast indicates conservation of this phenotype between these species (Figs. 3e and 4a, Extended Data Figs. 7e and 8a–c and Supplementary Table 4).

The downregulation of *Fgf4* expression in the mouse is likely to account for the failure of primitive endoderm differentiation (Fig. 4c,d and Extended Data Fig. 7e), because FGF4 is a key NANOG-regulated ligand that is essential for primitive endoderm maturation^36,41,42^. In contrast to the mouse, we note that human *NANOG*-edited cells differentiated to the primitive endoderm, despite the absence of *FGF4* transcripts (Figs. 3c–f and 4c,d). This is surprising, given the role of receptor tyrosine kinase (RTK) signalling via the FGF–MEK–ERK pathway in human primitive endoderm specification^43,44^. We confirmed that *NANOG*-edited cells express a number of key primitive endoderm-associated markers such as *GATA6*, *GATA4*, *PDGFRA*, *SOX17*, *FOXA2* and *OTX2* and basement-membrane associated factors such as *LAMA1*, *LAMB1*, *LAMC1* and *COL4A1* (Fig. 4c and Extended Data Fig. 9b).

Given the lack of *FGF4* transcription in *NANOG*-edited cells, we investigated the expression of genes encoding alternative ligands and any receptors associated with RTK signalling (Fig. 4d and Extended Data Figs. 9c). We identified expression of genes encoding FGF receptors (*FGFR1*, *FGFR2*^42^, *FGFR3* and *FGFR4*), insulin receptor genes (*INSR* and *IGFR1*), FGF ligands (*FGF1*, *FGF12* and *FGF18*) and insulin growth factor ligands (*IGF1*). This suggests either that alternative ligands bind to FGF receptors or that RTK signalling is activated by alternative ligands and receptors that compensate for the loss of *FGF4*. We previously showed crosstalk between FGF and IGF signalling in stem cell proliferation and that treatment with exogenous IGF1 led to increased numbers of ICM cells with embryos in a diapause-like state^45,46^; it would therefore be interesting to determine whether FGF18, IGF1 or alternative RTK signalling components can compensate for the loss of *NANOG* and whether they may have a role in primitive endoderm specification in a non-perturbed context.

Overall, we show that adenine base editing is a precise and effective strategy for functional genetic interrogation in early human embryos, overcoming some of the genotoxic limitations of conventional CRISPR–spCas9-mediated genome editing. Here we apply adenine base editing to disrupt *NANOG*, revealing a requirement for this gene in human pluripotency and epiblast specification (Figs. 3c–f and 4e). In contrast to mouse, we observed that *NANOG*-edited human embryos retained primitive endoderm cells despite the absence of FGF4 (Fig. 4e), and our data suggest a number of plausible RTK signalling components that would be interesting to investigate in the future. The inability of *Nanog*-null mutant mouse embryos to give rise to epiblast or mature primitive endoderm cells, and the expression of trophectoderm-associated transcripts in the ICM, is consistent with previous studies^6,7,35,36^. This suggests divergence in gene function between the human and mouse, consistent with our previous studies indicating differences in OCT4 function^16^.

Whereas base editing of exon–intron splice sites has been reported in growth hormone receptor in porcine cells, major histocompatibility complex in immune cells and to target fluorescent proteins^15,47,48^, to our knowledge, this is the first demonstration of base editing used to disrupt a conserved splice donor site to induce a splicing defect and generate a loss-of-function mutation in a developmental regulator across any cell type. This approach will be broadly applicable to other developmental regulators and a substantial improvement over other CRISPR–Cas9 approaches. Given that G•C>A•T substitutions underlie nearly half of all known pathogenic variants^5^, base editing approaches have been proposed as potential tools for disease modelling and therapeutic correction. Although the present study was not designed to assess clinical applicability of base editing in the human germline, our findings do demonstrate reduced aneuploidy following genome editing compared with previous Cas9-mediated approaches^1–3^. Although these observations suggest potential for base editing to investigate gene function in early-stage human embryos, substantial basic research is necessary before potential therapeutic correction. This includes extensive evaluation of safety, such as further assessment of off-target activity, genotoxicity and developmental potential in a larger cohort and across more genomic loci in basic research before consideration for clinical use in heritable genome editing. Base editor variants such as ABE8e-V106W^5^ and CBE6b-V106W^49^ show improved safety through reduced DNA and RNA off-target editing in cell and animal studies, but their performance in human embryos is not yet characterized. Crucially, clinical translation of genome editing in human embryos must not proceed unless there is broad societal debate and support^50–52^. Caution, underpinned by rigorous ethical inquiry and oversight together with extensive public discourse, are needed. Together, these findings define a previously unappreciated requirement for *NANOG* in human development and provide a generalizable strategy for precise and minimally disruptive genome engineering in early embryos.

## Methods

### Ethics statement

The human embryos and gametes used in this work were donated to the University of Cambridge and Francis Crick Institute following assisted reproductive treatment at UK clinics. Oversight of this research and licencing approvals were conducted by the UK Human Fertilisation and Embryology Authority (HFEA) under research license numbers R0162 and R0152 at centres 0397 and 0401. License approval involved independent review by the HFEA License and Executive Committees, as well as the UK Health Research Authority’s Research Ethics Committee IRAS projects 308099, 252286 and 272218. All human embryo research conducted was compliant with the HFEA Code of Practice, and regular audits and inspections were performed.

Informed consent was obtained from all donors by suitable qualified clinic staff not involved in the research or directly involved in treatment. Human oocytes, gametes and embryos donated as supernummary to assisted conception treatment requirements were not financially compensated. Alternatively, human oocytes were donated as part of an ‘egg sharing’ programme or by non-patient donors. A subsidy of £1,500 or an additional cycle of treatment was provided to donations made through the egg sharing programme, while non-patient donors received a £500 financial compensation per donation cycle. Before giving consent, donors were provided with all of the necessary information about the research project, including details of the biochemical and genetic studies, such as genome editing, that would be performed. Donated embryos and gametes were cryopreserved and transferred to the University of Cambridge and Francis Crick Institute, where they were thawed and used in the research project.

### Egg retrieval and fertilization

Oocytes were retrieved via ultrasound-guided follicle aspiration, and the surrounding cumulus cells were removed using hyaluronidase (HYASE; Vitrolife). Metaphase II (MII) oocytes were identified by the presence of the first polar body and subsequently fertilized through ICSI using sperm specifically donated for this study. The experiments were not randomized, and investigators were not blinded to group allocation during the procedures or outcome assessments.

### Human embryo thaw and culture

Human MII oocytes were thawed using the RapidWarm Oocyte Kit (VitroLife, 10122). Human oocytes or embryos were alternatively thawed using the Oocyte/Embryo Thawing Media kit (Kitazato, VT602). All kits were used per the manufacturer’s instructions.

Dishes were prepared with G-IVF (VitroLife, 10134) for oocytes or Gx-TL (VitroLife, 10172) for zygotes. Oocytes were cultured at 37 °C, 6% CO_2_ and 5% O2 prior to ICSI. Following ICSI the fertilized eggs were cultured at 37 °C and 6% CO_2_ in EmbryoSlide+ culture dishes (VitroLife, 16450) inside an EmbryoScope+ time-lapse incubator (VitroLife). Culture medium was overlaid with mineral oil (Cooper Surgical, ART-4008-5P). Dishes were prepared following the manufacturer’s instructions.

### Human sperm thawing

Frozen semen samples (~1 ml) were thawed at 37 °C in atmospheric gas incubator or water bath. A density gradient was prepared using 80% and 40% Puresperm solutions (Puresperm and GMOPS, Vitrolife). The gradient was assembled in 15 ml Falcon tubes, and the semen was layered on top. Samples were centrifuged at 300*g* for 5 min.

After centrifugation, the supernatant was removed and the sperm pellet was washed in G-MOPS (Vitrolife), followed by a second centrifugation under the same conditions. The final pellet was resuspended in 100 µl G-MOPS. Sperm quality was briefly assessed prior to ICSI by observing a small aliquot under the microinjection microscope.

### Human ICSI

An injection plate was prepared with multiple wash droplets of G-MOPS and G-IVF. One droplet of 10% polyvinylpyrrolidone (PVP) (Cooper Surgical, ART-4005-AA) was used to wash the ICSI injection needle to prevent sticking and a 10 μl streak of 10% PVP was used to reduce sperm motility. Mineral oil was overlaid and the plate was warmed to 37 °C before use. Injection (Cooper Surgical, MIC-SI-30) and holding pipettes (Cooper Surgical, MPH-MED-30) were used for ICSI.

One microlitre of the sperm preparation was pipetted into the bottom of the PVP streak and sperm that swam to the top were selected. Sperm were immobilized by striking the tail and were then drawn into the injection pipette. To perform adenine base editing coincident with ICSI, a solution of 50 ng μl^−1^ sgRNA and 100 ng μl^−1^ ABE8e mRNA or 100 ng μl^−1^ ABE8e protein was prepared in 1:10 Buffer TE (Invitrogen, 12090015) diluted in nuclease free water. Ribonucleoprotein (RNP) complexes were produced by incubating the mixture at 37 °C for 15 min. The solution was kept on ice until needed. A 10 μl droplet of the solution was prepared on the injection plate. The injection pipette containing sperm was expelled until the sperm was at the opening of the ICSI needle, then the sgRNA/ABE8e mRNA solution was aspirated to approximately two times the length of a sperm from the tip of the head to the end of the tail.

Oocytes were placed into the G-MOPS droplets. Oocytes were held with the polar body at the 12 or 6 o’clock position and injection needle approaching at the 3 o’clock position. The solution inside the injection pipette was expelled until the sperm had approximately one sperm length of medium ahead of it in the pipette, halving the volume of the aspirated base editing solution. The injection needle was inserted into the oolemma at a position approximately two-thirds the length of the oocyte. The ooplasm was gently aspirated until the oolemma visibly broke. The sperm was then rapidly and gently expelled into the oocyte. The pipette was removed and the holding pipette released the oocyte to allow membrane closure. Eggs were fertilized in groups of fewer than three and washed in Gx-TL before returning to an incubator for culture in an EmbryoScope incubator at 37 °C and 5% CO_2_ for culture until the blastocyst stage, 6 days post-fertilization.

### Human embryo single-cell collection

For single-cell collection, lysis buffer was prepared using the SMART-Seq v4 Ultra Low Input kit (Takara Bio, 634891) per manufacturer’s instructions. Laser microdissection (RI Saturn 5 Active Laser, Cooper Surgical) was used to enrich for polar region, comprising epiblast, primitive endoderm and overlying polar trophectoderm. The mural trophectoderm was further microdissected to collect replicate biopsies for genotyping and karyotyping.

Dishes were prepared with 500 μl TryplE Express (Thermo Fisher, 12604013) and warmed to 37 °C. The polar region was washed twice and deposited into the TryplE Express solution. Incubation was at 37 °C for 8–10 min.

Polar regions were then washed in three droplets of G-PGD medium (VitroLife, 10074). Glass capillaries (World Precision Instruments, TW100F-6) had been previously prepared using a Pipette Puller (Sutter Instruments, Model p-1000). Capillaries were broken with bore sizes ranging from 40 μm to 100 μm.

Using the glass capillary, polar regions were mouth pipetted up and down until single cells became detached. Cells were picked immediately once detached and deposited into lysis buffer. Cells were spun down and snap frozen on dry ice in Lysis Buffer from the SMART-Seq v4 Ultra Low Input kit (Takara Bio, 634891).

### ABE8e expression and purification

ABE8e protein was prepared following previously established protocol^53^. The pABE8e plasmid was transformed into *Escherichia coli* BL21 Star (DE3) competent cells (Thermo Fisher Scientific). Protein expression was induced in a 1 l culture grown in Terrific Broth supplemented with 25 µg ml^− 1^ kanamycin at 37 °C to an OD_600_ of 1.3 to 1.5. The culture was cold-shocked in ice water for 1 h and protein expression was induced by addition of l-rhamnose (Sigma) to a final concentration of 0.8% (wt/vol). The induced culture was incubated at 18 °C with shaking for 24 h. Cells were collected by centrifugation at 4,000*g* for 20 min, flash-frozen in liquid nitrogen, and stored at −80 °C.

Frozen cell pellet from 1 l of culture was resuspended in 30 ml of bacterial lysis buffer (1 M NaCl, 100 mM Tris-HCl pH 8.0, 1.5 mM TCEP, and 20% (vol/vol) glycerol supplemented with two EDTA-free cOmplete protease inhibitor tablets (Roche) per 50 ml buffer). Cells were lysed by sonication and the lysate was clarified by centrifugation at 20,000*g* for 20 min at 4 °C. The clarified supernatant was supplemented with imidazole to a final concentration of 30 mM using a 3 M stock solution at pH 8.0. Ni-NTA resin (1.5 ml; Qiagen) was added to the lysate and incubated with gentle rotation for 1 h at 4 °C to allow protein binding. The resin-lysate mixture was transferred to a disposable gravity-flow chromatography column, washed twice with 20 column volumes of high-salt buffer (1 M NaCl, 100 mM Tris-HCl pH 8.0, 1.5 mM TCEP, and 20% (vol/vol) glycerol), and bound protein was eluted using four column volumes of elution buffer (0.45 M NaCl, 100 mM Tris-HCl pH 8.0, 1.5 mM TCEP, 20% (vol/vol) glycerol, and 300 mM imidazole). The eluate was diluted tenfold with low-salt buffer (100 mM Tris-HCl pH 8.0, 1.5 mM TCEP, and 20% (vol/vol) glycerol) and immediately loaded onto a 5 ml HiTrap SP HP cation exchange column (Cytiva). The column was washed with three column volumes of low-salt buffer. Proteins were eluted using a linear gradient from 100% low-salt buffer to 80% high-salt buffer over a total volume of 50 ml at a flow rate of 5 ml min^−1^. Fractions containing only the ABE8e protein (185 kDa) were pooled, sterile-filtered using a low protein-binding 0.22-µm PES membrane filter (Merck) and concentrated using 100 kDa molecular weight cutoff centrifugal filters (Merck). The protein concentration was measured by NanoDrop One spectrophotometer (Thermo Fisher Scientific) and the final concentration was 4.3 mg ml^−1^. Protein aliquots were flash-frozen in liquid nitrogen and stored at −80 °C.

### Mouse embryo collection and culture

At the Francis Crick Institute, F_1_ (C57BL6 × CBA) 4- to 8-week-old females were super ovulated and mated to F_1_ (C57BL6 × CBA) 2- to 12-month-old male mice. The study was approved by the Animal Ethics Committee and by the UK Home Office licence number 70/8560, following all relevant institutional and national guidelines and regulations. At the University of Cambridge Physiology, Development and Neuroscience Department, CD1 10- to 12-week-old females were naturally mated to CD1 6- to 12-month-old male mice. All mice were kept at 21 °C, 50% humidity and subject to a standard light cycle of 12 h. Embryos were collected and cultured as described^54^. Zygotes were collected in EmbryoMAX FHM Mouse Embryo medium (Millipore, MR-122-D) and cumulus cells were removed with hyaluronidase (Sigma, H4272). Mouse embryos were placed in pre-equilibrated EmbryoMAX Advanced KSOM (Millipore, MR-101-D) overlaid with mineral oil (Origio; ART-4008-5P) at 37 °C and 5% CO_2_.

### Mouse embryo electroporation

In brief, mouse zygotes were transferred to droplets of FHM medium overlaid with mineral oil then underwent zona drilling to generate a single hole using a RI Saturn 5 Active Laser (Cooper Surgical). Zygotes were then washed in droplets of OptiMEM (Gibco, 31985-062) before being loaded into the electroporation plate. Zygotes were electroporated in OptiMEM on a NEPA21 platinum electrode plate (NepaGene, CUY501P1-1.5) using a NEPA21 Super Electroporator. Cas9 enzyme (LabOmics, Cas9-TOO-250) was pre-incubated with sgRNA for 15 min at 37 °C to form RNPs. The program used was 20 V, pulse length 25 ms, pulse interval 50 ms, 2× pulses. Embryos were then washed through FHM medium then washed and cultured in Advanced KSOM until the blastocyst stage.

### hESC culture

H9 hESCs (WiCell) were cultured on Matrigel-coated 6-well dishes (BD Biosciences, 356231). Cells were split at a 1:10–1:20 ratio every 3–4 days depending on confluence using ReLeSR (STEMCELL Technologies, 05872). Cells were fed daily with mTeSR1 (STEMCELL Technologies, 85831), supplemented with 1% penicillin/streptomycin (Gibco, 15140122). Cell pellets were resuspended in mTeSR1 supplemented with 1× CloneR2 (STEMCELL Technologies, 100-0691) prior to plating. hESCs were screened for mycoplasma presence on a monthly basis. hESCs were assessed for cell line identification and karyotype on a quarterly basis.

### Lipofection of primed hESCs

Seventy per cent confluent hESCs were dissociated to single-cell density using Accumax (Thermofisher Scientific, 00-4666-56). Cells were counted using a haemocytometer. Cells were then plated at a density of 3 × 10^5^ cells per well of a 6-well plate. Twenty-four hours later, cells were lipofected using Lipofectamine Stem Reagent (Thermo Fisher, STEM00003), following manufacturer’s instructions. Plasmid (1.5 µg) was used per well of a 6-well dish. The Francis Crick Institute Viral Vector Core produced an ABE8e (Addgene plasmid #138489) plasmid, by substituting the Cas9 region from pX459 (Addgene plasmid #62988) for ABE8e. The resultant plasmid contained an sgRNA sequence cloning site, ABE8e and puromycin resistance gene. After 24 h, medium was replaced with mTeSR1 supplemented with 0.5 µg ml^−1^ puromycin for 48 h. Cells were fed with mTeSR1 until collection after 3–7 days. The pX459 backbone was a gift from F. Zhang (Addgene plasmid #62988).

### Single-cell cloning of *NANOG*-edited hESCs

H9 hESCs were first lipofected as described above. After 2–3 days following puromycin selection, when colonies were of medium size, they were picked manually for cloning. In brief, colonies were scored and picked using a p20 pipette, then transferred into a 96-well plate containing Accumax. Cells were then incubated for 10 min at 37 °C. Afterwards, cells were gently pipetted to a single-cell density then deposited into Matrigel-coated 12-well plates containing mTeSR1 with 1× CloneR2 and cultured as described above.

### Design and ligation of sgRNAs

sgRNAs were designed using a combination of the in silico design tools CRISPOR, Benchling and Synthego Design Tool^55^. Guides were selected primarily based on having lowest predicted off-target binding to other regions of the genome. Other key considerations for sgRNAs for use in diverse human genetic backgrounds have been previously described^23^. For design of base editing sgRNAs, the position of the target sequence at an exon–intron boundary was prioritized. sgRNAs were selected which had the target A base of the exon–intron boundary motif at positions 13–17 distal from the PAM sequence. sgRNA sequences were ligated into pX459 or ABE8e-based plasmids as previously described^56^. The *NANOG* and *Nanog* sgRNA sequences used are provided in Fig. 1a, Extended Data Fig. 3a and Supplementary Table 6.

### Generation of ABE8e mRNA

Plasmids containing a T7-ABE8e sequence (Addgene #138489) were linearized using the SpeI-HF restriction enzyme (NEB; R3133S) at 37 °C overnight. Linearized plasmid DNA was cleaned by phenol/chloroform extraction. DNA was concentrated by ethanol precipitation using 3 M sodium acetate.

In vitro transcription was performed using the mMESSAGE mMachine T7 ULTRA kit (Invitrogen, AM1345) per the manufacturer’s instructions, with incubation for 2 h. RNA was cleaned using the QIAGEN RNeasy kit (QIAGEN, 74104) per the manufacturer’s instructions.

### Genomic DNA extraction and assessment of editing efficiency

Collected cells and embryos were processed using QIAGEN DNeasy Blood and Tissue Kit (QIAGEN, 69506) or by low-input whole-genome amplification using the RepliG Single Cell Kit (Qiagen, 150343), respectively. Both kits were used per the manufacturer’s instructions.

On- and off-target genome editing was assessed by PCR amplification of the target region. PCR reactions used the Q5 high-fidelity kit (New England BioLabs, M04925). Primers included the addition of Illumina MiSeq adaptor sequences. The PCR reactions were cleaned using AMPure XP beads (Beckman Coulter, BECLA63881) following the manufacturer’s instruction. Subsequently, target amplicon sequencing was performed either by Sanger sequencing or Illumina MiSeq (paired end reads of 250 base pairs). The sequencing primers used are reported in Supplementary Table 7.

### Human embryo immunofluorescence

Human blastocyst stage embryos were fixed in 4% methanol-free formaldehyde (Thermo Scientific, 28906) in phosphate-buffered saline (PBS, Sigma-Aldrich, D8537) for 20 min at room temperature. Fixed embryos were permeabilized in PBS (Sigma-Aldrich, D8662) containing 0.2% Triton X-100 (Sigma-Aldrich, 93443) for 10 min at room temperature, then blocked in 1% BSA (Fisher BioReagents, BP1605-100) in PBS (Sigma-Aldrich, D8662) overnight at 4 °C. Primary antibodies were diluted in 1% BSA in PBS and incubated overnight at 4 °C. Secondary antibodies were diluted in 1% BSA in PBS and DAPI used for nuclear staining. All primary and secondary antibodies and the dilutions used are listed in Supplementary Tables 8 and 9.

### Confocal imaging and image analysis of human embryos

Confocal imaging was performed in PBS (Sigma-Aldrich, D8662) with 3 mg ml^−1^ polyvinylpyrrolidone (Sigma-Aldrich, P0930) under paraffin oil in a 35 mm dish with a no. 1.5 glass coverslip. Images were acquired with SP8 confocal laser scanning microscope (Leica) using a plan apochromatic 40× 1.10 NA water-immersion objective. Nuclei were detected in the DAPI channel using the Spot Detection function in Imaris (Bitplane). The spot diameter was set to 8 µm. Following manual correction, mean fluorescence intensities were extracted for the GATA3, GATA4 and NANOG channels for each detected nucleus. Cells were classified automatically according to the channel with the highest mean fluorescence intensity. Spots/nuclei with mean fluorescence intensities below 2.5 arbitrary units in all three channels were classified as negative or unassigned, as the signal was considered too low for reliable classification. Images were acquired in 8-bit format, with an intensity range of 0–255.

### Mouse embryo immunofluorescence

Mouse embryos were washed in PBS then placed into 4% paraformaldehyde in PBS for 1 h at 4 °C. Embryos were then washed 3 times in PBS containing 0.1% Triton (PBS-T). To permeabilize, embryos were incubated in PBS-T (0.5%) for 20 min at room temperature. Blocking was performed by placing embryos in PBS-T (0.1%) supplemented with 5% donkey serum and 1.5% bovine serum albumin for 1 h. Primary antibodies were added by diluting in blocking solution and embryos incubated in this solution overnight at 4 °C. The following morning, embryos were washed several times in blocking solution, then incubated in PBS-T (0.1%) containing secondary antibodies for 1 h at room temperature. After washing 3 times in PBS-T, embryos were placed in 1:30 DAPI-Vectashield mounting medium (Vector Labs) in PBS-T (0.1%). All primary and secondary antibodies and the dilutions used are listed in Supplementary Tables 8 and 9.

### hESC immunofluorescence

hESCs were washed with PBS then fixed in 4% paraformaldehyde in PBS for 1 h at 4 ^o^C. hESCs were then washed 3 times with PBS containing 0.1% Triton (PBS-T). For permeabilization, hESCs were placed in PBS-T (0.5%) for 20 min at room temperature. hESCs were blocked in PBS-T (0.1%) supplemented with 10% donkey serum, or 5% donkey serum and 1.5% bovine serum albumin for 1 h. Primary antibodies were added by diluting in blocking solution and cells were incubated in this solution overnight at 4 °C. The following morning, hESCs were washed several times in blocking solution, then incubated in PBS-T (0.1%) containing secondary antibodies for 1 h at room temperature. After washing 3 times in PBS-T, 1:30 DAPI-Vectashield mounting medium (Vector Labs) in PBS-T (0.1%) was added to the hESCs prior to imaging. All primary and secondary antibodies and the dilutions used are listed in Supplementary Tables 8 and 9.

### Immunofluorescence imaging

Mouse embryos and hESCs were imaged on a Leica SP8 inverted microscope (Leica Microsystems). Images were captured with dimensions of either 512 × 512 or 1,024 × 1,024, line averaging was set to 2 and bidirectional *x* was used. For *z*-stacks, section thickness was set at 1 µm or 2 µm. Magnification used 10×, 20× or 40× oil-immersion objectives.

### Bulk RNA-seq

Primed hESC were used for the bulk RNA-seq experiments. hESCs were dissociated using ReLeSR. 1 ml PBS was added and gentle pipetting was performed to remove all cells from the surface of the well. The cell suspension was then centrifuged and the supernatant removed.

RNA was isolated using the Monarch Total RNA Miniprep (NEB, T2010S) according to the manufacturer’s instructions. RNA was then submitted to the Crick’s Genomics Facility for further processing. The facility performed library preparation using the NEBNext Ultra II Directional PolyA mRNA kit (NEB, E7760S). RNA sample quality was measured using a ScreenTape Assay on a 4200 TapeStation (Agilent). Sequencing was performed using a NovaSeq 6000 (Illumina) with average sequencing depth of 25 million reads.

### scRNA-seq

Single cells from mouse and human experiments were collected as described above. Cells were thawed and processed using the SMART-Seq v4 Ultra Low Input kit (Takara Bio, 634891), following manufacturer’s instructions for single-cell processing. Single-cell RNA-seq libraries were prepared using a Nextera XT DNA Library Preparation Kit (Illumina) according to the manufacturer’s protocol. Sequencing was carried out on a NovaSeq 6000 (Illumina) or AVITI (Element Biosciences) machine at a depth of ~12 million reads per cell.

### Karyotype analysis

For low-pass whole-genome sequencing, samples were subject to whole-genome amplification (Qiagen RepliG Single Cell Kit, 150343) followed by low-pass next generation MiSeq sequencing (Illumina) platform to approximately 1× coverage. An analysis pipeline based on the QDNASeq Package was used to generate chromosome copy number profiles. In brief, the genome was subdivided into 1,000-kilobase sections, with the number of reads mapping to a region calculated. A log_2_ ratio of reads mapping to each locus versus the average number of reads mapping to all other loci is calculated to predict gain or loss of a chromosomal region.

### Bioinformatic analysis of sgRNA efficiency

FASTQ alignment was performed using BWA^57^ to map paired end reads to the genome. SAMtools^58^ was then used for processing. Analysis was performed using the CrispRVariants package in R^59^. Further details have been previously described^23^. The following Github scripts were used: https://github.com/jzohren/crispr-miseq. To control for PCR amplification and sequencing errors, Cas9 edited mouse embryo reads had uncategorized (‘Other’) reads and single nucleotide variants of <1% each incidence filtered, and mouse embryo and hESC adenine base editing reads were assessed for editing at the on-target and bystander bases only. Human off-target DNA editing was assessed using CRISPResso2^60^.

### Bioinformatic analysis of scRNA-seq

Cutadapt version 4.2 package was used to remove sequencing adaptors from FASTQ files of scRNA-seq reads, with minimum read length set to 50. FastQ Screen version 0.13.0 and FastQC version 0.11.8 were used for assessing read quality. Alignment was performed using HISAT2 version 2.1.0 for mouse, and STARsolo 2.7.1b for human^61,62^. The mouse genome (*Mus musculus* GRCm38 release-89) or the human genome (*Homo sapiens* GRCh38 release-89) were used. For mouse, BAM files were generated using SAMtools version 1.18. Count matrices were generated using HTseq version 2.0.5^63^. Integration with previously published human embryo samples^37–39^ was performed using Seurat version 5.0.2^64^.

Dimensionality reduction and feature expression was performed using the R package Seurat version 5.0.2. For mouse cells, sample filtering was performed to exclude cells with fewer than 5,000 features, greater than 5% mitochondrial read content, and cells with less than 5 million or more than 20 million total RNA counts. For human cells, sample filtering was performed to exclude cells with fewer than 1,000 features, greater than 20% mitochondrial read content, and cells with fewer than 20,000 total RNA counts. Features expressed in fewer than three cells were also excluded. Cells were clustered using the Seurat FindNeighbors and FindClusters functions. In human embryos, after clustering, UMAP visualization was used to re-assign 14 trophectoderm cells and 4 primitive endoderm cells to local clusters.

Differential gene expression was performed with Seurat using the MAST test. Gene set enrichment analysis was performed using Enrichr^65^.

### Bioinformatic analysis of bulk RNA-seq

FASTQ files of bulk RNA reads were trimmed and aligned and a count matrix was generated using the methods described for scRNA-seq. Differential gene expression was performed using the R package DESeq2 version 1.42.1^66^. Features expressed in fewer than three samples or with fewer than ten counts were removed. False discovery rate was calculated using the Benjamini–Hochberg method. Gene set enrichment analysis was performed using Enrichr^65^.

### RNA off-target editing analysis

For human experiments, three wells of H9 hESCs were lipofected with plasmids containing Cas9 only or plasmids containing *NANOG* gRNA and ABE8e. Cells were collected after 48 h and processed using the Monarch Total RNA Miniprep (NEB, T2010S) according to the manufacturer’s instructions. RNA off-target editing was performed using a previously established pipeline^31^ availble in the following GitHub repository: https://github.com/aryeelab/RNAseq_BE_editing. The dbSNP reference was used to account for natural variation in human samples^67,68^. The following edits to the analysis were made: control (ABE8e only) and *NANOG*-edited hESCs were merged and mapped onto the same space. A genomic position required a minimum coverage of 50 reads to be analysed. To account for remaining SNVs, Single nucleotide variants, PCR or sequencing errors identified in both control and *NANOG*-edited hESCs across all experimental repeats were filtered out.

### Statistical information

The number of cells or embryos analysed (*n*), statistical tests and *P* values are all stated in each figure or figure legend. The tests performed in this paper are unpaired two-tailed Student’s *t*-test. For multiple comparison of RNA-seq analysis, false discovery rates were controlled with the Benjamini–Hochberg method (false discovery rate (FDR) < 0.05). Differential gene expression of scRNA-seq data was performed using the MAST test. Differential gene expression of bulk RNA-seq data was performed using DESeq2. Data are represented as mean ± s.d. Unless otherwise noted, each experiment was performed at least three times. Statistical methods were not used to predetermine sample size for experiments and experiments were not performed blinded. 

### Reporting summary

Further information on research design is available in the Nature Portfolio Reporting Summary linked to this article.

## Online content

Any methods, additional references, Nature Portfolio reporting summaries, source data, extended data, supplementary information, acknowledgements, peer review information; details of author contributions and competing interests; and statements of data and code availability are available at https://doi.org/10.1038/s41586-026-10792-1.

## Supplementary information

Reporting Summary

Supplementary Table 1 Differentially expressed genes comparing *NANOG*-edited versus *AAVS1*-control hESCs.

Supplementary Table 2 Differentially expressed genes comparing homozygous *NANOG*-edited human embryo cells versus *AAVS1*-edited control human embryo cells.

Supplementary Table 3 Differentially expressed genes comparing *Nanog*-edited versus *Aavs1*-control mouse embryos.

Supplementary Table 4 Gene ontology terms for differentially expressed genes mouse and human embryos following NANOG and AAVS1 editing.

Supplementary Table 5 Count matrix scRNA-seq of NANOG and AAVS1-edited human embryos with annotation of associated cell lineage.

Supplementary Tables 6–9 Supplementary Table 6: sgRNA sequences; Supplementary Table 7: Sequencing primers; Supplementary Table 8: Primary antibodies used in immunofluorescence; Supplementary Table 9: Secondary antibodies used in immunofluorescence.

Peer Review File

## Source data

Source Data Fig. 1

Source Data Fig. 2

Source Data Fig. 3

Source Data Fig. 4, Extended Data Fig. 9

Source Data Extended Data Fig. 1

Source Data Extended Data Fig. 2

Source Data Extended Data Fig. 3

Source Data Extended Data Fig. 4

Source Data Extended Data Fig. 6

## Data Availability

Source data files are provided with this paper for Figs. 1–4 and Extended Data Figs. 1–4, 6 and 9. Raw human and mouse embryo scRNA-seq and hESC RNA-seq data produced for this study are deposited in the Gene Expression Omnibus (GEO) under accessions GSE312160 (hESC), GSE312763 (mouse) and GSE319780 (human). Raw mouse and human embryo and hESC target amplicon sequencing datasets are available from Sequence Read Archive (SRA) database BioProject accession number PRJNA1431218. Count matrix of scRNA-seq analysis of *NANOG*- and AAVS1-edited human embryos with annotation of associated cell lineage is provided in Supplementary Table 5. The raw whole-genome sequencing data from human preimplantation embryos cannot be shared publicly to protect the privacy of the individuals or couples that donated their eggs, sperm or embryos to the study. Donor consent of the samples from which this data were generated does not allow for broad sharing of the raw whole-genome sequencing data. The anonymized raw whole-genome sequencing data from human preimplantation embryos may be requested through the corresponding author and by application to the University of Cambridge Research Office contracts team (sbscontracts@admin.cam.ac.uk). The data can be shared following signatures of the data transfer agreement by the academic institutions of the requester and corresponding author. The turnaround time for processing the initial application is four weeks. The data will be available at the University of Cambridge during the legal data retention time frame, which is ten years after completion of the project or publication of associated findings. Plotted low-pass whole-genome sequencing data are available from Figshare (https://doi.org/10.6084/m9.figshare.32336139 (ref. ^69^)). Raw human embryo microscopy data are available from Figshare (https://doi.org/10.6084/m9.figshare.32336139 (ref. ^69^)). Previously published single-cell RNA-seq data from human embryos were downloaded from GEO accessions GSE36552 and GSE66507 and at EMBL-EBI ArrayExpress accession number E-MTAB-3929. Previously published single-cell RNA-seq data from mouse embryos were downloaded from GEO accession GSE123046. Previously published single-cell or bulk RNA-seq data from human pluripotent stem cells were downloaded from GEO accessions GSE250613, GSE250614, GSE126488, GSE69647 and GSE138304 and at EMBL-EBI ArrayExpress accession numbers E-MTAB-2857, E-MTAB-4461 and E-MTAB-10914. Source data are provided with this paper.

## Code availability

Sequencing analysis pipeline, code and source data for this paper are available on Github at https://github.com/oliverbower/Bower_et_al_analysis.
