# Supplementary material for: Base editing reveals an essential role for NANOG in human embryogenesis
Source: Nature. 2026 Jun 25;658(8138):1281–8. doi: 10.1038/s41586-026-10792-1 (PMC13716833; doi:10.1038/s41586-026-10792-1)
Supplement: Supplementary file 1 [file 41586_2026_10792_MOESM1_ESM.pdf]

nature research

Corresponding author(s): Kathy Niakan and Katarina Harasimov

Last updated by author(s): May 20, 2026

Reporting Summary

Nature Research wishes to improve the reproducibility of the work that we publish. This form provides structure for consistency and transparency in reporting. For further information on Nature Research policies, see Authors & Referees and the Editorial Policy Checklist.

Statistics

For all statistical analyses, confirm that the following items are present in the figure legend, table legend, main text, or Methods section.

n/a	Confirmed	
☐	☒	The exact sample size (*n*) for each experimental group/condition, given as a discrete number and unit of measurement
☐	☒	A statement on whether measurements were taken from distinct samples or whether the same sample was measured repeatedly
☐	☒	The statistical test(s) used AND whether they are one- or two-sided<br>*Only common tests should be described solely by name; describe more complex techniques in the Methods section.*
☒	☐	A description of all covariates tested
☐	☒	A description of any assumptions or corrections, such as tests of normality and adjustment for multiple comparisons
☐	☒	A full description of the statistical parameters including central tendency (e.g. means) or other basic estimates (e.g. regression coefficient) AND variation (e.g. standard deviation) or associated estimates of uncertainty (e.g. confidence intervals)
☐	☒	For null hypothesis testing, the test statistic (e.g. *F*, *t*, *r*) with confidence intervals, effect sizes, degrees of freedom and *P* value noted<br>*Give P values as exact values whenever suitable.*
☒	☐	For Bayesian analysis, information on the choice of priors and Markov chain Monte Carlo settings
☒	☐	For hierarchical and complex designs, identification of the appropriate level for tests and full reporting of outcomes
☒	☐	Estimates of effect sizes (e.g. Cohen's *d*, Pearson's *r*), indicating how they were calculated

Our web collection on statistics for biologists contains articles on many of the points above.

Software and code

Policy information about availability of computer code

Data collection	All the data that support the findings of this study are shown in the Figures and Extended Data Figures. Target amplicon sequencing was performed either by Sanger sequencing or Illumina MiSeq platform with (paired end reads of 250 base pairs). Confocal images were acquired with an SP8 confocal laser scanning microscope (Leica) using a plan apochromatic 40x 1.10 NA water-immersion objective or a 10x or 20x oil immersion objective. For bulk RNAseq of hESCs, sequencing was performed using a NovaSeq 6000 (Illumina) with average sequencing depth of 25 million reads. For low-pass whole genome sequencing, samples were sequenced using a MiSeq sequencing (Illumina) platform to approximately 1x coverage. Single-cell RNAsequencing was carried out on NovaSeq 6000 to a depth of ~12 million reads per cell.
Data analysis	Sequencing analysis pipelines and codes used in this paper are available on Github at: https://github.com/oliverbower/Bower_et_al_analysis. No custom code was central to the conclusions of the paper.<br><br>Bioinformatic analysis of sgRNA efficiency<br>FASTQ alignment was performed using BWA to map paired end reads to the genome. SAMtools was used for processing. Analysis was performed using the CrispRVariants package in R and scripts from the following GitHub repository: https://github.com/jzohren/crispr-miseq. To control for PCR amplification and sequencing errors, Cas9 edited mouse embryo reads had uncategorised ('Other') reads and single nucleotide variants of <1% each incidence filtered, and mouse embryo and hESC adenine base editing reads were assessed for editing at the on-target and bystander bases only. Human off-target DNA editing was assessed using CRISPResso2.<br><br>Bioinformatic analysis of single cell RNAseq<br>Cutadapt version 4.2 package was used to remove sequencing adaptors from FASTQ files of single cell RNAseq reads, with minimum read length set to 50. FastQ Screen version 0.13.0 and FastQC version 0.11.8 were used for assessing read quality. Alignment was performed using HISAT2 version 2.1.0 for mouse, and STARsolo 2.7.1b for human. The mouse genome (Mus musculus GRCm38 release-89) or the human genome (Homo sapiens GRCh38 release-89) were used. For mouse, BAM files were generated using SAMtools version 1.18. Count matrices were generated using HTseq version 2.0.5. Integration with previously published human embryo samples was performed using Seurat version 5.0.2. Dimensionality reduction and feature expression was performed using the R package Seurat version 5.0.2. For

mouse cells, sample filtering was performed to exclude cells with less than 5000 features, greater than 5% mitochondrial read content, and cells with less than 5M or greater than 20M total RNA counts. For human cells, sample filtering was performed to exclude cells with less than 1000 features, greater than 20% mitochondrial read content, and cells with less than 20,000 total RNA counts. Features expressed in less than 3 cells were also excluded. Cells were clustered using the Seurat FindNeighbors and FindClusters functions. Differential gene expression was performed with Seurat using the MAST test. Gene set enrichment analysis was performed using Enrichr.

Bioinformatic analysis of bulk RNAseq
FASTQ files of bulk RNA reads were trimmed, aligned and a count matrix was generation using the methods described for single cell RNAseq. Differential gene expression was performed using the R package DESeq2 version 1.42.1. Features expressed in less than 3 samples or with less than 10 counts were removed. False discovery rate was calculated using the Benjamini-Hochberg method. Gene set enrichment analysis was performed using Enrichr.

RNA off-target editing analysis
RNA off-target editing was performed using a pipeline outlined in the following GitHub repository: https://github.com/aryeelab/RNAseq_BE_editing. The dbSNP reference was used to account for natural variation in human samples. The following edits to the analysis were made: Control (ABE8e only) and NANOG-edited hESCs were full merged and mapped onto the same space. A genomic position required a minimum coverage of 50 reads to be analysed. Single nucleotide variants, PCR or sequencing errors identified in both control and NANOG-edited hESCs across all experimental repeats were filtered out.

For manuscripts utilizing custom algorithms or software that are central to the research but not yet described in published literature, software must be made available to editors/reviewers. We strongly encourage code deposition in a community repository (e.g. GitHub). See the Nature Research guidelines for submitting code & software for further information.

Data

Policy information about availability of data

All manuscripts must include a data availability statement. This statement should provide the following information, where applicable:

- Accession codes, unique identifiers, or web links for publicly available datasets
- A list of figures that have associated raw data
- A description of any restrictions on data availability

Source data files are provided with this paper for Figures 1-4 and Extended Data Figures 1-4, 6 and 9. Raw embryo scRNAseq and hESC RNAseq produced for this study are deposited in the GEO database accession numbers: GSE312160, GSE312763, GSE319780, and SRA database BioProject accession number: PRJNA1431218. Count matrix of single cell RNAseq analysis of NANOG and AAVS1 edited human embryos with annotation of associated cell lineage is provided in Supplementary Table 5. Raw low-pass whole genome sequencing data from human preimplantation embryos cannot be made publicly available due to sensitive genomic information, ethical restrictions and the terms of donor informed consent. Raw human embryo microscopy data is available from Figshare: https://doi.org/10.6084/m9.figshare.32336139. Previously published single-cell RNA-seq data from human embryos were downloaded from GEO accessions GSE36552 [https://www.ncbi.nlm.nih.gov/geo/query/acc.cgi?acc=GSE36552] and GSE66507 [https://www.ncbi.nlm.nih.gov/geo/query/acc.cgi?acc=GSE66507] and at EMBL-EBI ArrayExpress accession number E-MTAB-3929 [https://www.ebi.ac.uk/arrayexpress/experiments/E-MTAB-3929/]. Previously published single-cell RNA-seq data from mouse embryos were downloaded from GEO accession GSE123046 [https://www.ncbi.nlm.nih.gov/geo/query/acc.cgi?acc=GSE123046]. Previously published single-cell or bulk RNA-seq data from human pluripotent stem cells were downloaded from GEO accessions GSE250613 [https://www.ncbi.nlm.nih.gov/geo/query/acc.cgi?acc=GSE250613], GSE250614 [https://www.ncbi.nlm.nih.gov/geo/query/acc.cgi?acc=GSE250614], GSE126488 [https://www.ncbi.nlm.nih.gov/geo/query/acc.cgi?acc=GSE126488], GSE69647 [https://www.ncbi.nlm.nih.gov/geo/query/acc.cgi?acc=GSE69647] and GSE138304 [https://www.ncbi.nlm.nih.gov/geo/query/acc.cgi?acc=GSE138304] and at EMBL-EBI ArrayExpress accession numbers E-MTAB-2857 [https://www.ebi.ac.uk/biostudies/studies?query=E-MTAB-2857], E-MTAB-4461 [https://www.ebi.ac.uk/biostudies/arrayexpress/studies/E-MTAB-4461] and E-MTAB-10914 [https://www.ebi.ac.uk/biostudies/ArrayExpress/studies/E-MTAB-10914?query=E-MTAB-10914].

Field-specific reporting

Please select the one below that is the best fit for your research. If you are not sure, read the appropriate sections before making your selection.

☒ Life sciences ☐ Behavioural & social sciences ☐ Ecological, evolutionary & environmental sciences

For a reference copy of the document with all sections, see nature.com/documents/nr-reporting-summary-flat.pdf

Life sciences study design

All studies must disclose on these points even when the disclosure is negative.

Sample size	No statistical methods were used to predetermine sample size. Sample size was determined based on our previous experience and experience from other groups' work.<br>For human embryos and embryonic stem cells:<br>Fogarty et al., Nature, 2017<br>Blakeley et al., Development, 2015<br>Niakan et al., Developmental Biology, 2013<br>For mouse embryos:<br>Hirate et al., Current Biology, 2013<br>Frum et al., Elife, 2018
Data exclusions	No data were excluded from the study design.

Replication	Experiment were replicated at least three times and the data were reproducible in the different attempts of replication. All attempts at replication were successful.
Randomization	Samples were allocated randomly into experimental groups.
Blinding	Blinding was not applied to experiments because group allocation was determined by predefined experimental variables such as sibling-matched control and experimental groups, developmental stage or cell culture conditions.

Reporting for specific materials, systems and methods

We require information from authors about some types of materials, experimental systems and methods used in many studies. Here, indicate whether each material, system or method listed is relevant to your study. If you are not sure if a list item applies to your research, read the appropriate section before selecting a response.

Materials & experimental systems

n/a	Involved in the study
☐	☒ Antibodies
☐	☒ Eukaryotic cell lines
☒	☐ Palaeontology
☐	☒ Animals and other organisms
☐	☒ Human research participants
☒	☐ Clinical data

Methods

n/a	Involved in the study
☒	☐ ChIP-seq
☒	☐ Flow cytometry
☒	☐ MRI-based neuroimaging

Antibodies

Antibodies used	A list of all antibodies used, catalog number and concentrations is provided in Supplementary Tables 8 and 9.
Validation	Primary antibodies:<br>Anti-OCT4, SC-5279 https://www.scbt.com/p/oct-3-4-antibody-c-10?srsltid=AfmBOorrPN-rOMHyIwSasYFHfehJQZbKTG7MrCHK32KMsnKb7vlDU9h-<br>Anti-NANOG, AF1997: https://www.rndsystems.com/products/human-nanog-antibody_af1997?gclsrc=aw.ds&gad_source=1&gad_campaignid=20161432922&gbraid=0AAAAAD_kmX25MH-8W-pqYW0sN3pD3Gdu5&gclid=EAIaIQobChMIyYG15oXIlAMVVJtQBh2j0jyhEAAYASAAEgKvnvD_BwE<br>Anti-NANOG, ab21624: https://www.abcam.com/en-us/products/primary-antibodies/nanog-antibody-ab21624<br>Anti-NANOG, 14-5761-80: https://www.thermofisher.com/antibody/product/Nanog-Antibody-clone-eBioMLC-51-Monoclonal/14-5761-80<br>Anti-GATA4, SC-9053: https://www.scbt.com/p/gata-4-antibody-h-112?srsltid=AfmBOopo-BZi8IdH23DN2zSMD8lInnjHuZ8BHhNyMkRhoFQVmCmYRi0b<br>Anti-GATA4, 14-9980-82: https://www.thermofisher.com/antibody/product/Gata-4-Antibody-clone-eBioEvan-Monoclonal/14-9980-82<br>Anti-CDX2, MU392A-UC: https://biogenex.com/product/anti-cdx-2/<br>Anti-GATA3, ab199428: https://www.abcam.com/en-us/products/primary-antibodies/gata3-antibody-epr16651-chip-grade-ab199428

Eukaryotic cell lines

Policy information about cell lines

Cell line source(s)	We used the H9 human ESC line obtained under licence and SLA agreement with WiCell.
Authentication	STA profiling was performed.
Mycoplasma contamination	The cell lines were routinely tested for mycoplasma and were found to be negative.
Commonly misidentified lines (See ICLAC register)	No commonly misidentified cell lines were used in the study.

Animals and other organisms

Policy information about studies involving animals; ARRIVE guidelines recommended for reporting animal research

Laboratory animals	Four- to eight-week-old (C57BL6×CBA) F1 female mice were super-ovulated using injection of 5 IU of pregnant mare serum gonadotrophin (PMSG; Sigma-Aldrich). Forty-eight hours after PMSG injection, 5 IU of human chorionic gonadotrophin (HCG; Sigma-Aldrich) was administered. Superovulated females were set up for mating with 2-12-month-old (C57BL6×CBA) F1 males. At the University of Cambridge Physiology, Development and Neuroscience Department, CD1 10-12-week females were naturally

	mated to CD1 6-12-month-old males mice. Mice were maintained on a 12 h light–dark cycle, ambient temperature 19/22°C, and humidity 45/65%.
Wild animals	No wild animals were used in the study.
Field-collected samples	No field collected samples were used in the study.
Ethics oversight	The study was approved by the Animal Ethics Committee and by the UK Home Office licence number 70/8560, following all relevant institutional and national guidelines and regulations.

Note that full information on the approval of the study protocol must also be provided in the manuscript.

Human research participants

Policy information about studies involving human research participants

Population characteristics	No sex or gender based analysis was performed on early human embryos or gametes that were consented across diverse backgrounds from clinics in Cambridge, Newcastle and London.
Recruitment	Informed consent was obtained from all donors by suitable qualified clinic staff not involved in the research or directly involved in treatment. Human oocytes, gametes and embryos donated as supernummary to assisted conception treatment requirements were not financially compensated. Alternatively, human oocytes were donated as part of an 'egg sharing' programme or by non-patient donors. A subsidy of £1,500 or an additional cycle of treatment was provided to donations made through the 'egg sharing' programme while non-patient donors received a £500 financial compensation per donation cycle. Before giving consent, donors were provided with all of the necessary information about the research project, including details of the biochemical and genetic studies, such as genome editing, that would be performed.
Ethics oversight	This study was approved by the UK Human Fertilisation and Embryology Authority (HFEA): under research license numbers R0162 and R0152 at centres 0397 and 0401 and independently reviewed by the Health Research Authority's Research Ethics Committee IRAS projects 308099, 252286 and 272218.

Note that full information on the approval of the study protocol must also be provided in the manuscript.
