# Supplementary material for: Base editing reveals an essential role for NANOG in human embryogenesis
Source: Nature. 2026 Jun 25;658(8138):1281–8. doi: 10.1038/s41586-026-10792-1 (PMC13716833; doi:10.1038/s41586-026-10792-1)
Supplement: Supplementary file 2 [file 41586_2026_10792_MOESM8_ESM.pdf]

nature portfolio

Peer Review File

Base editing reveals an essential role for NANOG in human embryogenesis

Corresponding Author: Professor Kathy Niakan

Any redactions in this file are there to maintain patient confidentiality, the confidentiality of unpublished data, or to remove third-party material.

This file contains all reviewer reports in order by version, followed by all author rebuttals in order by version.

Version 0:

Reviewer comments:

Referee #1

(Remarks to the Author)
The manuscript by Bower et al, which is clearly written and contains high quality data, represents a multi-lab collaboration characterised by world-leading expertise in genome editing of human embryos (Niakan), human embryo/egg research (Niakan, Herbert) and genome editing/base editing technology (Liu – who invented base editing) and their colleagues. This is an ideal combination of talents to explore the use of base editing to perform gene function studies in human embryos, which Niakan previously attempted (on POU5F1), but using the (subsequently identified) sub-optimal (at least in embryos) CRISPR/Cas9 methodology based on double-strand breaks. This report essentially describes an attempt to do it another way (on a different gene). The success of the base editing strategy is noteworthy, given the relative failure of the earlier strategy and will no doubt (and justifiably) attract much attention.

Comments on ethical acceptability
The research described here - genetic alteration of embryos - falls into Category 2 of the ISSCR's guidelines. Note: this is not heritable human genome editing (HHGE), which would require transfer of an altered embryo to a uterus (ISSCR Cat 3A, not permitted – and unlawful in the UK anyway). Based on this categorisation, the ISSCR states that human embryo research is permitted only after review and approval through a specialised scientific and ethics review process.
This research is (easily) compliant with these guidelines and local (statutory) regulations, having been: i) licenced (all paperwork available) by the UK Human Fertilisation & Embryology Authority (HFEA), the statutory regulator of human embryo research, whose Licence Committee (following review of the application by independent experts) approves a research licence to perform embryo research only after careful consideration of the scientific rationale for doing so i.e. the potential benefits (and harms) of the research (the principle of proportionality) and the necessity to use (appropriate numbers of) human embryos i.e. the unavailability of suitable alternatives (sometimes called the principle of subsidiarity). The creation of human embryos is also permitted in this UK framework, if this is required, as it is here (to ensure editing occurs at the earliest opportunity) and this activity is explicitly justified in accompanying documents seen by other assessors; and ii) granted ethical approval by the UK Health Research Authority's Research Ethics Committee (paperwork also supplied). The HFEA inspection reports for the project also indicate the research is compliant with the Act/Code of Practice.
This research requires donation of human oocytes – consent to donation was obtained and forms/information sheets (for gametes and embryos – all appropriate) are included. This research does not culture embryos beyond 14-days post-fertilisation.
Note also that the work was supported by the Wellcome Human Developmental Biology Initiative (and other major UK funders), which would also have required an ethical assessment of the rationale and experimental design.
The authors supply a list of members comprising the various committees (at least 3) that assessed their research proposals and the composition of these is entirely appropriate.
There is a useful ethics statement in the manuscript summarising some of this.
Finally: the authors have ensured that the research is compliant with a series of ethical norms established by different institutions, nationally and internationally – it is not their job here to resolve fundamental questions concerning the moral status of the human embryo. In the UK, whatever that status, the human embryo is the sort of thing that can be lawfully created and experimented on in the interests of the greater good (public interest).

General comments on the manuscript

There is a reassuring narrative in the manuscript, familiar from the previous work of Niakan with human embryos. The base editing technical aspects (sgRNA efficiency etc) are first addressed in human ES cells – which are also analysed to examine the role of NANOG in this context; mouse embryos are then used to test the effectiveness of this base editor in a mammalian embryo. Only when these components are completed satisfactorily are human embryos used to generate knowledge about human embryos (which cannot be guaranteed by using hES cells or mouse embryos). This is an example of the 'principle of subsidiarity', which requires that entities of a lower 'moral status' are used to answer scientific questions (where possible/feasible) before using entities of higher moral status (here, human embryos). Of course, this assumed hierarchy of moral status is contestable. Minimising the use of human embryos in this way is evidence of taking the special status of human embryos (in UK law) seriously, but it is also pragmatic: human embryos are not easy to source and are a rare, thus precious, commodity. Having said that, I'm not sure I would use the word 'precious' in the manuscript (l. 84, 132), since in addition to my use of it to highlight the limited numbers available, some might use it to denote an inherent moral status that is incompatible with research i.e. with the instrumentalization of human embryos. Perhaps best to delete or use another word ('valuable'?) But I leave it to the authors to decide.

The comparative analysis of the human and mouse embryo phenotypes makes for an interesting read – and the species-specific roles of NANOG described are important, given that manipulating the fate of human pluripotent stem cells in regenerative medicine requires understanding human gene networks and cannot simply rely on knowledge from the mouse. It is also good to see that the speculation at the end of the manuscript about potential clinical applications (mutation correction) are tempered by the insistence that future clinical use is a decision for society and not just scientists, based on ethical inquiry, not just scientific feasibility.

Specific comments/questions

I'm slightly confused about the blastocyst 4 data. The scRNAseq was performed on isolated (enriched) ICMs and the sequencing data suggested blasto 4 is effectively unedited/wild-type or het – but if I understand correctly, 57% of cells of this blasto show trophectoderm clustering. How efficient is the enrichment of ICM?

Why is NANOG expression reduced in NANOG KO hES cells? Nonsense-mediated decay?

The focus on the impact of base editing on the intended target site (and bystander effects) is warranted, given previous problems with loss of heterozygosity etc. But was off-target base editing assessed as a possibility (as opposed to off-target cutting/repair)?

The number of human embryos analysed (transcriptomically) is small (4) and we are an outbred species with genetic variability between us. The upshot is a snapshot of NANOG function in the human embryo, rather than a definitive analysis – would the authors agree? Isn't this a limitation that should be briefly dealt with in the manuscript?

Referee #2

(Remarks to the Author)

In this manuscript, Bower et al. employed ABE8e to edit the NANOG splicing site, thereby disrupting its function. Their findings elucidate NANOG's pivotal role in human embryonic lineage specification, revealing that human pluripotent cells require NANOG to sustain their identity—a requirement distinct from that observed in mouse embryonic stem cells. Notably, NANOG-edited human embryos retained endoderm cells despite the absence of the critical factor FGF4, with IGF1 mediating functional compensation. These unexpected results challenge existing paradigms and underscore the need for further investigation. The following points are suggested to revised the manuscript:

Major comments

1. In Ext. Data Fig. 1b, the efficiency of ABE8e alone appears higher than that of ABE8e with sg4. This raises the possibility of sgRNA-independent off-target activity, which should be examined. Similarly, in Ext. Data Fig. 1e, please clarify why editing the AAVS1 site in a single-cell clone resulted in an SNV in NANOG.

2. Following ABE8e editing of embryos, no RNA-level off-target analysis is presented. It is recommended to assess the events of RNA off-target editing.

3. The substantial differences in editing efficiency between Blastocyst 4 and Blastocysts 1–3 require explanation. Additionally, in Ext. Data Fig. 4a,b, the wild-type data should be presented separately from that of Blastocyst 4 for clarity.

4. In Ext. Data Fig. 5a,b, the authors used an spCas9 strategy in mouse zygotes to target the orthologous 3′ region of Nanog exon 1. To ensure the reliability of downstream analyses, please provide DNA-level off-target data to confirm that the results are unaffected by unintended edits.

5. Given that CRISPR/Cas9 mutagenesis can induce genomic instability, chromosomal rearrangements, and unintended on-target changes, could alternative editors—such as cytosine base editors or prime editors—be considered to introduce stop codons or deletions in Nanog in mouse zygotes, thus aligning more closely with the editing strategy used for NANOG disruption in human embryos?

6. Beyond RTK signaling, could additional key pathways or alternative compensatory factors be identified?

7. Following the detection of IGF1, please discuss its potential implications for future disease research or therapeutic applications.

Referee #3

(Remarks to the Author)
Summary
The authors present a novel application of adenine base editing (ABE8e) to knockout the transcription factor NANOG in human embryos, achieving this without detectable genotoxic effects. It is stated that the loss of NANOG disrupts the formation of the pluripotent epiblast, instead biasing cells toward yolk sac or placental-like lineages. Notably, the study shows evidence of a functional compensation mechanism in humans for primitive endoderm formation in the absence of NANOG, which is partially mediated by IGF1 signaling. These observations diverge from established mouse models, underscoring species-specific developmental pathways and reinforcing the critical importance of direct investigation in human embryos. The work also claims the potential of ABE8e's value for safe, targeted studies in human developmental biology.

Major comments

Although the use of base editing for functional KO studies in human embryos is original, a major issue when using gene editing in the human germline, remains the full efficiency of the technique, which requires analysis on different levels on a sufficient number of embryos. The ABE induces a splice donor site mutation (GT→GC) but does not show direct evidence of intron retention, exon skipping, or aberrant splicing in NANOG transcripts in embryos. Base editing at splice sites may not always abolish gene function; transcripts might still be produced with cryptic splicing. I suggest that the authors provide direct evidence of aberrant splicing (e.g., RT-PCR or NANOG-specific transcript reconstruction from scRNA-seq) to confirm splicing disruption and absence of functional transcripts. The authors have provided some evidence on the genomic and transcriptomic level for this, but show that a full knock-out is very hard to obtain in human embryos, with only 1 successful embryo (almost) fully edited. So all the next comparisons of transcription and species differences are biased by this low full KO editing efficiency, because of mosaic gene editing events which is a major flaw in this study. Furthermore, in the single-cell RNA-seq analysis, cells containing NANOG transcript reads were filtered out. However, it remains unclear how the authors confirmed that the retained cells were indeed fully edited, rather than unedited or TE cells. Additional clarification or validation is needed to ensure that the single-cell data analyzed truly reflect NANOG-deficient cells and not a mixture of cell types or editing states. It is unclear why the authors did not perform basic NANOG protein immunostaining in human embryos, which could have been readily combined with genomic-level editing verification. Such an approach would have provided direct evidence of functional protein loss and helped validate the claimed knockout phenotype. Given the already low editing efficiency, the scarcity of human oocytes available for research is understandable. However, the number of human embryos analyzed in this study is extremely limited and insufficient to robustly support the authors' conclusions. Several key pieces of information are missing, which undermines the strength of the claims made (see further comments below). I strongly recommend that the authors expand their analysis to include a larger number of edited human embryos to allow for more statistically and biologically meaningful interpretations. Furthermore, the current data do not support the conclusion that ABE is highly efficient in this context, and I suggest that this statement be moderated accordingly. In addition, appropriate controls in human embryos are lacking. Even in the mouse embryo experiments, the number of embryos analyzed is very limited, raising concerns about the reproducibility and generalizability of the findings. Additional biological replicates and inclusion of proper negative and positive controls are needed to strengthen the conclusions drawn from both human and mouse data.

Secondly, the manuscript lacks critical information regarding the accuracy of the microdissection used to separate the two (or three) lineages of the human blastocyst. This is a technically challenging procedure that relies on clear identification of the ICM, which is not always visually discernible. In Figure 2a, it appears that laser dissection was used to isolate only a portion of the blastocyst, likely containing a mixture of cell types, including TE cells, rather than specifically isolating distinct lineages. This raises concerns about the purity of the dissected populations. Given the low editing efficiency and the likelihood of mosaicism, how do the authors distinguish transcriptional effects of NANOG editing from those arising due to TE contamination? Is lineage assignment inferred solely based on the absence or reduced number of NANOG transcript reads? If so, this is not a reliable indicator of editing success or lineage identity. Additional clarification on the lineage identification strategy, preferably supported by lineage-specific markers or single-cell-level annotation, is needed to validate these interpretations.

Finally, the mechanistic experiment involving IGF1 treatment was conducted on a very small number of embryos, which limits the ability to draw robust conclusions. The observed effects should be interpreted with caution, and the authors should either increase the number of biological replicates or clearly acknowledge the limitations of these preliminary findings.

Specific comments

- Regarding the sequencing depth (scRNAseq) of the blastocysts: While the authors report average read counts per cell at the NANOG locus, this coverage is likely insufficient for robust base-level genotyping in single cells. Genotype assignments (WT vs edited vs unclear vs absent) should be accompanied by read count thresholds to increase confidence, particularly in

cells where expression is low due to the expected functional knockout.

- Fig 2 (following previous comment): The substantial mosaicism observed within TE (Fig. 2e) raises the possibility that similar mosaicism exists within the ICM. This could affect the interpretation of lineage specification defects as being solely due to complete loss of NANOG. Additional analysis of mosaicism in ICM cells would strengthen the conclusions.

- Fig 2: Why did the authors decide to not perform scRNAseq of blastocyst 5, since it carries a more homozygous genotype that could therefore strengthen the findings of the study?

- Fig 2: The authors should also include the whole-genome sequencing analysis of blastocyst 3, given that this is the only homozygous mutant.

- The study showed a reduction in OCT4 protein in NANOG-/- hESCs, but not in their transcriptional data. Could the authors elaborate on this in the discussion?

- What are the scores of human embryos and more specifically the ICM vs TE in the human embryo experiments? As timelapse imaging was performed, this should be very feasible. From the pictures, you only see an obvious ICM in blastocyst

- Can the authors clarify/extend the mention of a 'diapause like state' upon IGF1 treatment? Do treated embryos exhibit decreased proliferation?

- Extended Data Figure 4: Genotyping NANOG-targeted human embryos at the blastocyst stage: why only data for 3 embryos?

- Extended Data Figure 9: Differences and similarities in expression of receptor tyrosine kinase (RTK) signalling components and primitive endoderm-associated genes in human versus mouse NANOG/Nanog-edited embryos. Also including not fully edited mouse/human embryos?

Referee #4

(Remarks to the Author)
This study by Bower et al. investigates the role of the pluripotency-associated transcription factor NANOG in early human embryos using adenine base editor ABE8e, a next-generation genome-editing tool that avoids DNA double-strand breaks and the associated risks of genotoxicity and aneuploidy inherent to conventional CRISPR/Cas9 approaches. To the best of my knowledge, this represents the first successful application of base editing for functional interrogation of gene activity in human embryos. By precisely converting the canonical "GT" splice donor site in exon 1 of NANOG to "GC," the authors efficiently achieved a loss-of-function mutation. After validating this editing strategy in human embryonic stem cells (ESCs), they applied it to human MII-stage oocytes by co-injection with sperms and cultured them to the blastocyst stage. Genotyping confirmed efficient and accurate base editing, with mutant embryos progressing morphologically to the blastocyst stage. However, single-cell transcriptomic analysis revealed a profound disruption in epiblast formation: the NANOG-edited embryos failed to establish a pluripotent epiblast and instead upregulated gene programs characteristic of the trophectoderm and hypoblast lineages. These results clearly demonstrate that NANOG is indispensable for human epiblast fate acquisition and pluripotency maintenance. Importantly, the findings also highlight key species-specific regulatory differences. Unlike mouse Nanog mutants—which fail to specify either epiblast or primitive endoderm—NANOG-deficient human embryos still gave rise to hypoblast-like cells, possibly due to compensatory IGF signaling that circumvents the requirement for NANOG-induced FGF4. Altogether, this study underscores the necessity of studying human development directly in human embryos or primate models, as rodent systems do not fully capture the nuances of human pluripotency regulation. It also provides valuable insight into the molecular mechanisms that govern early lineage segregation and has broader implications for understanding human infertility, improving stem cell models, and evaluating the safety of germline genome editing. That said, despite the significance of the findings, the current study does not sufficiently assess the genomic and epigenetic safety profile of ABE8e in human embryos. As it stands, these safety concerns, along with other major concerns, preclude a recommendation for publication in Nature in its current form.
Major concerns:
1. The manuscript emphasizes the relative safety of adenine base editing (ABE) compared to CRISPR/Cas9-mediated editing, however, this claim is not adequately supported by evidence. Given the potential translational implications of applying ABE for germline gene correction—as noted by the authors in the discussion section ("our approach also establishes a framework for disease modeling and potential therapeutic correction through base editing")—it is critical to rigorously evaluate both the safety and specificity of this approach in human embryos. At present, the manuscript lacks comprehensive off-target analyses and broader assessments of potential genotoxicity, transcriptomic perturbation, or epigenetic dysregulation. To strengthen the study's impact and support its potential translational relevance, the authors are encouraged to incorporate the following safety-related experiments and analyses in a revised version:
a) To support the safety profile of ABE8e in human embryos, the authors should perform high-coverage whole-genome sequencing of edited embryos to detect off-target base edits, indels, or structural variants beyond NANOG. Prior studies in hESCs (PMID: 35991312) found no detectable off-targets using similar methods. Special attention should be given to noncoding regulatory regions (e.g., promoters, enhancers, UTRs) that could impact gene expression and viability. Given that NANOG-null embryos may not yield ESCs, the authors could instead derive trophoblast stem cells (TSCs) from trophectoderm biopsies as a surrogate platform for comprehensive genomic and transcriptomic profiling. If concerns arise about genomic stability during extended hTSC culture, analyses could focus on early-passage cells or blastocyst

outgrowths. For a broader safety assessment of ABE8e, editing a neutral locus such as AAVS1 followed by ESC derivation and comprehensive characterization of edited lines, including off-target analysis, transcriptomic profiling, and epigenetic evaluation, could be considered.
b) While WGS of trophectoderm biopsies (Fig. 2f) assessed aneuploidy and chromosomal loss, these analyses are insufficient to rule out broader genomic instability. Despite avoiding double-strand breaks, ABE8e has been linked to large-scale structural variants—including deletions, duplications, and translocations—in mammalian embryos (PMID: 39529170). To rigorously evaluate genome integrity, the authors should apply structural variant detection pipelines to existing WGS data or perform long-read sequencing to capture complex rearrangements. Even rare events can disrupt cell function, induce apoptosis, or cause mosaicism, posing risks to embryo development. A comprehensive genome-wide analysis is needed to validate editing safety.
c) While the manuscript documents lineage shifts in NANOG-edited embryos, a more comprehensive transcriptomic analysis is warranted. Specifically, ICM-derived cells that adopt trophectoderm-like and hypoblast-like fates should be directly compared to bona fide TE and hypoblast cells from control embryos. Differential gene expression and pathway enrichment analyses could help reveal whether the converted cells retain residual pluripotency signatures or activate aberrant programs, such as DNA damage response, apoptosis, or cell cycle dysregulation—pathways previously linked to ABE-induced aneuploidy (PMID: 39529170). Additionally, integrating these transcriptomic findings with genomic variant data would clarify whether any observed expression changes correlate with potential off-target edits or unintended structural variants.
d) The deaminase in ABE can induce unintended A→G edits in RNA independent of gRNAs. Although transient, such edits may disrupt gene expression. Comparing RNA-seq reads to the reference genome can help detect and quantify RNA off-target effects of ABE.

2. The authors attribute the divergent effects of NANOG loss in mouse and human ESCs to species differences. However, mouse ESCs represent the naïve state, while human ESCs resemble post-implantation, primed epiblasts. To rule out pluripotency-state effects, the authors should evaluate NANOG deficiency in mouse epiblast stem cells and naïve-state human ESCs.

3. The conclusions of altered lineage specification in NANOG-edited embryos rely heavily on scRNA-seq clustering (e.g., Fig. 3) without independent validation. Immunostaining for epiblast, hypoblast/primitive endoderm, and trophectoderm markers in control and NANOG-edited embryos, along with quantification of lineage proportions, is needed to confirm disrupted specification.

4. It remains unclear how NANOG-deficient ICM cells adopt trophectoderm-like and hypoblast-like fates. The authors could explore NANOG knockout in naïve hESCs to gain mechanistic insight into these lineage conversions. Does knocking out NANOG in naïve hESCs also drive the cells to TE-like and hypoblast-like lineages?

5. The authors suggest IGF1 signaling compensates for loss of NANOG/FGF4 for hypoblast specification in humans. This could be directly tested by inhibiting IGF1R in NANOG-deficient embryos. Mouse embryos should be used as controls. Conversely, combining FGF blockade with IGF1 supplementation in WT mouse and human embryos could assess functional redundancy.

5. Given the availability of protocols for culturing human blastocysts beyond implantation (PMIDs: 27144686, 27144363, 31830756), the authors should consider extending culture and analysis of NANOG-edited and control embryos into early post-implantation stages.

Minor concerns:

1. To confirm that observed phenotypes are due to NANOG loss, please perform a rescue experiment by reintroducing NANOG in human ESCs.
2. Co-injection of CRISPR constructs together with sperm into M-II oocytes has previously been used to reduce mosaicism (PMID: 28783728). The authors should cite this paper and briefly comment on this.
3. Include a volcano plot, analogous to Fig. 3f, to illustrate the transcriptional impact of Nanog knockout in mouse blastocysts.
4. Show the effect of IGF1 on primitive endoderm formation in mouse embryos to complement the human data presented in Fig. 4f.
5. Please specify how cell types (e.g., trophectoderm, epiblast, ICM, primitive endoderm) were classified in both human and mouse single-cell RNA-seq datasets.
6. Clarify whether the reported 77.4% efficiency for sgRNA5 (Fig. 1c, Extended Data Fig. 1b) was calculated post-transfection and sorting.
7. Line 90–91 should state that both N-terminal and C-terminal antibodies failed to detect NANOG protein instead of claiming “full-length loss of NANOG protein”.
8. For single-cell RNA-seq data, subdivide edited alleles into synonymous, missense, and other categories. Provide detailed read-level sequences in the supplementary data.
9. For embryos that failed to reach the blastocyst stage (Extended Data Fig. 3b), clarify whether editing efficiency was assessed at the single-blastomere level or not.
10. In Extended Data Fig. 9, INSR and IGF1R appear only modestly enriched in hypoblasts. Please provide IF evidence of their expression in human hypoblasts and compare with mouse embryos.

Referee #5

(Remarks to the Author)
I co-reviewed this manuscript with one of the reviewers who provided the listed reports.

Version 1:

Reviewer comments:

Referee #1

(Remarks to the Author)
The authors have addressed my questions to my satisfaction - the additional data and analyses have improved the manuscript.
One suggestion: on p15 the authors end with a statement about the conditions of legitimate use of embryo genome editing in a clinical context: "Nonetheless, clinical translation of genome editing in human embryos must not proceed unless there is broad societal debate and support" - it would be useful to have a reference to the reports of the two commissions on Human Genome Editing at this point, one of which focussed on heritable uses, since they remain useful reminders of this and other conditions. The citations are:

National Academies of Sciences, Engineering, and Medicine (2017) Human Genome Editing: Science, Ethics, and Governance. Washington, DC: The National Academies Press. doi: https://doi.org/10.17226/24623.

National Academy of Medicine, National Academy of Sciences, and the Royal Society (2020) Heritable Human Genome Editing. Washington, DC: The National Academies Press. https://doi.org/10.17226/25665.

Andy Greenfield

Referee #2

(Remarks to the Author)
The authors have done more experiments and analyses to improve this study and the concerns I raised have been addressed. I recommend the acceptance of this revised manuscript.

Referee #3

(Remarks to the Author)
The revised manuscript has improved substantially following the reviewers' comments. In particular, the authors have increased the number of edited embryos analysed, introduced genomic genotyping of embryos, added immunofluorescence validation of NANOG protein loss and lineage markers, and revised the transcriptomic analyses accordingly. These additions strengthen the central conclusions.
Overall, the study is now considerably stronger and many of the initial concerns have been addressed. However, several points still require clarification or moderation in the manuscript.

1. Evidence for disruption of NANOG splicing: The authors now provide additional evidence suggesting disruption of the NANOG splice donor site, including RNA-seq reads extending into the intron and PCR assays across exon boundaries. Together with the immunofluorescence analysis showing loss of NANOG protein, this provides support for functional disruption of the gene. However, the PCR data presented in Extended Data Fig. 7 appear to show faint bands in the edited samples, suggesting that amplification may not be completely absent. This raises the possibility that low levels of correctly spliced transcript could still be present. The authors should therefore clarify this interpretation and avoid stating that no amplification occurs if weak bands are visible.More broadly, the RNA-level consequences of the splice-site mutation remain somewhat indirect. Visualization of splice junction usage or more explicit characterization of aberrant transcripts would further strengthen the conclusion that the GT→GC mutation produces a complete loss-of-function allele.

2. Interpretation of editing efficiency and mosaicism: The authors now genotype embryos using Sanger sequencing of multiple trophectoderm biopsies and identify embryos classified as homozygous edited. This represents an important improvement compared with the original submission. However, genotype assignment based on a limited number of biopsies cannot fully exclude mosaicism within the remaining embryo. Consequently, it may be premature to conclude that embryos showing homozygous edits in all biopsies represent completely edited embryos. This limitation should be acknowledged more explicitly. Related to this point, statements that mosaicism is reduced or that embryos are fully edited should be carefully phrased given that the analysis is based on biopsies rather than whole-embryo genotyping.

3. Use of arrested embryos to estimate editing efficiency: The authors include analysis of embryos that arrested prior to the blastocyst stage in order to estimate editing efficiency, which is a good idea in se. However, interpretation of editing outcomes in arrested embryos is not straightforward. Arrested embryos frequently exhibit DNA fragmentation or developmental abnormalities, which may complicate interpretation of genotyping results. The authors should clarify the

rationale for including these embryos in the analysis and avoid overinterpreting editing efficiency based on this dataset.

Some minor comments:

1. The immunofluorescence analysis demonstrating loss of NANOG protein together with retention of GATA3 and GATA4 is an important addition and provides independent validation of the single-cell RNA-seq results. This experiment substantially strengthens the manuscript.
2. The manuscript occasionally refers to the editing strategy as achieving “high-efficiency disruption” of NANOG. Given that complete loss of NANOG protein is observed in a subset of embryos, it may be more accurate to moderate this wording.
3. In the description of the editing experiments, the text refers to “delivery 11 of ABE8e protein”. This appears to be a typographical error and should be corrected.

Referee #6

(Remarks to the Author)
In this revised ms, the authors describe the use of base-editing tools to assess the function of the Nanog transcription factor in early human embryos. Overall, as noted by the previous reviewers (I was not one of the previous reviewers), they have provided a careful assessment of the effectiveness of adenine base editing to target a splice donor site in Nanog, which should produce a splicing defect and effectively a null mutation. They tested it first in human ES cells and then in preimplantation human embryos. In an extended series of human embryo studies, they report an editing frequency of 62.5% (10/16 embryos). However, only 3 embryos were homozygous Nanog-/-. The rest were presumably heterozygous or mosaic- thus the overall efficiency of editing is not quite clear.
In the three homozygous embryos, they find reduction in epiblast markers but presence of primitive endoderm and trophectoderm, suggesting that loss of Nanog leads to failure of epiblast development but not of the primitive endoderm. This is in contrast to the mouse, where loss of Nanog leads to loss of both epiblast and primitive endoderm, identifying a clear distinction across the species, likely related to the loss of FGF4 in the mouse and some resistance to this loss in humans. They also provide some analysis of any possible off target editing at either the DNA or RNA level, as well as evidence of low likelihood of aneuploidy.
Overall, it seems that base editing is an appropriate tool for mutational analysis of gene function in early human embryos. The efficiency is reasonable, and any genotoxic effects are reduced compared with CRISPR/Cas9. This study is not designed to assess whether this approach could be used clinically, which would require a lot more analysis than provided here. The authors must make this distinction very clear. On l348 they state that they have established a framework for potential therapeutic correction – I do not believe this is the case-and it is NOT the goal of this study. I would rewrite this section.
Specific Comments
1. L86 and elsewhere. It is stated that the base editing leads to splicing defects and a null allele. It is not quite clear to me exactly what the null allele would be- can this be explained better? Is NMD induced? Has this been assessed? Or is the splicing defect alone enough to produce a null allele?
2. It is hard to work out the exact effect of loss of Nanog on the overall development of the blastocysts. In Fig 2 e-j, we are given cell number counts for different lineages in the edited blastocysts. Overall it does not seem that there is a big difference in overall cell number between wt and edited embryos (Fig2e) and the number of TE and PrE cells do not seem to differ. However, these numbers pool all edited embryos including non-homozygous mutants, making it hard to know what is happening. The most important data must come from the homozygous mutants- and these are presumably the three embryos in Fig 2j that show only GATA4+ cells in the ICM. However, this figure does not provide actual cell counts, just percentages. Thus I am still unclear whether the ICM maintains its cell number but Epiblast cells are redirected to PrE or TE, or whether the overall ICM number is reduced as epiblast cells fail to develop, but PrE and TE cell numbers do not change. These different scenarios lead to different interpretations of the phenotype observed.
3. Terminology needs to be adjusted. In the Abstract, l52, the authors talk about yolk sac or placental-like transcriptional programs, when what they mean is primitive endoderm (hypoblast) and trophectoderm lineage programs. Yolk sac and placenta are multi-lineage organs.
4. Another confusing use of terminology is to call the dissected part of the blastocyst that contains the polar TE and the ICM as the ICM. It actually has all three lineages in it and the single cell RNAseq data has to be segregated to identify the three cell types. I think the previous reviewers were quite confused and so will be the readers of the paper. Please rename this dissected part of the blastocyst- perhaps polar region?
5. L 331 states that the results demonstrate failure to activate epiblast-associated program. Can this actually be demonstrated? It might require looking in earlier stages of development of Nanog -/- embryos. I realize this is not a reasonable request but would suggest some caution in interpreting the current results.

Version 2:

Reviewer comments:

Referee #3

(Remarks to the Author)
The authors have adequately addressed my remaining concerns.

Referee #6

(Remarks to the Author)
The authors have responded well to all the reviewers' comments. I am happy with the revised ms.

Open Access This Peer Review File is licensed under a Creative Commons Attribution 4.0 International License, which permits use, sharing, adaptation, distribution and reproduction in any medium or format, as long as you give appropriate credit to the original author(s) and the source, provide a link to the Creative Commons license, and indicate if changes were made.
In cases where reviewers are anonymous, credit should be given to 'Anonymous Referee' and the source.
The images or other third party material in this Peer Review File are included in the article's Creative Commons license, unless indicated otherwise in a credit line to the material. If material is not included in the article's Creative Commons license and your intended use is not permitted by statutory regulation or exceeds the permitted use, you will need to obtain permission directly from the copyright holder.
To view a copy of this license, visit https://creativecommons.org/licenses/by/4.0/

Response to editor and peer reviewers

Your manuscript, "Base editing reveals an essential role for NANOG in human embryo lineage specification", has now been seen by 5 referees, whose comments are attached below. While they find your work of potential interest, as do we, they have raised significant concerns that in our view need to be addressed before we can consider publication in Nature.

Should further experiments allow you to address these criticisms, we would be happy to consider a revised manuscript (unless something similar has been accepted at Nature or appeared elsewhere in the meantime). We hope to receive your revised paper within four to six months. If you cannot complete the required revisions within this time frame, please let us know when you would anticipate being able to submit a revised manuscript.

We thank the peer reviewers for their constructive suggestions to improve the manuscript and for the opportunity to resubmit a revised version. We have significantly amended the figures, included additional experiments and replicates and revised the manuscript to take on board their comments.

Referees' comments:

Referee #1 (Remarks to the Author):

The manuscript by Bower et al, which is clearly written and contains high quality data, represents a multi-lab collaboration characterised by world-leading expertise in genome editing of human embryos (Niakan), human embryo/egg research (Niakan, Herbert) and genome editing/base editing technology (Liu – who invented base editing) and their colleagues. This is an ideal combination of talents to explore the use of base editing to perform gene function studies in human embryos, which Niakan previously attempted (on POU5F1), but using the (subsequently identified) sub-optimal (at least in embryos) CRISPR/Cas9 methodology based on double-strand breaks. This report essentially describes an attempt to do it another way (on a different gene). The success of the base editing strategy is noteworthy, given the relative failure of the earlier strategy and will no doubt (and justifiably) attract much attention.

Comments on ethical acceptability

The research described here - genetic alteration of embryos - falls into Category 2 of the ISSCR's guidelines. Note: this is not heritable human genome editing (HHGE), which would require transfer of an altered embryo to a uterus (ISSCR Cat 3A, not permitted – and unlawful in the UK anyway). Based on this categorisation, the ISSCR states that human embryo research is permitted only after review and approval through a specialised scientific and ethics review process.

This research is (easily) compliant with these guidelines and local (statutory) regulations, having been: i) licenced (all paperwork available) by the UK Human Fertilisation & Embryology Authority (HFEA), the statutory regulator of human embryo research, whose Licence Committee (following review of the application by independent experts) approves a research licence to perform embryo research only after careful consideration

of the scientific rationale for doing so i.e. the potential benefits (and harms) of the research (the principle of proportionality) and the necessity to use (appropriate numbers of) human embryos i.e. the unavailability of suitable alternatives (sometimes called the principle of subsidiarity). The creation of human embryos is also permitted in this UK framework, if this is required, as it is here (to ensure editing occurs at the earliest opportunity) and this activity is explicitly justified in accompanying documents seen by other assessors; and ii) granted ethical approval by the UK Health Research Authority's Research Ethics Committee (paperwork also supplied). The HFEA inspection reports for the project also indicate the research is compliant with the Act/Code of Practice.
This research requires donation of human oocytes – consent to donation was obtained and forms/information sheets (for gametes and embryos – all appropriate) are included.
This research does not culture embryos beyond 14-days post-fertilisation. Note also that the work was supported by the Wellcome Human Developmental Biology Initiative (and other major UK funders), which would also have required an ethical assessment of the rationale and experimental design.
The authors supply a list of members comprising the various committees (at least 3) that assessed their research proposals and the composition of these is entirely appropriate.
There is a useful ethics statement in the manuscript summarising some of this.
Finally: the authors have ensured that the research is compliant with a series of ethical norms established by different institutions, nationally and internationally – it is not their job here to resolve fundamental questions concerning the moral status of the human embryo. In the UK, whatever that status, the human embryo is the sort of thing that can be lawfully created and experimented on in the interests of the greater good (public interest).

Response: We thank the reviewer for their incredibly helpful and thorough review of the documents we provided. Their assessment is a useful and informed summary of the context in which our work was conducted.

General comments on the manuscript

There is a reassuring narrative in the manuscript, familiar from the previous work of Niakan with human embryos. The base editing technical aspects (sgRNA efficiency etc) are first addressed in human ES cells – which are also analysed to examine the role of NANOG in this context; mouse embryos are then used to test the effectiveness of this base editor in a mammalian embryo. Only when these components are completed satisfactorily are human embryos used to generate knowledge about human embryos (which cannot be guaranteed by using hES cells or mouse embryos). This is an example of the 'principle of subsidiarity', which requires that entities of a lower 'moral status' are used to answer scientific questions (where possible/feasible) before using entities of higher moral status (here, human embryos). Of course, this assumed hierarchy of moral status is contestable. Minimising the use of human embryos in this way is evidence of taking the special status of human embryos (in UK law) seriously, but it is also pragmatic: human embryos are not easy to source and are a rare, thus precious, commodity. Having said that, I'm not sure I would use the word 'precious' in the manuscript (l. 84, 132), since in addition to my use of it to highlight the limited numbers available, some might use it to denote an inherent moral status that is incompatible with research i.e. with the

instrumentalization of human embryos. Perhaps best to delete or use another word ('valuable'?) But I leave it to the authors to decide.

Response: We thank the reviewer for their incredibly helpful comments and summary of the context of the field. The reviewer makes a very important point about word choice when discussing human embryo research. We have amended the manuscript to replace the word precious with valuable. This is feedback which we will take on board for other contexts in which we discuss our research, thank you.

The comparative analysis of the human and mouse embryo phenotypes makes for an interesting read – and the species-specific roles of NANOG described are important, given that manipulating the fate of human pluripotent stem cells in regenerative medicine requires understanding human gene networks and cannot simply rely on knowledge from the mouse. It is also good to see that the speculation at the end of the manuscript about potential clinical applications (mutation correction) are tempered by the insistence that future clinical use is a decision for society and not just scientists, based on ethical inquiry, not just scientific feasibility.

Response: We thank the reviewer for their helpful comments. We used this as an opportunity to emphasize more the rigorous ethical inquiry and oversight that is needed together with extensive public discourse.

Specific comments/questions

I'm slightly confused about the blastocyst 4 data. The scRNAseq was performed on isolated (enriched) ICMs and the sequencing data suggested blasto 4 is effectively unedited/wild-type or het – but if I understand correctly, 57% of cells of this blasto show trophectoderm clustering. How efficient is the enrichment of ICM?

Response: We appreciate the reviewer's comment, which is also a point raised by Reviewer 2. ICM enrichment was performed using laser-assisted micodissection. This enriches for epiblast, primitive endoderm and polar trophectoderm for single-cell RNA sequencing and allows for the separation of the mural trophectoderm which is used for genotyping and karyotyping (Reviewer Figure 1a).

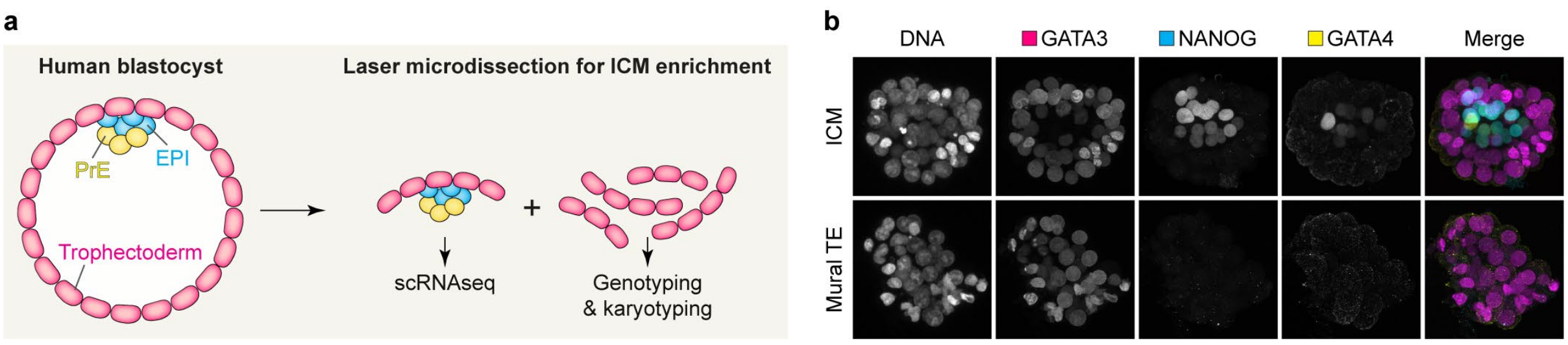

Reviewer Figure 1. Laser-assisted microdissection for ICM enrichment. a, Schematic representation of the experimental pipeline. b, Immunofluorescence of two parts of the blastocyst following laser-assisted microdissection.

The proportion of trophectoderm cells we see in this embryo is consistent with the proportions isolated in the Meistermann et al., Cell Stem Cell 2021, Petropolous et al.,

Cell 2016; and Simon et al., Nature Communications 2025 datasets, presented in new Figure 4d, which used similar laser-assisted ICM enrichment. The proportion of TE in the Blakeley et al., Development 2015 paper is lower but this looks to be an outlier in ICM enrichment compared to other studies. We thought to keep it in for transparency, but if the reviewer would so advise, we could remove the Blakeley et al 2015 example.

To address the question of efficiency of ICM enrichment, we repeated laser-assisted ICM enrichment on control blastocysts and performed immunofluorescence analysis and quantification of TE (GATA3), EPI (NANOG) and PrE (GATA4) associated molecular markers. We provide this data in new Extended data Fig. 6a (also shown in Reviewer Figure 1b above).

Moreover, in the revised manuscript we provide additional controls, replicated base editing of NANOG in additional human embryos and extensively revised the analysis, figures and manuscript based on feedback from all reviewers. This allowed us to clarify the genotype of the base edited embryos using a DNA Sanger sequencing-based genotyping on multiple trophoblast biopsies to identify *NANOG* null mutant embryos for subsequent analysis (new Figure 3b). We also provide immunofluorescence analysis of NANOG, GATA3 and GATA4 protein expression following NANOG base editing of human embryos (new Figure 2), which further corroborates the conclusions from our observations.

Why is NANOG expression reduced in NANOG KO hES cells? Nonsense-mediated decay?

Response: We thank the reviewer for highlighting this point. Yes, we speculate that *NANOG* transcripts are reduced in the NANOG KO hESCs by nonsense-mediated decay. Alternatively, differentiation of the hESCs triggered by destabilisation of pluripotency following *NANOG* perturbation could also lead to loss of *NANOG* transcripts. We have amended the manuscript to state: "*NANOG* transcript may be downregulated due to nonsense-mediated decay and/or due to differentiation of the human ESCs triggered by destabilisation of pluripotency following *NANOG* perturbation."

The focus on the impact of base editing on the intended target site (and bystander effects) is warranted, given previous problems with loss of heterozygosity etc. But was off-target base editing assessed as a possibility (as opposed to off-target cutting/repair)?

Response: The reviewer raises an excellent point about evaluating off-target editing. We have addressed this by assessing 10 in silico-predicted off-target sites, including a NANOG pseudogene, which was the top predicted off-target site (new Figure 3e and Extended Data Fig. 6e). We performed target amplicon next generation sequencing of a pool of 3 replicate biopsies from 10 NANOG base edited human blastocysts. Altogether we found lower than 0.15% read counts had any evidence of sequence variation from the reference in over 36,000 reads (new Extended Data Fig. 6e). This points to low rates of off-target base editing. We also complemented the low-pass whole genome sequencing-based karyotype analysis with G-band karyotype analysis which confirms absence of karyotypic abnormalities including evaluating genome wide structural changes (new Figure 3d and Extended Data Fig. 6d).

The number of human embryos analysed (transcriptomically) is small (4) and we are an outbred species with genetic variability between us. The upshot is a snapshot of NANOG function in the human embryo, rather than a definitive analysis – would the authors agree? Isn't this a limitation that should be briefly dealt with in the manuscript?

Response: The reviewer raises an important point about the limitations of the study. We have since increased the number of human embryos that were base edited and included *AAVS1* editing control datasets. This increased our n number from 4 blastocysts that were evaluated by single cell RNAseq to 13 blastocysts (11 NANOG base edited plus 2 AAVS1 controls (that anchor the data with previously published RNAseq at equivalent stages) (new Figure 3 and 4, new Extended Data Fig. 4f). We also base edited the *NANOG* locus in additional human embryos to evaluate the expression of NANOG, GATA4 and GATA3 proteins (new Figure 2). We hope the reviewer will agree that this is a more comprehensive analysis of the role of *NANOG* in a very challenging to study developmental context. The fact that we are able to replicate the data in a highly diverse genetic background from a variety of different donors gives us confidence in the conclusions.

Referee #2 (Remarks to the Author):

In this manuscript, Bower et al. employed ABE8e to edit the NANOG splicing site, thereby disrupting its function. Their findings elucidate NANOG's pivotal role in human embryonic lineage specification, revealing that human pluripotent cells require NANOG to sustain their identity—a requirement distinct from that observed in mouse embryonic stem cells. Notably, NANOG-edited human embryos retained endoderm cells despite the absence of the critical factor FGF4, with IGF1 mediating functional compensation. These unexpected results challenge existing paradigms and underscore the need for further investigation. The following points are suggested to revised the manuscript:

Major comments

1. In Ext. Data Fig. 1b, the efficiency of ABE8e alone appears higher than that of ABE8e with sg4. This raises the possibility of sgRNA-independent off-target activity, which should be examined. Similarly, in Ext. Data Fig. 1e, please clarify why editing the AAVS1 site in a single-cell clone resulted in an SNV in NANOG.

Response: The reviewer is pointing out the low levels of single nucleotide variants (SNVs) detected in the control samples following next generation on-target amplicon sequencing. The analysis pipeline we used does not discriminate whether there are polymerase-related PCR or technical sequencing-related errors and we provided the most raw and unfiltered datasets in the initial version of the figure to be as transparent as possible. We appreciate that this causes confusion and is not a standard way of presenting the data. We have since filltered out the reads with low rates of SNVs outside of the gRNA target region that are result of technical artifacts.

2. Following ABE8e editing of embryos, no RNA-level off-target analysis is presented. It is recommended to assess the events of RNA off-target editing.

Response: The reviewer raises an excellent point, as previous studies have reported spurious off-target RNA editing with cytidine deaminase base editor variants (e.g. Grunewald et al., Nature 2019). Notably, however, the ABE base editor have been shown to induce substantially lower levels of off-target RNA editing (Grunewald et al., Nature 2019; Sheriff et al., Scientific Reports 2022). To evaluate off-target RNA editing in our system, we analysed hESCs targeted with ABE8e and *NANOG* sgRNA (new Figure 3f). We have revised the manuscript on Page 10 to say: "to assess possible RNA off-target edits following ABE8e editing we transfected human ESCs and collected cells for genome-wide total RNAseq 48hrs following puromycin selection (Extended Data Fig. 2e). We applied a previously published pipeline to determine edits in RNAseq reads (Grunewald et al., Nature 2019) and observed A to G editing rate at all sites increased by 1.2% in ABE8e *NANOG*-edited cells compared to controls (n = 348 control, n = 415 ABE8e *NANOG*-targeted) (Fig. 3f). Notably, we observed similar percentages of base changes at other nucleotides not associated with adenine base editing conversion, for example 1.6% increase of C to G base conversion in ABE8e *NANOG*-targeted cells compared to controls (n = 91 control, n = 145 ABE8e *NANOG*-targeted), indicative of technical DNA polymerase or sequencing error rates."

3. The substantial differences in editing efficiency between Blastocyst 4 and Blastocysts 1–3 require explanation. Additionally, in Ext. Data Fig. 4a,b, the wild-type data should be presented separately from that of Blastocyst 4 for clarity.

Response: We thank the reviewer for this comment and agree that the previous version was not as clearly presented. We have simplified and expanded our analysis of editing efficiency using multiple trophectoderm biopsies from each embryo, and by adding more NANOG base edited human embryos to our analysis (now 359 NANOG-targeted cells from 11 embryos) plus AAVS1-targeted control (42 cells from 2 embryos) (new Figure 3b). We have significantly revised the figure by designating the genotype of the embryos using DNA Sanger sequencing-based genotyping of trophectoderm biopsies (new Figure 3b). This allowed us to distinguish homozygous-targeted embryos from mosaic/heterozygous-targeted embryos, and we used these categories in all subsequent analysis. We have updated Figures 3, 4 and 5 and Extended Data Figures accordingly.

4. In Ext. Data Fig. 5a,b, the authors used an spCas9 strategy in mouse zygotes to target the orthologous 3' region of Nanog exon 1. To ensure the reliability of downstream analyses, please provide DNA-level off-target data to confirm that the results are unaffected by unintended edits.

Response: We appreciate the reviewer's suggestion. We have designed PCR primers to amplify off-target sites in the mouse. We PCR amplified 9 *in silico* predicted off-target sites in pooled samples of 3 Nanog genome edited mouse embryos. This showed generally low rates of off-target edits except on one locus and we provide this data as bar charts in new Extended Data Figure 10. We also evaluated karyotype in mouse embryos

and found that in 1 out of 5 blastocysts we can detect segmental aneuploidy while 4 out of 5 mouse embryos were karyotypically normal. This data is presented in new Extended Data Fig 10. This is consistent with the approximately 20% aneuploidy we and others previously reported following the use of spCas9 enzyme in embryos (Alanis Lobato et al., 2023). Unfortunately, we were unable to design an sgRNA targeting a *Nanog* splice donor site in mouse embryos because no suitable PAM sequence for ABE8e is present at a distance compatible with efficient base editing of the splice donor site. As a result, ABE8e-driven base editing could not be applied to this locus in the mouse system.

5. Given that CRISPR/Cas9 mutagenesis can induce genomic instability, chromosomal rearrangements, and unintended on-target changes, could alternative editors—such as cytosine base editors or prime editors—be considered to introduce stop codons or deletions in Nanog in mouse zygotes, thus aligning more closely with the editing strategy used for NANOG disruption in human embryos?

Response: The reviewer raises an interesting point. We had considered using other base editors to target Nanog in mouse embryos and spent considerable time trying to design a working gRNA. Unfortunately, it was not possible to design a gRNA to target the region at a splice donor site using the ABE8e base editor, as explained in the previous comment.

Furthermore, we found that all forms of conventional -NGG PAM Cas9 activity at the mouse exon 1 splice donor were not viable, because of high divergence in sequence homology between human and mouse; whereas the human exon 1 splice donor site is rich with PAM compatible sequences, this is not the case in mouse. Indeed, the nearest -NGG sequence for the mouse exon 1 splice donor site motif is 55 bases away, whereas the human is an optimal 15 bases away.

We initially started the project with the idea to use spCas9 and switched to base editor in valuable human embryos only when we found in hESCs segmental aneuploidy of the on-target chromosome. We decided in the interest of time and resources to keep the mouse data because the findings in this species are less novel and there is extensive publications on the role of Nanog in the mouse, which our data confirmed (Allegre et al., 2022; Frankenberg et al., 2011; Chambers et al., 2003; Mitsui et al., 2003). We appreciate this is not ideal but hope that the reviewer will understand why we would prefer to keep this data.

We attempted prime editing with Esther Posfai (Yale) who published together with Britt Adamson (Yale) that this approach works in mouse embryos, especially when combined with an inhibitor of mismatch repair (Kim-Yip et al., Nature Biotechnology 2024). However, after evaluating pegRNAs targeting *NANOG* in HEK293 cells they found that the efficiency was low with approximately 5% of human cell lines having evidence of the intended edits (please see the peer reviewer figure below). The efficiency is too low to use this approach in human and mouse embryos.

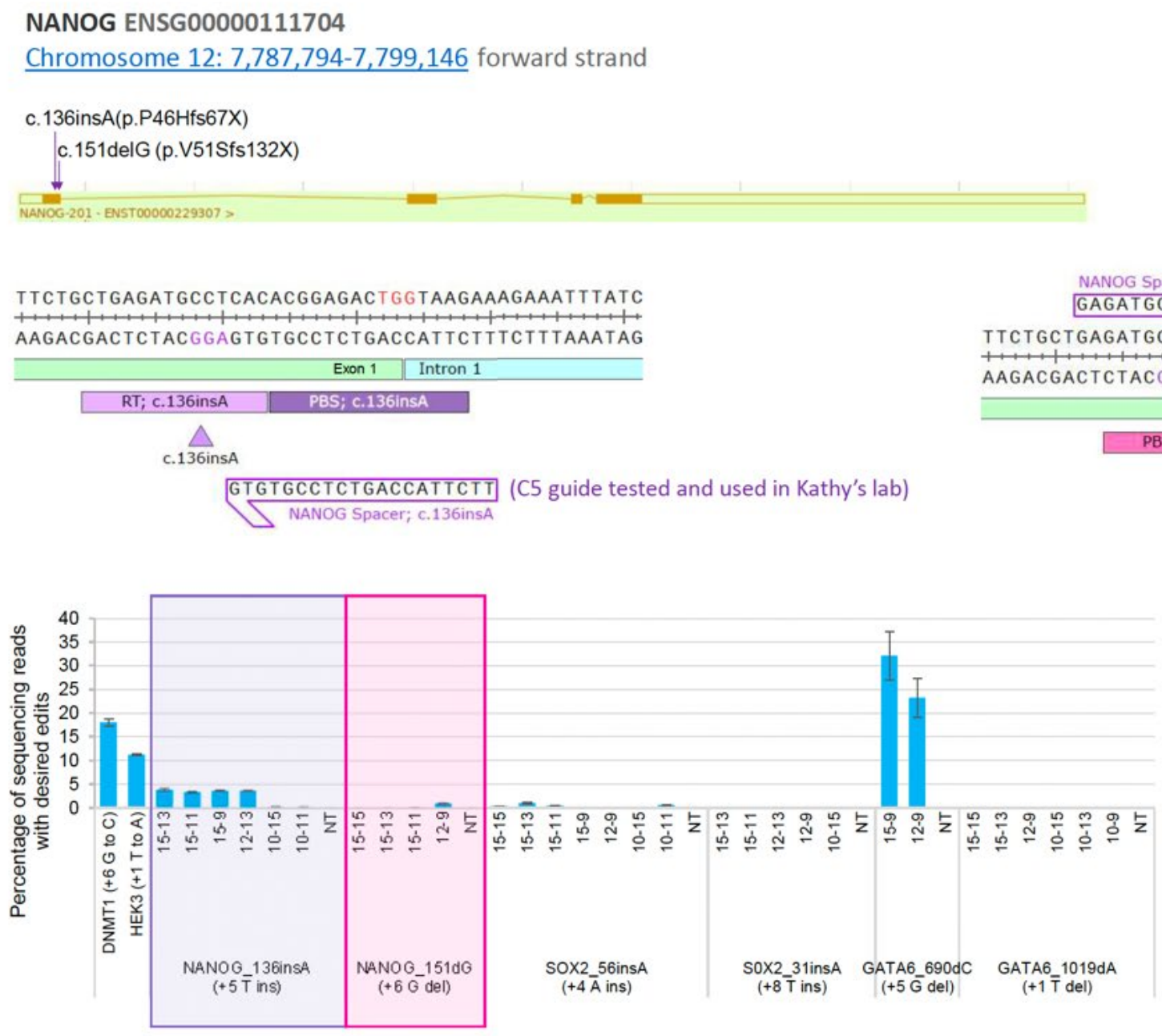

6. Beyond RTK signaling, could additional key pathways or alternative compensatory factors be identified?

Response: We amended the manuscript and updated Figure 5d and Extended Data Fig. 14 to expand on alternatives to IGF1 compensation. We also state:
"We previously showed crosstalk between FGF and IGF signalling in stem cell proliferation and that treatment with exogenous IGF1 led to increased numbers of ICM cells with embryos in a diapause-like state (Iyer et al., Cell 2024; Wamaitha et al., Nature Communications 2020), it would therefore be interesting to determine if FGF18, IGF1 or alternative RTK signalling components can compensate for the loss of *NANOG* and whether they may have a role in primitive endoderm specification in a non-perturbed context."

"In contrast to the mouse, we observed that *NANOG*-edited human embryos retained primitive endoderm cells despite the absence of FGF4 and our data suggests a number of plausible RTK signalling components that would be interesting to investigate in the future."

In terms of alternatives to RTK signalling, the gene set enrichment analysis did not identify an obvious alternative pathways. However, we provide all of the differentially expressed genes in Supplementary Table 2, which may be further analysed by others to identify candidate factors to functionally investigate in the future.

7. Following the detection of IGF1, please discuss its potential implications for future disease research or therapeutic applications.

Response: We thank the reviewer for this suggestion. As noted above we have removed the data on IGF1 because we were not able to repeat this. We have therefore not included reference of implications of IGF1 for future therapeutic applications given the change in emphasis.

Referee #3 (Remarks to the Author):

Summary

The authors present a novel application of adenine base editing (ABE8e) to knockout the transcription factor NANOG in human embryos, achieving this without detectable genotoxic effects. It is stated that the loss of NANOG disrupts the formation of the pluripotent epiblast, instead biasing cells toward yolk sac or placental-like lineages. Notably, the study shows evidence of a functional compensation mechanism in humans for primitive endoderm formation in the absence of NANOG, which is partially mediated by IGF1 signaling. These observations diverge from established mouse models, underscoring species-specific developmental pathways and reinforcing the critical importance of direct investigation in human embryos. The work also claims the potential of ABE8e's value for safe, targeted studies in human developmental biology.

Major comments

Although the use of base editing for functional KO studies in human embryos is original, a major issue when using gene editing in the human germline, remains the full efficiency of the technique, which requires analysis on different levels on a sufficient number of embryos. The ABE induces a splice donor site mutation (GT→GC) but does not show direct evidence of intron retention, exon skipping, or aberrant splicing in NANOG transcripts in embryos. Base editing at splice sites may not always abolish gene function; transcripts might still be produced with cryptic splicing. I suggest that the authors provide direct evidence of aberrant splicing (e.g., RT-PCR or NANOG-specific transcript reconstruction from scRNA-seq) to confirm splicing disruption and absence of functional transcripts. The authors have provided some evidence on the genomic and transcriptomic level for this, but show that a full knock-out is very hard to obtain in human embryos, with only 1 successful embryo (almost) fully edited. So all the next comparisons of transcription and species differences are biased by this low full KO editing efficiency, because of mosaic gene editing events which is a major flaw in this study. Furthermore, in the single-cell RNA-seq analysis, cells containing NANOG transcript reads were filtered out. However, it remains unclear how the authors confirmed that the retained cells were indeed fully edited, rather than unedited or TE cells. Additional clarification or validation is needed to ensure that the single-cell data analyzed truly reflect NANOG-deficient cells and not a mixture of cell types or editing states. It is unclear why the authors did not perform basic NANOG protein immunostaining in human embryos, which could have been readily combined with genomic-level editing verification. Such an approach would have provided direct evidence of functional protein loss and helped validate the claimed knockout phenotype. Given the already low editing efficiency, the scarcity of human oocytes available for research is understandable. However, the number of human embryos analyzed in this study is extremely limited and insufficient to robustly support the authors' conclusions.

Several key pieces of information are missing, which undermines the strength of the claims made (see further comments below). I strongly recommend that the authors expand their analysis to include a larger number of edited human embryos to allow for more statistically and biologically meaningful interpretations. Furthermore, the current data do not support the conclusion that ABE is highly efficient in this context, and I suggest that this statement be moderated accordingly. In addition, appropriate controls in human embryos are lacking. Even in the mouse embryo experiments, the number of embryos analyzed is very limited, raising concerns about the reproducibility and generalizability of the findings. Additional biological replicates and inclusion of proper negative and positive controls are needed to strengthen the conclusions drawn from both human and mouse data.

Response: The reviewer makes several excellent points. To address their concerns, we performed the following:

1. To address the point about lack of evidence of splicing defects leading to null mutations we provide:
    a. Examples of reconstructed RNAseq tracks using IGV browser screenshots in Extended Data Fig. 8 that show transcriptional reads into the intronic region, which are absent in control *AAVS1*-targeted cells, as expected. This would be expected to abolish functional protein because transcription into the intron would lead to introduction of amino acids or premature termination codons that would affect protein function.

    b. In addition, we designed PCR primers at exonic regions across the exon 1 splice site. We PCR amplified cDNA purified from AAVS1 targeted versus NANOG targeted embryos (Extended Data Fig. 7). While we could amplify a product across exons 1 and 2 and exons 1 and 4 in the cDNA from AAVS1 targeted embryos, the NANOG targeted embryos were not able to be amplified at these regions, presumably because of intron retention and the product being too large to amplify or due to loss of cDNA from non-sense mediate decay in some samples. We confirmed that there was cDNA purified in all samples by amplifying a control *GAPDH* site where a band was present in all samples. Again, this points to appropriate splicing in AAVS1 targeted samples and a splicing defect in NANOG targeted samples.

2. To address the concern about lack of controls, we edited the *AAVS1* locus and collected single cells for RNAseq analysis to anchor to the previously published datasets and provide reassurance that we are able to collect cells across TE, PrE, EPI and ICM lineages. This data in presented in new Figures 3 and 4.
3. To address the reviewer's concerns regarding sample size, mosaicism, and editing efficiency, we undertook the following approaches:
    a. <u>Reduction of mosaicism through optimisation of ABE8e protein delivery.</u> We purified recombinant ABE8e protein (new Extended Data Fig. 5a) after extensively optimising its production and use over a four-year period to ensure robust activity without inducing toxicity in human embryos. Delivery

of ABE8e as a purified protein reduced mosaicism and facilitated the generation of homozygous base-edited embryos. Editing outcomes were assessed by DNA Sanger sequencing-based genotyping of multiple independent trophectoderm biopsies from individual embryos, confirming reduced mosaicism and the presence of homozygous edits (new Figure 3b).

b. Increased sample size for scRNA sequencing and quantitative assessment of editing efficiency. In response to the reviewer's request to increase *n*, we performed single-cell RNA sequencing on an additional seven *NANOG* base-edited human embryos. Editing reagents were delivered as ribonucleoprotein complexes and microinjected coincident with ICSI. Embryos that reached blastocyst stage were laser dissected, and an additional 202 single cells were collected and sequenced, increasing the total number of single cells analysed to 359. All *NANOG* base-edited embryos that reached the blastocyst stage (12/12) showed evidence of successful editing (new Figure 3b), exhibiting either mosaic/heterozygous editing or homozygous null mutations at the *NANOG* locus. When combined with embryos previously edited using ABE8e delivered as mRNA together with sgRNA, this yielded an overall efficiency of 25% (3 out of 12 blastocysts) for generating complete *NANOG* homozygous null mutant embryos (new Figure 3b, embryos #4, #7, and #8 are null mutants).

c. Inclusion of arrested embryos to maximise assessment of editing efficiency. To maximise the information obtained from the limited number of human embryos available for analysis and to account for the challenging experimental context, we additionally analysed 16 *NANOG*-targeted embryos that arrested prior to blastocyst formation. Of these, 62.5% (10/16) embryos showed clear evidence of base editing (new Extended Data Fig. 5b-d), indicating high editing activity even in embryos that failed to progress developmentally. Notably, many embryos that lacked detectable editing arrested at the two-cell stage, at which point the base edit is unlikely to have been incorporated into the genome due to limited number of DNA replication events.

d. Restriction of single-cell analyses to genetically confirmed *NANOG* null embryos. To ensure that the single-cell RNA-seq analyses reflect bona fide *NANOG*-deficient cells, rather than a mixture of editing states or mosaic genotypes, we focused our downstream analyses on *NANOG* homozygous null mutant embryos (new Figure 3b). The *NANOG* null mutant embryos were compared against *AAVS1*-edited control embryos and previously published control datasets. We have updated the differential gene expression analyses, as well as the dot plot and violin plot comparisons, accordingly. These revised analyses are presented in new Figures 3, 4, and 5, as well as the associated new Extended Data Figures. Importantly, this refined analysis demonstrated that *NANOG* null mutant embryos give rise to primitive endoderm and trophoblast lineages, while completely lacking

epiblast cells, consistent with the conclusions drawn from our original analysis.

4. To confirm loss of NANOG protein, we performed NANOG base editing (using RNP complexes) in additional human embryos followed by immunofluorescence analysis of NANOG, GATA4, and GATA3 expression, which mark epiblast, primitive endoderm, and trophoblast lineages, respectively. Quantification of protein expression is provided in new Figure 2 and demonstrates a clear loss of NANOG protein with preservation of GATA4 and GATA3 expression. Notably, 3 out of 7 *NANOG*-targeted human embryos (42.85%) exhibited a complete absence of detectable NANOG protein while retaining GATA4 and GATA3 expression, a phenotype that was not observed in control embryos. Taken together, this experiment demonstrates that our base editing strategy achieves high-efficiency disruption of NANOG protein expression in a substantial fraction of embryos, and that primitive endoderm and trophoblast lineages can form despite complete loss of NANOG function, as evidenced by retention of GATA4 and GATA3 expression.
5. Given that the mouse experiments have been extensively characterised by others, namely Claire Chazaud shows by qPCR analysis differentially expressed genes consistent with our RNAseq data (Frankenberg et al., Developmental Cell 2011; Allegre et al., Nature Communications 2022). Moreover, the Chazaud laboratory have extensively characterised by protein and Fluidigm qPCR analysis defects in primitive endoderm specification in the *Nanog* null mutant mouse embryo using classical genetic breeding. Our data is consistent with their results where we also observe a loss of PrE markers such as Gata4 and Sox17. We integrated our control and *Nanog* genome edited mouse scRNAseq data with a previously published atlas of mouse *in vivo* flushed embryos at embryonic days 3.5 and 4.5 from Kat Hadjantonakis' and Dana Pe'er's laboratories (Nowotschin et al., Nature 2019). This demonstrates that our *Aavs1* control edited mouse (n = 62) embryo cells cluster with reference TE, PrE and EPI groups, whereas the *Nanog* genome edited mouse (n = 64) embryo cells cluster with the TE and ICM. We also perform additional immunofluorescence analysis of Nanog genome edited versus Aavs1 edited mouse embryos which confirms the same conclusions about absence of both EPI and PrE and present this in Extended Data Fig. 9. Given that our data confirms and further expands previously published Fluidigm and protein expression data, we felt that it was not necessary to generate additional scRNAseq data in the mouse. This was also cost prohibitive and we prioritised sequencing additional human embryo datasets.

Secondly, the manuscript lacks critical information regarding the accuracy of the microdissection used to separate the two (or three) lineages of the human blastocyst. This is a technically challenging procedure that relies on clear identification of the ICM, which is not always visually discernible. In Figure 2a, it appears that laser dissection was used to isolate only a portion of the blastocyst, likely containing a mixture of cell types, including TE cells, rather than specifically isolating distinct lineages. This raises concerns about the purity of the dissected populations. Given the low editing efficiency and the likelihood of mosaicism, how do the authors distinguish transcriptional effects of NANOG editing from those arising due to TE contamination? Is lineage assignment

inferred solely based on the absence or reduced number of NANOG transcript reads? If so, this is not a reliable indicator of editing success or lineage identity. Additional clarification on the lineage identification strategy, preferably supported by lineage-specific markers or single-cell-level annotation, is needed to validate these interpretations.

Response: The reviewer raises a good point. We know of no method to cleanly remove TE. We and others have previously performed immunosurgery, which works very well to isolate ICM from the mouse but leaves significant numbers of TE cells in human embryos treated with equivalent methods. We and others have also used laser-mediated ICM isolation (Meistermann et al., Cell Stem Cell 2021; Simon et al., Nature Comms 2025; Blakeley et al., Development 2015; Hyslop et al., Nature 2026; Fogarty et al., Nature 2017). This also leaves residual polar TE cells. The proportion of TE cells is approximately 50% from single cell RNAseq based lineage assignments using molecular markers. No group has performed IF to date of the ICM enriched portion versus mural TE following either laser dissection or immunosurgery to determine the enrichment of TE, EPI or PrE by protein expression. Here we perform this analysis for the first time on laser dissected ICM versus mural TE. We present this data in new Extended Data Fig. 6a.

Additional clarification on the lineage identification strategy, preferably supported by lineage-specific markers or single-cell-level annotation, is needed to validate these interpretations.

Response: We provide heatmap expression of individual cells and dotplots of multiple lineage-associated genes to support the lineage identification in both the human and mouse scRNAseq datasets. In the human datasets, cells from each of the lineages clustered separately and the trophectoderm was distinguished by molecular markers *GATA2*, *GATA3*, *CDX2*, the inner cell mass expressed *GATA6* and *POU5F1*, the epiblast was enriched for *NANOG*, *POU5F1* and *ARGFX*, while the primitive endoderm expressed *GATA6*, *GATA4* and *PDGFRA*. These data are presented in Figure 4b,e. We also provide a count matrix table (Supplementary Table 5) annotating the lineage associated identity of each individual cell.

In the mouse, the cell types were initially distinguished by the expression of trophectoderm markers Cdx2 and Gata3, epiblast by the expression of Nanog and Sox2 and primitive endoderm by the expression of Gata6 and Sox17 (Extended Data Fig. 8d). We subsequently confirmed these assignments using dot plots for additional markers presented in Extended Data Fig. 8e. We have used similar strategies to assign lineages in both human and mouse in past publications (Blakeley et al., Development 2015) and our strategy for lineage assignment was confirmed by other studies (e.g. Stirparo et al., Development 2018).

Finally, the mechanistic experiment involving IGF1 treatment was conducted on a very small number of embryos, which limits the ability to draw robust conclusions. The observed effects should be interpreted with caution, and the authors should either increase the number of biological replicates or clearly acknowledge the limitations of these preliminary findings.

Response: We thank the reviewer for this insightful comment. We agree that the IGF1 treatment experiment was performed on a limited number of embryos, which restricts the strength of any mechanistic conclusions that can be drawn. In revising the manuscript, we prioritised the use of the valuable human embryos for experiments that were most critical to strengthening the central conclusions of the study, including increasing the single-cell RNA-seq sample size and independently validating NANOG protein loss and lineage outcomes by immunofluorescence. These additional experiments substantially reinforce our main conclusion that NANOG null mutant embryos retain primitive endoderm and trophoblast lineages while lacking epiblast cells.

Given the limited number of human embryos available, we were not able to expand the IGF1 treatment experiment to a sufficiently powered sample size. We have therefore removed the IGF1 data from the revised manuscript. Instead, we now provide a more general discussion of candidate signalling pathways and factors that may contribute to the unexpected persistence of primitive endoderm specification in the absence of NANOG (new Extended Data Fig. 14), thereby outlining important directions for future investigation.

Specific comments

- Regarding the sequencing depth (scRNAseq) of the blastocysts: While the authors report average read counts per cell at the NANOG locus, this coverage is likely insufficient for robust base-level genotyping in single cells. Genotype assignments (WT vs edited vs unclear vs absent) should be accompanied by read count thresholds to increase confidence, particularly in cells where expression is low due to the expected functional knockout.

Response: We appreciate the reviewer's point about assigning genotype to targeted embryos. We have since revised our approach to assign genotype based on genomic DNA Sanger sequencing-based genotyping of multiple biopsies from targeted embryos. We provide the genotyping assignments data in the new Figure 3b.

- Fig 2 (following previous comment): The substantial mosaicism observed within TE (Fig. 2e) raises the possibility that similar mosaicism exists within the ICM. This could affect the interpretation of lineage specification defects as being solely due to complete loss of NANOG. Additional analysis of mosaicism in ICM cells would strengthen the conclusions.

Response: We switched to using stringent DNA-based genotyping of multiple biopsies to assign homozygous mutant embryos. *NANOG* reads from scRNAseq analysis of these embryos either had clear evidence of targeting leading to splicing errors, were absent, or did not span the target region; overall none of the cells from these embryos had evidence of wildtype alleles or reads in their transcriptome. This gives us confidence that the embryos are *NANOG* null mutant. We distinguished these NANOG null mutant embryos from mosaic/heterozygously edited embryos which had evidence of wildtype transcripts and genotypes based on scRNAseq and DNA-based Sanger sequencing. Comparisons

of differential gene expression and lineage markers were amended to focus on *NANOG* null mutant embryos versus *AAVS1* and previously published control datasets.

- Fig 2: Why did the authors decide to not perform scRNAseq of blastocyst 5, since it carries a more homozygous genotype that could therefore strengthen the findings of the study?

Response: Unfortunately, the ICM sample was lysed during processing of single cell disaggregation before picking and therefore we could not perform this analysis. We had the trophectoderm biopsies which we decided to retain to indicate the rate of NANOG base editing in developing embryos. We have since edited a number of additional human embryos which we provide in new Figures 2-5.

- Fig 2: The authors should also include the whole-genome sequencing analysis of blastocyst 3, given that this is the only homozygous mutant.

Response: We thank the reviewer for this suggestion. In the updated manuscript, this embryo is designated as blastocyst #4, and the whole-genome sequencing analysis is presented in the new Figure 3d.

- The study showed a reduction in OCT4 protein in NANOG-/- hESCs, but not in their transcriptional data. Could the authors elaborate on this in the discussion?

Response: We thank the reviewer for this comment, similarly identified by Reviewer 1. We do identify a decrease in *POU5F1* expression in the bulk RNAseq though this is not significant (Supplementary Table 1). We assume this is due to variable rates of *NANOG* editing and rate of differentiation across the cell lines at the time point collected.

- What are the scores of human embryos and more specifically the ICM vs TE in the human embryo experiments? As timelapse imaging was performed, this should be very feasible. From the pictures, you only see an obvious ICM in blastocyst

Response: Embryos were graded by two clinical embryologists and the data is provided in the new Figure 3b.

- Can the authors clarify/extend the mention of a 'diapause like state' upon IGF1 treatment? Do treated embryos exhibit decreased proliferation?

Response: As noted above we have since removed the emphasis on IGF1 and instead provide alternative factors that may functionally compensate for the loss of FGF4 in primitive endoderm specification. We have therefore removed the mention of a diapause like state upon IGF1 treatment.

- Extended Data Figure 4: Genotyping NANOG-targeted human embryos at the blastocyst stage: why only data for 3 embryos?

Response: For our initial submission, we had a total of 5 edited embryos; 1 of which had low rates of editing, 4 of which had high rates of editing; of these 4, 1 embryo was unfortunately lysed during ICM disaggregation and therefore was not processed by scRNAseq analysis. We have since increased our total number of *NANOG* targeted embryos to n = 11 in total, with genotypes shown in new Figure 3b.

- Extended Data Figure 9: Differences and similarities in expression of receptor tyrosine kinase (RTK) signalling components and primitive endoderm-associated genes in human versus mouse NANOG/Nanog-edited embryos. Also including not fully edited mouse/human embryos?

Response: We focused on the *NANOG/Nanog* loss-of-function compared to controls and did not provide the heterozygous/mosaic embryos because we wanted to keep the focus of the results on drawing biological insights from unambiguous genotypes. We also were limited in the space for figures and text. Should the reviewer like us to include this data, we would be happy to amend the figures and text and would need to ask the journal editor for permission to increase the number of figures and text.

Referee #4 (Remarks to the Author):

This study by Bower et al. investigates the role of the pluripotency-associated transcription factor NANOG in early human embryos using adenine base editor ABE8e, a next-generation genome-editing tool that avoids DNA double-strand breaks and the associated risks of genotoxicity and aneuploidy inherent to conventional CRISPR/Cas9 approaches. To the best of my knowledge, this represents the first successful application of base editing for functional interrogation of gene activity in human embryos. By precisely converting the canonical "GT" splice donor site in exon 1 of NANOG to "GC," the authors efficiently achieved a loss-of-function mutation. After validating this editing strategy in human embryonic stem cells (ESCs), they applied it to human MII-stage oocytes by co-injection with sperms and cultured them to the blastocyst stage. Genotyping confirmed efficient and accurate base editing, with mutant embryos progressing morphologically to the blastocyst stage. However, single-cell transcriptomic analysis revealed a profound disruption in epiblast formation: the NANOG-edited embryos failed to establish a pluripotent epiblast and instead upregulated gene programs characteristic of the trophectoderm and hypoblast lineages. These results clearly demonstrate that NANOG is indispensable for human epiblast fate acquisition and pluripotency maintenance. Importantly, the findings also highlight key species-specific regulatory differences. Unlike mouse Nanog mutants—which fail to specify either epiblast or primitive endoderm—NANOG-deficient human embryos still gave rise to hypoblast-like cells, possibly due to compensatory IGF signaling that circumvents the requirement for NANOG-induced FGF4. Altogether, this study underscores the necessity of studying human development directly in human embryos or primate models, as rodent systems do not fully capture the nuances of human pluripotency regulation. It also provides valuable insight into the molecular mechanisms that govern early lineage segregation and has broader implications for understanding human infertility, improving stem cell models, and evaluating the safety of germline genome editing. That said, despite the significance of the findings, the current study does

not sufficiently assess the genomic and epigenetic safety profile of ABE8e in human embryos. As it stands, these safety concerns, along with other major concerns, preclude a recommendation for publication in Nature in its current form.

Major concerns:

1. The manuscript emphasizes the relative safety of adenine base editing (ABE) compared to CRISPR/Cas9-mediated editing, however, this claim is not adequately supported by evidence. Given the potential translational implications of applying ABE for germline gene correction—as noted by the authors in the discussion section ("our approach also establishes a framework for disease modeling and potential therapeutic correction through base editing")—it is critical to rigorously evaluate both the safety and specificity of this approach in human embryos. At present, the manuscript lacks comprehensive off-target analyses and broader assessments of potential genotoxicity, transcriptomic perturbation, or epigenetic dysregulation. To strengthen the study's impact and support its potential translational relevance, the authors are encouraged to incorporate the following safety-related experiments and analyses in a revised version:

Response : We do not advocate for the use of this technology until there is more extensive evaluation of safety and efficacy and until there is wide-spread societal debate. We also make it clear that further evaluation of safety is needed before consideration of clinical use. In the final paragraph of the manuscript we note: "Nonetheless, clinical translation of genome editing in human embryos must not proceed unless there is broad societal debate and support. Caution, underpinned by rigorous ethical inquiry and oversight together with extensive public discourse, are needed. Moreover, while we did not observe obvious DNA or RNA off-target effects or chromosome gains or losses, it will be essential to further evaluate the possibility of genotoxicity in a larger cohort and across more genomic loci prior to consideration for clinical use in heritable genome editing."

We wanted to emphasize that the focus of our manuscript is on drawing biological insight into the role of a developmental regulator NANOG and providing a proof-of-principle of the utility of base editors for molecular genetic analysis of human embryogenesis. We are aware of another manuscript being reviewed at the same time which has a focus on clinical translation. The bar for use in basic research studies is different to that for clinical translation. Nevertheless, we attempted to address as many of the comments as we could reasonably perform with the resources available to us. We significantly increased the number of NANOG edited human embryos, provided additional controls and reference data comparisons. We evaluated RNA off-target editing and performed G-band karyotype analysis. We retargeted stem cell lines and revised much of the figures to address the reviewers points. We also performed protein expression analysis which independently confirms conclusions drawn from single-cell RNAseq analysis. We hope the reviewer agrees that we have addressed their most important points and have improved the study.

a) To support the safety profile of ABE8e in human embryos, the authors should perform high-coverage whole-genome sequencing of edited embryos to detect off-target base edits, indels, or structural variants beyond NANOG. Prior studies in hESCs (PMID:

35991312) found no detectable off-targets using similar methods. Special attention should be given to noncoding regulatory regions (e.g., promoters, enhancers, UTRs) that could impact gene expression and viability. Given that NANOG-null embryos may not yield ESCs, the authors could instead derive trophoblast stem cells (TSCs) from trophectoderm biopsies as a surrogate platform for comprehensive genomic and transcriptomic profiling. If concerns arise about genomic stability during extended hTSC culture, analyses could focus on early-passage cells or blastocyst outgrowths. For a broader safety assessment of ABE8e, editing a neutral locus such as AAVS1 followed by ESC derivation and comprehensive characterization of edited lines, including off-target analysis, transcriptomic profiling, and epigenetic evaluation, could be considered.

Response: We appreciate the reviewer's point and agree that additional data is needed to determine off-target effects, if any. To address this point we performed the following:

1. We retargeted the *NANOG* locus in human ESCs by base editing followed by clinical-grade G-band karyotype analysis which we present in Extended Data Fig. 3c showing normal karyotypes.
2. We performed target amplicon sequencing of 10 *in silico* predicted off-target loci in 10 NANOG targeted human embryos and 30 biopsies, which we present in Fig. 3d and Extended Data Fig 6 showing 0.15% or less edits.
3. Further karyotype analysis of either whole embryos following base editing or biopsy of trophectoderm samples did not identify the types of whole chromosome or segmental aneuploidies we readily detected in the past using the spCas9 variant.
4. Our attempts to derive human trophoblast stem cells from NANOG targeted embryos failed n = 6 embryos (please see the peer reviewer figure below), consistent with recent reports of low efficiency from Marta Shahbazi's lab (LMB) (Schwarz et al., bioRxiv 2025). We unfortunately do not have sufficient human embryos to repeat this attempt.

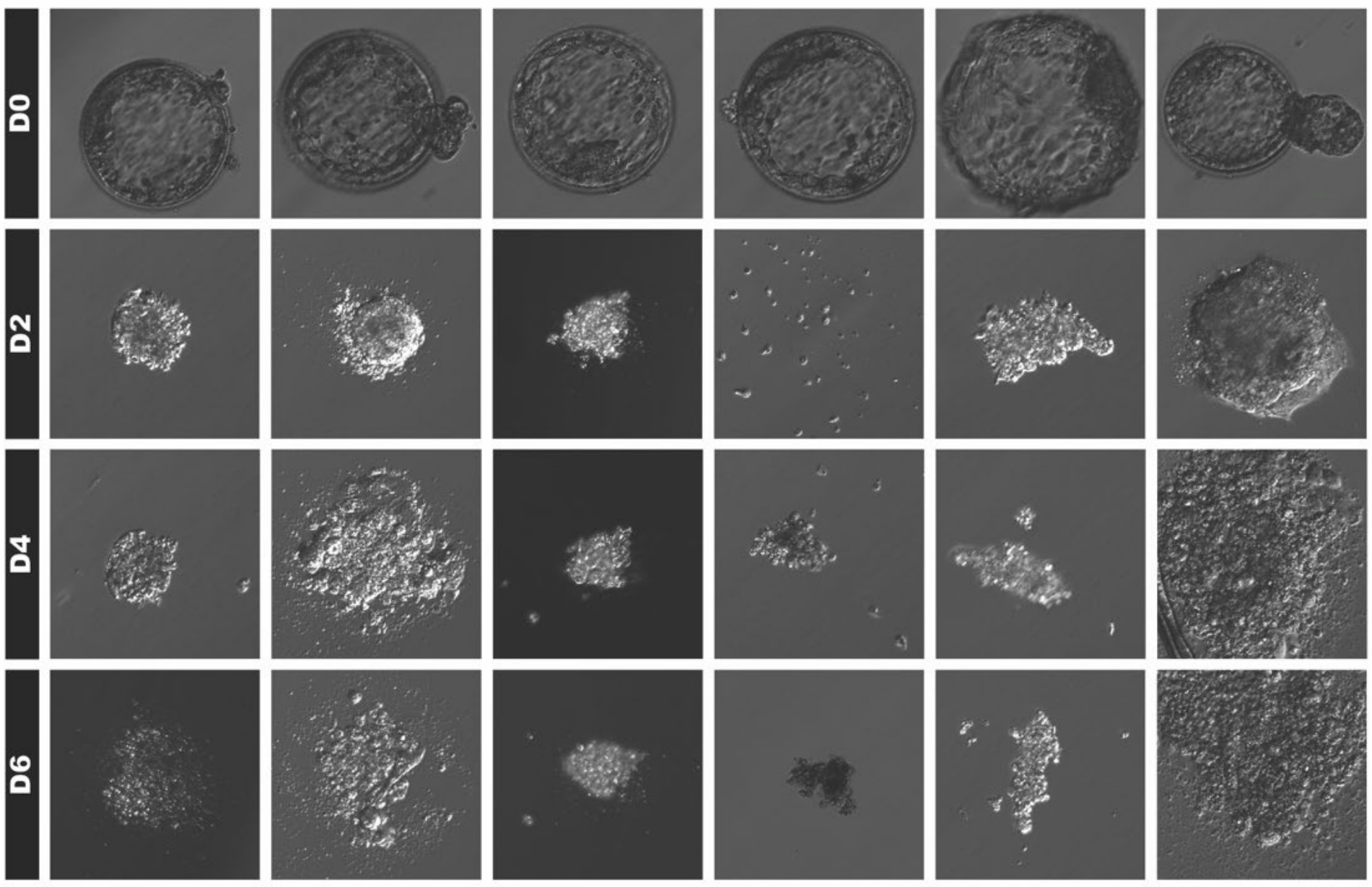

As noted above our study is focused on understanding the role of a developmental regulator and not on clinical translation. Therefore, we feel that the bar for safety is different. We are aware of another manuscript currently under review that more directly

addresses the concerns about safety following the derivation of ESCs. We also wanted to note that we have limited availability of gametes and embryos donated to research and undergo strict regulatory oversight. We therefore need to be cautious about the proportionate use of human embryos to address meaningful conclusions of direct relevance to the study. We therefore are not able to provide additional experiments to further address this point.

We provide the additional note in the last paragraph that further evaluate of the possibility of genotoxicity in a larger cohort and across more genomic loci prior to consideration for clinical use is needed. We hope the reviewer will agree that this is a proportionate use of valuable human embryos given the focus is on understanding the role of NANOG in early development.

b) While WGS of trophectoderm biopsies (Fig. 2f) assessed aneuploidy and chromosomal loss, these analyses are insufficient to rule out broader genomic instability. Despite avoiding double-strand breaks, ABE8e has been linked to large-scale structural variants—including deletions, duplications, and translocations—in mammalian embryos (PMID: 39529170). To rigorously evaluate genome integrity, the authors should apply structural variant detection pipelines to existing WGS data or perform long-read sequencing to capture complex rearrangements. Even rare events can disrupt cell function, induce apoptosis, or cause mosaicism, posing risks to embryo development. A comprehensive genome-wide analysis is needed to validate editing safety.

If you don't see aneuploidy it is unlikely to see del, duplication, translocation

Response: To address this, we performed 3 rounds of NANOG/ABE8e targeting in human ESCs and subjected them for karyotype analysis with Cell Guidance Systems, a company that assesses cell cultures with clinically qualified cytogeneticists. Karyotyping in this manner is widely used by regulatory agencies and scientific journals to assess cell genetic integrity. The service uses 20 cells assayed, which should reveal mosaicism rates of >5% in cultures. We found across 3 repeats no incidents of abnormal karyotype after NANOG ABE8e editing. As noted above we also make the point that "while we did not observe obvious DNA or RNA off-target effects or chromosome gains or losses, it will be essential to further evaluate the possibility of genotoxicity in a larger cohort and across more genomic loci prior to consideration for clinical use in heritable genome editing."

Notably we did not observe activation of TP53 or factors involved in apoptosis pathways from the RNAseq analysis of human ESCs or embryos, in contrast to that reported by Wu et al., Genome Biology 2024, which we expand on in the response below.

c) While the manuscript documents lineage shifts in NANOG-edited embryos, a more comprehensive transcriptomic analysis is warranted. Specifically, ICM-derived cells that adopt trophectoderm-like and hypoblast-like fates should be directly compared to bona fide TE and hypoblast cells from control embryos. Differential gene expression and pathway enrichment analyses could help reveal whether the converted cells retain residual pluripotency signatures or activate aberrant programs, such as DNA damage

response, apoptosis, or cell cycle dysregulation—pathways previously linked to ABE-induced aneuploidy (PMID: 39529170). Additionally, integrating these transcriptomic findings with genomic variant data would clarify whether any observed expression changes correlate with potential off-target edits or unintended structural variants.

Response: We have significantly expanded our analysis by including additional NANOG base edited human embryos to our analysis (now 359 NANOG-targeted cells from 11 embryos) plus AAVS1-targeted control (42 cells from 2 embryos). We have updated Figures 3, 4 and 5 and Extended Data Figures accordingly. We also provide additional volcano plots and gene enrichment terms in Extended Data Figure 13 comparing all cells, all ICM cells or all TE cells. We also provide a Supplementary Table 4 of gene enrichment terms related to this analysis. This provides further evidence that the NANOG-null mutant human and mouse embryo cells downregulate factors associated with the pluripotent epiblast and embryonic stem cells and upregulate factors associated with the trophectoderm compared to AAVS1 control edited human and mouse embryos. The comparisons between ICM cells or between TE cells are not as statistically significant given the low n number. We therefore focused on highlighting the enrichment terms in the all cell comparisons.

Wu et al., Genome Biology 2024 reported transcriptional differences in apoptosis, cell cycle and DNA damage response factors in primary human T cells that were edited with adenine base editors compared to controls (e.g. Figure 3 PMID: 39529170). We provide Supplementary Tables 1, 2 and 3 indicating that there is a lack of significant upregulation of the transcripts reported by Wu et al., Genome Biology 2024 in our NANOG/Nanog edited embryos or human ESCs. For example, there is no significant upregulation of *TP53*, *JUN*, *DNAJB11*, *CKS2*, *UBE2C*, *TNFSF10*, etc and we do not observe an enrichment of gene ontology terms associated with TP53 pathway, cell death or necrosis that the Wu et al., Genome Biology 2024 group reported in primary T cells.

d) The deaminase in ABE can induce unintended A→G edits in RNA independent of gRNAs. Although transient, such edits may disrupt gene expression. Comparing RNA-seq reads to the reference genome can help detect and quantify RNA off-target effects of ABE.

Response: The reviewer raises an excellent point, which was also raised by Reviewer 2. Previous studies have reported spurious off-target RNA editing with cytidine deaminase base editor variants (e.g. Grunewald et al., Nature 2019). Notably, however, the ABE base editor have been shown to induce substantially lower levels of off-target RNA editing (Grunewald et al., Nature 2019; Sheriff et al., Scientific Reports 2022). To evaluate off-target RNA editing in our system, we analysed hESCs targeted with ABE8e and *NANOG* sgRNA (new Figure 3f). We have revised the manuscript on Page 10 to say: "to assess possible RNA off-target edits following ABE8e editing we transfected human ESCs and collected cells for genome-wide total RNAseq 48hrs following puromycin selection (Extended Data Fig. 2e). We applied a previously published pipeline to determine edits in RNAseq reads (Grunewald et al., Nature 2019) and observed A to G editing rate at all sites increased by 1.2% in ABE8e *NANOG*-edited cells compared to controls (n = 348 control, n = 415 ABE8e *NANOG*-targeted) (Fig. 3f). Notably, we observed similar

percentages of base changes at other nucleotides not associated with adenine base editing conversion, for example 1.6% increase of C to G base conversion in ABE8e *NANOG*-targeted cells compared to controls (n = 91 control, n = 145 ABE8e *NANOG*-targeted), indicative of technical DNA polymerase or sequencing error rates.”

2. The authors attribute the divergent effects of NANOG loss in mouse and human ESCs to species differences. However, mouse ESCs represent the naïve state, while human ESCs resemble post-implantation, primed epiblasts. To rule out pluripotency-state effects, the authors should evaluate NANOG deficiency in mouse epiblast stem cells and naïve-state human ESCs.

Response: The reviewer raises a good point. We attempted to generate *NANOG* null mutant naïve hESCs and despite several attempts were unsuccessful (Extended Data Fig. 2). As a comparison we targeted AAVS1 in parallel and observed moderate and persistent base editing. We believe that transfection of naïve hESCs is not very efficient. Moreover, we believe there may be an essential role for NANOG in naïve hESCs that precludes our ability efficiently collect enough edited cells for subsequent analysis because by contrast we were able to expand AAVS1 edited cells. This is in contrast to primed hESCs where transfection is more efficient and we observed high rates of NANOG base editing. We also attempted electroporation in naïve hESCs but this was also insufficient. We provide these results in Extended Data Fig. 2.
We did not target mouse epiblast stem cells because this was not the focus of the paper and has been reported by others, notably Sun et al Developmental Biology 2014. The functional role of *Nanog* in mESCs has been well characterised by others. We accordingly revised our manuscript to state:

“The phenotype we observed in human ESCs contrasts with *Nanog*-null mouse ESCs which remain pluripotent, undergo indefinite self-renewal, express pluripotency-associated factors *Oct4, Sox2, Dppa3, Klf4, Esrrb* and *Zfp42*, and chimerically contribute to all somatic lineages and the germ-line (Chambers et al., Nature 2007; Zhang et al., Cell Reports 2018), which may reflect stem cell state, culture condition or species differences. The role of *NANOG* in human ESCs is reminiscent of that reported in primed mouse EpiSCs, where it functions to inhibit somatic cell lineages, including neuroectoderm differentiation (Sun et al., Developmental Biology 2014), consistent with previous studies of human ESCs in equivalent primed culture conditions (Vallier et al., Development 2009).”

3. The conclusions of altered lineage specification in NANOG-edited embryos rely heavily on scRNA-seq clustering (e.g., Fig. 3) without independent validation. Immunostaining for epiblast, hypoblast/primitive endoderm, and trophectoderm markers in control and NANOG-edited embryos, along with quantification of lineage proportions, is needed to confirm disrupted specification.

Response: This is an excellent suggestion. We performed immunofluorescence analysis of NANOG, GATA4 and GATA3 in NANOG base edited embryos. Co-staining and quantification of placental-progenitor (trophectoderm) and yolk sac progenitor (primitive endoderm) markers GATA3 and GATA4 showed the retention of these extraembryonic

lineages despite the loss of *NANOG* (Fig. 2e-j). We performed quantification of the proportion of cells expressing these lineage-associated molecular markers compared to expression in control embryos. This data in presented in an updated Figure 2 and substantiate the conclusions from single cell RNAseq analysis.

4. It remains unclear how NANOG-deficient ICM cells adopt trophectoderm-like and hypoblast-like fates. The authors could explore NANOG knockout in naïve hESCs to gain mechanistic insight into these lineage conversions. Does knocking out NANOG in naïve hESCs also drive the cells to TE-like and hypoblast-like lineages?

Response: As above, we attempted to generate *NANOG* null mutant naïve human ESCs and despite several attempts were unsuccessful. Notably, a previous paper from Ge Guo suggests that loss of NANOG in naïve hESCs leads to no observable colonies in N2B27 which are conditions similar to ours (Supplementary Figure 5, Guo et al., Cell Stem Cell 2021). The group also demonstrate that loss of NANOG leads to TE-like differentiation. However, the genotype of the targeted cells was not provided and there was not extensive characterisation beyond GATA3 upregulation. Investigating the role of NANOG in naïve human ESCs warrants further technical optimisation and is beyond the limit of our technical ability.

5. The authors suggest IGF1 signaling compensates for loss of NANOG/FGF4 for hypoblast specification in humans. This could be directly tested by inhibiting IGF1R in NANOG-deficient embryos. Mouse embryos should be used as controls. Conversely, combining FGF blockade with IGF1 supplementation in WT mouse and human embryos could assess functional redundancy.

Response: As noted above we have since removed the emphasis on IGF1 and instead provide alternative factors that may functionally compensate for the loss of FGF4 in primitive endoderm specification.

To address the question of whether IGF1 signalling compensates for the loss of NANOG/FGF4 in human would involve IGF1R and insulin receptor inhibition in *NANOG* null mutant embryos compared to controls. This would require approximately 30 oocytes to have sufficient numbers of replicate embryos that reach the blastocyst stage given drop off in fertilisation and blastocyst development rates. We unfortunately do not have sufficient human gametes consented to perform this experiment. We include a suggestion to investigate the functional compensation and interplay between IGF and FGF signalling in the manuscript noting:

"We previously showed crosstalk between FGF and IGF signalling in stem cell proliferation and that treatment with exogenous IGF1 led to increased numbers of ICM cells with embryos in a diapause-like state (Iyer et al., Cell 2024; Wamaitha et al., Nature Communications 2020), it would therefore be interesting to determine if FGF18, IGF1 or alternative RTK signalling components can compensate for the loss of *NANOG* and whether they may have a role in primitive endoderm specification in a non-perturbed context."

“In contrast to the mouse, we observed that *NANOG*-edited human embryos retained primitive endoderm cells despite the absence of FGF4 and our data suggests a number of plausible RTK signalling components that would be interesting to investigate in the future.”

As noted above we have removed the IGF1 data because we were not able to repeat this experiment. We have instead addressed the point of Reviewer 3 by providing plausible alternative factors that may functionally compensate for the loss of FGF4.

5. Given the availability of protocols for culturing human blastocysts beyond implantation (PMIDs: 27144686, 27144363, 31830756), the authors should consider extending culture and analysis of NANOG-edited and control embryos into early post-implantation stages.

Response: The focus of our study is on the role of NANOG in human preimplantation lineage specification. We observe that NANOG null-mutations lead to loss of epiblast specification prior to implantation at the blastocyst stage. We therefore do not believe extended culture will be informative to understanding the role of NANOG in preimplantation development because we already see a completely loss of epiblast prior to implantation. There are roles of other factors we seek to investigate after implantation, but NANOG is not one of these factors. Moreover, current post-implantation culture conditions are not yet robust and reproducible to determine the role of factors in human early epiblast development.

Minor concerns:

1. To confirm that observed phenotypes are due to NANOG loss, please perform a rescue experiment by reintroducing NANOG in human ESCs.

Response: We believe that a rescue experiment is beyond the scope of this study. It is also not the standard method used in the field, including in evaluating the role of NANOG in mouse embryos or in ESCs (e.g. Chambers et al., Cell 2003; Mitsui et al., Cell 2003; Silva et al., Cell 2009; Chambers et al., Nature 2007; Zhang et al., Cell Reports 2018; Frankenberg et al., Developmental Cell 2011; Allegre et al., Nature Communications 2022). We are confident in the results because we were able to reproduce data from the genetic knock-out mouse showing loss of primitive endoderm development (consistent with Frankenberg et al., Developmental Cell 2011; Allegre et al., Nature Communications 2022) and provided additional phenotypic characterisation that has not been performed before, for example scRNAseq. We also confirmed and expanded on the *NANOG* loss-of-function phenotype in human ESCs (consistent with Vallier et al., Development 2009) again giving us confidence in our data and conclusions.

2. Co-injection of CRISPR constructs together with sperm into M-II oocytes has previously been used to reduce mosaicism (PMID: 28783728). The authors should cite this paper and briefly comment on this.

Response: We appreciate the reviewer's point and have included the following sentence in the results section: "We used a co-injection method that has previously been shown to reduce mosaicism and increase the efficiency of CRISPR-Cas9-mediated editing (reference PMID: 28783728)".

3. Include a volcano plot, analogous to Fig. 3f, to illustrate the transcriptional impact of Nanog knockout in mouse blastocysts.

Response: We appreciate the Reviewer's helpful suggestion and have included the mouse volcano plot in Extended Data Fig. 13a.

4. Show the effect of IGF1 on primitive endoderm formation in mouse embryos to complement the human data presented in Fig. 4f.

Response: As noted above we have removed the IGF1 treatment data and expanded on alternative mechanisms that may functionally compensate for the absence of FGF4-mediated primitive endoderm differentiation.

5. Please specify how cell types (e.g., trophectoderm, epiblast, ICM, primitive endoderm) were classified in both human and mouse single-cell RNA-seq datasets.

Response: We appreciate the Reviewer's point, which was also raised by other reviewers. In the human datasets, cells from each of the lineages clustered separately and the trophectoderm was distinguished by molecular markers *GATA2*, *GATA3*, *CDX2*, the inner cell mass expressed *GATA6* and *POU5F1*, the epiblast was enriched for *NANOG*, *POU5F1* and *ARGFX*), while the primitive endoderm expressed *GATA6*, *GATA4* and *PDGFRA*. These data are presented in Figure 4b,e). We also provide a count matrix table (Supplementary Table 5) annotating the lineage associated identity of each individual cell.

In the mouse, the cell types were initially distinguished by the expression of trophectoderm markers Cdx2 and Gata3, epiblast by the expression of Nanog and Sox2 and primitive endoderm by the expression of Gata6 and Sox17 (Extended Data Fig. 8d). We subsequently confirmed these assignments using dot plots for additional markers presented in Extended Data Fig. 8e. We have used similar strategies to assign lineages in both human and mouse in past publications (Blakeley et al., Development 2015) and our strategy for lineage assignment was confirmed by other studies (e.g. Stirparo et al., Development 2018).

6. Clarify whether the reported 77.4% efficiency for sgRNA5 (Fig. 1c, Extended Data Fig. 1b) was calculated post-transfection and sorting.

Response: We appreciate this point and clarified in the figure legend that the bulk population of cells were analysed post transfection and puromycin selection.

7. Line 90–91 should state that both N-terminal and C-terminal antibodies failed to detect NANOG protein instead of claiming "full-length loss of NANOG protein".

Response: We appreciate this point and amendmented the text accordingly.

8. For single-cell RNA-seq data, subdivide edited alleles into synonymous, missense, and other categories. Provide detailed read-level sequences in the supplementary data.

Response: We have since updated our method to genotyping the embryos prior to phenotyping. We provide a summary of the embryo genotypes in new Figure 3b.

We have removed reference to genotypes based on RNA-seq data. We provide examples of transcriptional reads into the intron, which confirms there is a splicing defect. There is no synonymous or missence mutations in this case because the edits are in the splice site, not within the exon.

9. For embryos that failed to reach the blastocyst stage (Extended Data Fig. 3b), clarify whether editing efficiency was assessed at the single-blastomere level or not.

Response: We appreciate the reviewer's point and have revised the figure legend to make it clear that the genotyping was performed on whole arrested embryos. We have included additional replicate whole embryos, which has increased the number from 5 to 16 embryos analysed. A summary of our findings are presented in Extended Data Fig. 5b-d.

10. In Extended Data Fig. 9, INSR and IGF1R appear only modestly enriched in hypoblasts. Please provide IF evidence of their expression in human hypoblasts and compare with mouse embryos.

Response: As noted above we have since removed the data related to IGF1 treatment of human embryos and provided alternative factors that may functionally compensate for the loss of FGF4.

We find that INSR and IGF1R are transcribed in ICM, TE and EPI cells and INSR is also expressed in some PrE cells in the control embryos. It may be there is a cell-fate switch of ICM or EPI cells to PrE or a proliferative advantage to PrE cells. It would be interesting to explore this mechanism in the future, which is beyond the scope of our study. We did not evaluate protein expression by immunofluorescence analysis. It is challenging to evaluate receptor expression in the primitive endoderm using immunofluorescence analysis because our standard methods require permeabilization which will lead to protein extraction, disruption of receptor complexes and loss, denaturation and epitope masking. To determine whether the protein for the receptor is expressed we would need to optimise methods for non or mild permeabilization. We would also need to evaluate whether the presence or absence of the trophectoderm could impact on antibody accessibility to receptor epitopes. We felt that it is beyond the scope of the paper to optimise a new method for immunofluorescence analysis, which has never been used in the lab. From previous studies we did observe by western blot analysis previously the expression of downstream effectors of the INSR and IGF1R pathways including expression of phosphorylated AKT (pAKT) and S6 (pSK) along with pan AKT and pan S6 (Wamaitha et al., 2020).

Referee #5 (Remarks to the Author):

I co-reviewed this manuscript with one of the reviewers who provided the listed reports.

Response: We understand that the peer reviewer has provided a joint report with another reviewer.

We are grateful for an opportunity to submit a revised manuscript. We are also very grateful that all reviewers were positive about our previously revised manuscript. We appreciate that there was an additional reviewer who provided constructive feedback. We have addressed all of the remaining recommendations and feel that these were helpful and constructive. We addressed the reviewer comments by providing the following changes:

1. Clarify how we present and explain the splicing defect observed. We have revised the scRNAseq data mapping to the *NANOG* locus on an IGV browser to show clear GT→GC mutation at the *NANOG* splice donor site, aberrant intron-retaining transcripts following exon 1 and absence of mapping to subsequent exons, suggesting a splicing defect (Extended Data Figure 5). This is in contrast to *AAVS1*-edited controls where scRNAseq reads mapped to the 3' untranslated region and all *NANOG* exons with evidence of correct splicing across exonic regions. We also contrast this with reads mapping to the *GAPDH* locus in both the *NANOG*- and *AAVS1*-targeted cells which showed correct splicing and reads mapping to only exons.

2. Revised the discussion section to emphasize that the focus of our study is the development and application of an effective method to investigate gene function in early human embryos. We have made it clearer that substantial basic research is necessary before potential therapeutic correction including further assessment of off-target activity, genotoxicity, and developmental potential in a larger cohort of embryos and across more genomic target loci.

3. Amended the human embryo immunofluorescence figures to distinguish the homozygous mutant embryos from the mosaic/heterozygote NANOG-targeted embryos (now in Figure 1).

4. Amended the manuscript text to address the points raised about terminology and typographical errors.

5. To address the journal formatting instructions, we have revised the number of main and extended data figures. We now provide 4 main and 9 extended data figures and have reduced the manuscript text.

We would be happy to make any further changes if needed.

Referees' comments:

Referee #1 (Remarks to the Author):

The authors have addressed my questions to my satisfaction - the additional data and analyses have improved the manuscript.

One suggestion: on p15 the authors end with a statement about the conditions of legitimate use of embryo genome editing in a clinical context: "Nonetheless, clinical translation of genome editing in human embryos must not proceed unless there is broad societal debate and support" - it would be useful to have a reference to the reports of the two commissions on Human Genome Editing at this point, one of which focussed on heritable uses, since they remain useful reminders of this and other conditions. The citations are:

National Academies of Sciences, Engineering, and Medicine (2017) Human Genome Editing: Science, Ethics, and Governance. Washington, DC: The National Academies Press. doi: https://doi.org/10.17226/24623.

National Academy of Medicine, National Academy of Sciences, and the Royal Society (2020) Heritable Human Genome Editing. Washington, DC: The National Academies Press. https://doi.org/10.17226/25665.

Andy Greenfield

Response: We thank the reviewer for this helpful contribution and for their constructive feedback which strengthened the manuscript. We have added the relevant citations, as well as a citation for the updates to the ISSCR Guidelines for Stem Cell Research and Clinical Translation (Lovell-Badge et al., 2021). We believe these citations complement our emphasis that broad societal debate is necessary in considering whether or not to progress with clinical translation.

Referee #2 (Remarks to the Author):

The authors have done more experiments and analyses to improve this study and the concerns I raised have been addressed. I recommend the acceptance of this revised manuscript.

Response: We thank the reviewer for their constructive feedback which considerably improved our study.

Referee #3 (Remarks to the Author):

The revised manuscript has improved substantially following the reviewers' comments. In particular, the authors have increased the number of edited embryos analysed, introduced genomic genotyping of embryos, added immunofluorescence validation of NANOG protein loss and lineage markers, and revised the transcriptomic analyses accordingly. These additions strengthen the central conclusions.

Overall, the study is now considerably stronger and many of the initial concerns have been addressed. However, several points still require clarification or moderation in the manuscript.

Response: We thank the reviewer for their constructive feedback which improved our study and we appreciate the additional points below which we have attempted to clarify with revisions to the text and amendments to figures.

***1.** Evidence for disruption of NANOG splicing: The authors now provide additional evidence suggesting disruption of the NANOG splice donor site, including RNA-seq reads extending into the intron and PCR assays across exon boundaries. Together with the immunofluorescence analysis showing loss of NANOG protein, this provides support for functional disruption of the gene. However, the PCR data presented in Extended Data Fig. 7 appear to show faint bands in the edited samples, suggesting that amplification may not be completely absent. This raises the possibility that low levels of correctly spliced transcript could still be present. The authors should therefore clarify this interpretation and avoid stating that no amplification occurs if weak bands are visible. More broadly, the RNA-level consequences of the splice-site mutation remain somewhat indirect. Visualization of splice junction usage or more explicit characterization of aberrant transcripts would further strengthen the conclusion that the GT→GC mutation produces a complete loss-of-function allele.*

Response: We agree that the PCRs presented in the Extended Data Figure 7 were ambiguous, as faint bands were detectable in the edited samples and could be interpreted as residual correctly spliced transcript. We had attempted Sanger sequencing of excised bands from the gels, however sequencing came back inconclusive and we could not verify by sequencing that the target amplicon is the *NANOG* locus, an intronless *NANOG* pseudogene or another genomic loci. To avoid overinterpretation, we have removed these PCRs from the revised manuscript.

Instead, we have amended our scRNAseq visualisation to more directly assess splicing outcomes at the *NANOG* locus in Extended Data Figure 5. We have updated the figure to show read alignments at the *NANOG* exon–intron boundary, demonstrating clear extension of reads mapping from exon 1 to intron 1 in *NANOG*-targeted cells and absence of reads mapping to exons 2-4, consistent with disruption of the canonical splice donor site. Together with the loss of NANOG protein by immunofluorescence, these data support the conclusion that the GT→GC mutation disrupts normal splicing and results in intron-retention and prematurely terminated mRNA in *NANOG* edited cells. In AAVS1 and non-edited controls there is no retention of *NANOG* intronic reads and the mRNA only maps to the exonic or 3' untranslated regions. We also contrast this with reads mapping to the *GAPDH* locus in both the *NANOG*- and *AAVS1*-targeted cells which showed correct splicing and reads mapping to only exons.

We have revised the section, now on page 11, to the following:

'In *NANOG*-edited cells, scRNAseq reads exhibited GT to GC mutation at the *NANOG* splice donor site, aberrant intron 1-retaining transcripts following exon 1 and absence of reads mapping to exons 2-4, suggesting a splicing defect (Extended Data Fig. 5). By contrast, in *AAVS1*-edited controls, *NANOG* scRNAseq reads mapped to the 3'

untranslated region and all *NANOG* exons, with evidence of correct splicing across exonic regions (Extended Data Fig. 5).'

We would be happy to amend this further if the reviewer felt this would be essential.

2. *Interpretation of editing efficiency and mosaicism: The authors now genotype embryos using Sanger sequencing of multiple trophectoderm biopsies and identify embryos classified as homozygous edited. This represents an important improvement compared with the original submission. However, genotype assignment based on a limited number of biopsies cannot fully exclude mosaicism within the remaining embryo. Consequently, it may be premature to conclude that embryos showing homozygous edits in all biopsies represent completely edited embryos. This limitation should be acknowledged more explicitly. Related to this point, statements that mosaicism is reduced or that embryos are fully edited should be carefully phrased given that the analysis is based on biopsies rather than whole-embryo genotyping.*

Response: We thank the reviewer for identifying this important caveat to our analysis strategy. We have more explicitly highlighted this limitation in revised text on page 11:

'Genotyping of biopsied embryos indicated homozygous mutant, heterozygous or mosaic editing, or wildtype alleles at the *NANOG* locus (Fig. 2a). While these groupings informed further analysis, trophectoderm biopsies represent only a portion of the embryo and may not reflect the genotype of the embryo as a whole.'

Further down on page 11:

'To further understand the transcriptome-wide consequences of the loss of *NANOG* we focused on the embryos where trophectoderm biopsies suggested a homozygous *NANOG* mutant genotype compared to *AAVS1* and reference control embryos.'

We would be happy to make further edits if the reviewer felt this would be important.

3. *Use of arrested embryos to estimate editing efficiency: The authors include analysis of embryos that arrested prior to the blastocyst stage in order to estimate editing efficiency, which is a good idea in se. However, interpretation of editing outcomes in arrested embryos is not straightforward. Arrested embryos frequently exhibit DNA fragmentation or developmental abnormalities, which may complicate interpretation of genotyping results. The authors should clarify the rationale for including these embryos in the analysis and avoid overinterpreting editing efficiency based on this dataset.*

Response: The reviewer raises an important point that we agree with. We have included the following clarification and caveat:

'To maximise the information gained from embryos used in this study, arrested human embryos were also analysed to evaluate editing frequency (Extended Data Fig. 3g-i). Sequencing of embryos that arrested prior to the blastocyst stage revealed an editing frequency of 62.5% (n = 10 out of 16 embryos) (Extended Data Fig. 3i). However, given that arrested human embryos are developmentally compromised and frequently harbour karyotypic abnormalities[30], this editing rate may not be representative of embryos capable of developing beyond this stage.'

Some minor comments:

***1.** The immunofluorescence analysis demonstrating loss of NANOG protein together with retention of GATA3 and GATA4 is an important addition and provides independent validation of the single-cell RNA-seq results. This experiment substantially strengthens the manuscript.*

Response: We thank the reviewer for their supportive comment.

***2.** The manuscript occasionally refers to the editing strategy as achieving "high-efficiency disruption" of NANOG. Given that complete loss of NANOG protein is observed in a subset of embryos, it may be more accurate to moderate this wording.*

Response: We appreciate the reviewers point and agree. We have removed mentions of high-efficiency (or efficient) editing of human embryos, instead describing it as effective where we thought it was appropriate. Our reasoning is based on the observation that 84.8% of trophectoderm biopsies (n = 39/46) and 100% of embryos (n = 12/12) (Figure 2a) had evidence of editing at the *NANOG* locus. Of these, 25% had homozygous edits at the *NANOG* locus (n = 3/12 embryos). We therefore concluded that the method was sufficiently effective to allow us to investigate gene function. Moreover, we observed >75% *NANOG* editing in transient transfections of human embryonic stem cells. In the mouse, we observed consistently >80% editing rate of the *Nanog* locus. We would be happy to amend this further if necessary.

***3.** In the description of the editing experiments, the text refers to "delivery 11 of ABE8e protein". This appears to be a typographical error and should be corrected.*

Response: We thank the reviewer for identifying this typo. We have since revised the manuscript and amended this sentence.

Referee #6 (Remarks to the Author):

In this revised ms, the authors describe the use of base-editing tools to assess the function of the Nanog transcription factor in early human embryos. Overall, as noted by the previous reviewers (I was not one of the previous reviewers), they have provided a careful assessment of the effectiveness of adenine base editing to target a splice donor site in Nanog, which should produce a splicing defect and effectively a null mutation. They tested it first in human ES cells and then in preimplantation human embryos. In an extended series of human embryo studies, they report an editing frequency of 62.5% (10/16 embryos). However, only 3 embryos were homozygous Nanog-/-. The rest were presumably heterozygous or mosaic- thus the overall efficiency of editing is not quite clear.

In the three homozygous embryos, they find reduction in epiblast markers but presence of primitive endoderm and trophectoderm, suggesting that loss of Nanog leads to failure of epiblast development but not of the primitive endoderm. This is in contrast to the mouse, where loss of Nanog leads to loss of both epiblast and primitive endoderm,

identifying a clear distinction across the species, likely related to the loss of FGF4 in the mouse and some resistance to this loss in humans.

They also provide some analysis of any possible off target editing at either the DNA or RNA level, as well as evidence of low likelihood of aneuploidy.

Overall, it seems that base editing is an appropriate tool for mutational analysis of gene function in early human embryos. The efficiency is reasonable, and any genotoxic effects are reduced compared with CRISPR/Cas9. This study is not designed to assess whether this approach could be used clinically, which would require a lot more analysis than provided here. The authors must make this distinction very clear. On l348 they state that they have established a framework for potential therapeutic correction – I do not believe this is the case-and it is NOT the goal of this study. I would rewrite this section.

Response: We thank the reviewer for their valuable feedback and very helpful comments. We completely agree that our study is not designed to assess whether the approach could be used clinically and that much more basic research would be needed. We have revised the section on pages 15 and 16 to:

'Given that G•C>A•T substitutions underlie nearly half of all known pathogenic variants[5], base editing approaches have been proposed as potential tools for disease modelling and therapeutic correction. While the present study was not designed to assess clinical applicability of base editing in the human germline, our findings do demonstrate reduced aneuploidy following genome editing compared to previous Cas9-mediated approaches[1-3]. While these observations suggest potential for base editing to investigate gene function in early-stage human embryos, substantial basic research is necessary before potential therapeutic correction. This includes extensive evaluation of safety, such as further assessment of off-target activity, genotoxicity, and developmental potential in a larger cohort of embryos and across more genomic target loci in basic research before consideration for clinical use in heritable genome editing.'

Specific Comments

***1.** L86 and elsewhere. It is stated that the base editing leads to splicing defects and a null allele. It is not quite clear to me exactly what the null allele would be- can this be explained better? Is NMD induced? Has this been assessed? Or is the splicing defect alone enough to produce a null allele?*

Response: We agree with the reviewer that this is confusing and we have since revised the manuscript to remove reference to a null allele. We have revised the figure containing scRNAseq reads mapping to the *NANOG* locus on an IGV browser to show clear read-through from exon 1 into intron 1 in *NANOG*-targeted cells (Extended Data Figure 5). On page 11 we have amended the text to explain that scRNAseq reads in *NANOG*-targeted cells exhibited GT→GC mutation at the *NANOG* splice donor site, aberrant intron-retaining transcripts following exon 1 and absence of mapping to subsequent exons, suggesting a splicing defect (Extended Data Fig. 5). This is in contrast to *AAVS1*-edited controls where scRNAseq reads mapped to the 3' untranslated region and all *NANOG* exons with evidence of correct splicing across exonic regions. We also contrast this with reads mapping to the *GAPDH* locus in both the *NANOG*- and *AAVS1*-targeted cells which showed correct splicing and reads mapping to only exons.

While we have not directly assayed NMD in this study, the scRNAseq data, together with the absence of detectable NANOG protein by immunofluorescence, support the conclusion that the edited allele behaves as a functional loss-of-function (null) allele.

We have revised the section, now on page 11, to the following:

'In *NANOG*-edited cells, scRNAseq reads exhibited GT to GC mutation at the *NANOG* splice donor site, aberrant intron 1-retaining transcripts following exon 1 and absence of reads mapping to exons 2-4, suggesting a splicing defect (Extended Data Fig. 5). By contrast, in *AAVS1*-edited controls, *NANOG* scRNAseq reads mapped to the 3' untranslated region and all *NANOG* exons, with evidence of correct splicing across exonic regions (Extended Data Fig. 5).'

2. *It is hard to work out the exact effect of loss of Nanog on the overall development of the blastocysts. In Fig 2 e-j, we are given cell number counts for different lineages in the edited blastocysts. Overall it does not seem that there is a big difference in overall cell number between wt and edited embryos (Fig2e) and the number of TE and PrE cells do not seem to differ. However, these numbers pool all edited embryos including non-homozygous mutants, making it hard to know what is happening. The most important data must come from the homozygous mutants- and these are presumably the three embryos in Fig 2j that show only GATA4+ cells in the ICM. However, this figure does not provide actual cell counts, just percentages. Thus I am still unclear whether the ICM maintains its cell number but Epiblast cells are redirected to PrE or TE, or whether the overall ICM number is reduced as epiblast cells fail to develop, but PrE and TE cell numbers do not change. These different scenarios lead to different interpretations of the phenotype observed.*

Response: The reviewer makes several excellent points. We have revised the figure (now Figure 1e-i) to make it clear the total n number of cells that express each of the lineage-associated molecular markers and total cell number for each embryo. We have also distinguished the homozygous mutant embryos (lighter shade compared to other samples) from the mosaic/heterozygote *NANOG*-targeted embryos. The data suggests that there is a trend toward lower cell number in the *NANOG* homozygous mutant blastocysts compared to controls and mosaic/heterozygote embryos. The ICM number is reduced in *NANOG* homozygous mutant embryos, GATA4 expressing primitive endoderm cells are observed, but this does not appear to be a conversion of epiblast cells because NANOG expressing epiblast cells fail to develop and the ICM number is reduced. This is interesting and in contrast to what we and others observed for FGF-treated embryos where there is a conversion of NANOG-expressing epiblast cells to GATA4-expressing primitive endoderm and no reduction in the number of ICM cells (see Figure 1 and Supplementary Figure 1c in Simon et al., Nature Comms 2025). There is also a trend toward fewer GATA3 expressing TE cells in *NANOG* homozygous mutant embryos. We would be happy to expand on these points in the manuscript if the reviewer felt that this would be appropriate. We are a little reluctant to draw too many conclusions in the manuscript from a low n number.

***3.** Terminology needs to be adjusted. In the Abstract, l52, the authors talk about yolk sac or placental-like transcriptional programs, when what they mean is primitive endoderm (hypoblast) and trophectoderm lineage programs. Yolk sac and placenta are multi-lineage organs.*

Response: We agree with the reviewer and have revised the abstract and manuscript accordingly.

***4.** Another confusing use of terminology is to call the dissected part of the blastocyst that contains the polar TE and the ICM as the ICM. It actually has all three lineages in it and the single cell RNAseq data has to be segregated to identify the three cell types. I think the previous reviewers were quite confused and so will be the readers of the paper. Please rename this dissected part of the blastocyst- perhaps polar region?*

Response: We thank the reviewer for this helpful clarification and suggestion. We have amended the text throughout to refer to this as the polar region and defined this as comprising epiblast, primitive endoderm and overlying polar trophectoderm cells.

***5.** L 331 states that the results demonstrate failure to activate epiblast-associated program. Can this actually be demonstrated? It might require looking in earlier stages of development of Nanog -/- embryos. I realize this is not a reasonable request but would suggest some caution in interpreting the current results.*

Response: We completely agree with the reviewer, to demonstrate this would require considerable investigation of *NANOG-/-* human embryos at earlier stages of development. We are very grateful for the opportunity to amend this section and have since removed this sentence.
