# Supplementary material for: Base editing reveals an essential role for NANOG in human embryogenesis
Source: Nature. 2026 Jun 25;658(8138):1281–8. doi: 10.1038/s41586-026-10792-1 (PMC13716833; doi:10.1038/s41586-026-10792-1)
Supplement: Supplementary file 3 [file 41586_2026_10792_MOESM7_ESM.docx]

**SUPPLEMENTARY TABLES 6 - 9**

**Base editing reveals an essential role for NANOG in human embryogenesis**

Oliver J. Bower, Ana E.R. Orsi, Riley McMahon, Desislava Staneva, Josephine Blagrove, Kashish Singh, Claire S. Simon, Afshan McCarthy, Patricia Garcia, Valerie Shaikly, Mohamed Taranissi, Martin Wilding, Paul Serhal, Rabi A. Odia, Mina Vasilic, Meenakshi Choudhary, Athanasios Papathanasiou, Kay Elder, Phil Snell, Leila Christie, Mandana Arbab, David R. Liu, Mary Herbert, Katarina Harasimov, Kathy K. Niakan

### **Supplementary Table 6**

### **sgRNA sequences**

| Name | Species | Sequence |
| --- | --- | --- |
| AAVS1 ABE8e sgRNA | Human | GCGGCTCCAATTCGGAAGTG |
| NANOG sgRNA ABE8e sgRNA | Human | TTCTTACCAGTCTCCGTGTG |
| Aavs1 sgRNA | Mouse | GCGATGCTGCAAAAAATGCA |
| Nanog sgRNA | Mouse | TACTGAGATGCTCTGCACAG |

### **Supplementary Table 7**

### **Sequencing primers**

| Name | Species | Sequence |
| --- | --- | --- |
| NANOG sgRNA Sequencing primer F Exon 1 | Human | TGTGATTTGTGGGCCTGAAG |
| NANOG sgRNA Sequencing primer R Intron 1 | Human | GGTCCATCATTGCTCAAGAGG |
| AAVS1 Sequencing primer F | Human | ATCCTCCTTCCCCGTTGC |
| AAVS1 Sequencing primer R | Human | ATGCGGAAGAGAGTAGGTCG |
| Nanog sgRNA Sequencing primer F | Mouse | AAGCATCGAATTCTGGGAACG |
| Nanog sgRNA Sequencing primer R | Mouse | AAGCAATAACCCTTCAGCCC |
| NANOG sgRNA Sequencing primer R Exon 4 | Human | CTGGATGTTCTGGGTCTGGT |
| NANOG sgRNA Sequencing primer R Exon 2 | Human | GCTGGGTGGAAGAGAACACA |
| GAPDH Primer F | Human | ATCCCTGAGCTGAACGGGAA |
| GAPDH Primer R | Human | ACCACCTGGTGCTCAGTGTA |
| Off-target: intergenic:LYZL2-RP11-14C22.3 Sequencing primer F | Human | TGGTGCTGAGGCTTGAAACA |
| Off-target: intergenic:LYZL2-RP11-14C22.3 Sequencing primer R | Human | CTGCCAAGACATCCCCACAT |
| Off-target: intron:SYNJ2 Sequencing primer F | Human | TCTGACACAGTGCAGGGAAAA |
| Off-target: intron:SYNJ2 Sequencing primer R | Human | GAAATGCACCTGGGACTGTTG |
| Off-target: intergenic:RNU6-1249P-TMEM100 Sequencing primer F | Human | AGTCCAGAAAAACTGAGTGTTGG |
| Off-target: intergenic:RNU6-1249P-TMEM100 Sequencing primer R | Human | GAGAGGGTGAGCATCTACCAC |
| Off-target: intergenic:RP11-415G4.1-RN7SKP6 Sequencing primer F | Human | GTACGAAGGTGCCCTTTATCCT |
| Off-target: intergenic:RP11-415G4.1-RN7SKP6 Sequencing primer R | Human | AATGACTCCCCAGGTGACAAG |
| Off-target: exon:MIR485 Sequencing primer F | Human | TCCCTTAAGGTAAGTGCGCC |
| Off-target: exon:MIR485 Sequencing primer R | Human | CCCTCCGAGGCAGAATTTGA |
| Off-target: intron:TXNDC16 Sequencing primer F | Human | GTGCCCCGTTTTTCTCCTTC |
| Off-target: intron:TXNDC16 Sequencing primer R | Human | AGTGGTAGGGGTTTAATGCCTG |
| Off-target: intergenic:LINC00659-MRGBP Sequencing primer F | Human | TGTCCATGTACGTGTGTTGT |
| Off-target: intergenic:LINC00659-MRGBP Sequencing primer R | Human | GACCCAGGGTGCTCAGAAT |
| Off-target: intron:GADL1 Sequencing primer F | Human | ATCTCTTGATAGTCTCCCCTGTCT |
| Off-target: intron:GADL1 Sequencing primer R | Human | ACTGTGGCCAAAGACAAAGTG |
| Off-target: intergenic:NANOGP1 Sequencing primer F | Human | TAAAGCCTGCCTTGCTCCAA |
| Off-target: intergenic:NANOGP1 Sequencing primer R | Human | CAGTGCACACTCCTGAGACC |
| Off-target: intron:AUH Sequencing primer F | Human | TAGGTCACACATCTGGCAGC |
| Off-target: intron:AUH Sequencing primer R | Human | AGCATTCTGTAAAGGGCCCT |
| Off-target: intron:Slc6a5 Sequencing primer F | Mouse | CCCTGTCACCTTCCAGACTT |
| Off-target: intron:Slc6a5 Sequencing primer R | Mouse | GCAATGCCACAACCTGCAAA |
| Off-target: intergenic:Gm5670-1700112E06Rik Sequencing primer F | Mouse | CAGGTCACACACTGCCTTTCT |
| Off-target: intergenic:Gm5670-1700112E06Rik Sequencing primer R | Mouse | GACCACTGGTGGAGAGCTGA |
| Off-target: intron:Nfia Sequencing primer F | Mouse | TGGGCTACGTGTAGATTGCC |
| Off-target: intron:Nfia Sequencing primer R | Mouse | CCCACGTAGAAAGAGCTGAGT |
| Off-target: intron:Ccnyl1 Sequencing primer F | Mouse | GCACTAAGTGGTTGGCTGGA |
| Off-target: intron:Ccnyl1 Sequencing primer R | Mouse | CCGGCTGTTCCAGAAGTAGT |
| Off-target: intergenic:Xylt1-Gm24063 Sequencing primer F | Mouse | CAAGACTCTGCCCCTCTTGTT |
| Off-target: intergenic:Xylt1-Gm24063 Sequencing primer R | Mouse | TGGGGAGACGAAGGAAATCCA |
| Off-target: intron:Nrcam Sequencing primer F | Mouse | GTACGTTGAGCTTACTTGTCCT |
| Off-target: intron:Nrcam Sequencing primer R | Mouse | CACTTCCCCACTCCCATACC |
| Off-target: intron:Corin Sequencing primer F | Mouse | TTGCCCTGCACAGCAGTAAT |
| Off-target: intron:Corin Sequencing primer R | Mouse | CCTTCTAGCATGGGGCTTGT |
| Off-target: intergenic:Tmem18-Fam150b Sequencing primer F | Mouse | TAGCACTTAGGGGGTGGAGTA |
| Off-target: intergenic:Tmem18-Fam150b Sequencing primer R | Mouse | GGTCTCCTTGTTTTTGCCACT |
| Off-target: intergenic:Sphkap-Gm25754 Sequencing primer F | Mouse | TAAGGACAGATTACGGACGCAG |
| Off-target: intergenic:Sphkap-Gm25754 Sequencing primer R | Mouse | AACTGAACAGCAGACATCCCT |

### **Supplementary Table 8**

### **Primary antibodies used in immunofluorescence**

| Target | Species | Dilution | Supplier | Catalogue No. |
| --- | --- | --- | --- | --- |
| Anti-OCT4 | Mouse IgG2b | 1:200 | Santa Cruz | SC-5279 |
| Anti-NANOG | Goat | 1:200 or 1:250 | R&D | AF1997 |
| Anti-NANOG | Rabbit | 1:100 | Abcam | ab21624 |
| Anti-NANOG | Rat | 1:100 | eBioscience | 14-5761-80 |
| Anti-GATA4 | Rabbit | 1:50 | Santa Cruz | SC-9053 |
| Anti-GATA4 | Rat | 1:400 | eBioscience | 14-9980-82 |
| Anti-CDX2 | Mouse | 1:100 | Biogenex | MU392A-UC |
| Anti-GATA3 | Rabbit | 1:200 | Abcam | ab199428 |

### **Supplementary Table 9**

### **Secondary antibodies used in immunofluorescence**

| Target | Dilution | Supplier | Catalogue No. |
| --- | --- | --- | --- |
| Donkey anti-Mouse IgG (H+L), Alexa Fluor | 1:300 or 1:400 | Invitrogen | A21202  A21203  A31571 |
| Donkey Alexa Fluor anti-Rabbit IgG (H+L), Alexa Fluor | 1:300 or 1:400 | Invitrogen | A21206  A21207  A31573 |
| Donkey Alexa Fluor anti-Goat IgG (H+L), Alexa Fluor | 1:300 or 1:400 | Invitrogen | A11055  A11058  A21447  A32816 |
| Donkey Alexa Fluor anti-Rat IgG (H+L), Alexa Fluor | 1:300 or 1:400 | Invitrogen | A21208  A21209  A48272 |
